# Data-Driven Generation of Conformational Ensembles and Ternary Complexes for PROTAC and Other Chimera Systems

**DOI:** 10.1021/acs.jcim.5c00880

**Published:** 2025-09-05

**Authors:** Fabio Montisci, Laura Friggeri, Kepa K. Burusco-Goni, Patrick McCabe, Bojana Popovic, Jason C. Cole

**Affiliations:** 42702 Cambridge Crystallographic Data Centre, 12 Union Road, Cambridge CB2 1EZ, U.K.

## Abstract

We present the protolysis-targeting chimera (PROTAC) Conformer Generator, a fast and knowledge-based tool for generating robust conformational ensembles of PROTACs and other chimeric degraders. The modeling protocol integrates conformer generation, rigid-body ternary complex (TC) assembly, and conformational sampling strategies that address the inherent flexibility and complexity of these molecules. Each modeled TC is evaluated using a clash-score and a surface-score, designed to prioritize sterically and geometrically plausible models with favorable protein surface interactions. The protocol was validated using experimentally determined PROTAC-mediated TC structures from the Protein Data Bank and “PROTAC-like” structures from the Cambridge Structural Database, demonstrating accuracy across diverse systems. The results show that the PROTAC Conformer Generator can reliably reproduce experimental conformations and generate simple TC models that recapitulate the relative orientations between E3 ubiquitin ligase and the protein of interest as observed in protein crystal structures. This robust validation supports the method’s reliability and establishes a reference framework for degrader modeling studies. The PROTAC Conformer Generator provides a structured and validated workflow for modeling and assessing degrader conformations and ternary complexes, enabling rapid ensemble generation and downstream integration into relevant early stage drug design pipelines.

## Graphical abstract

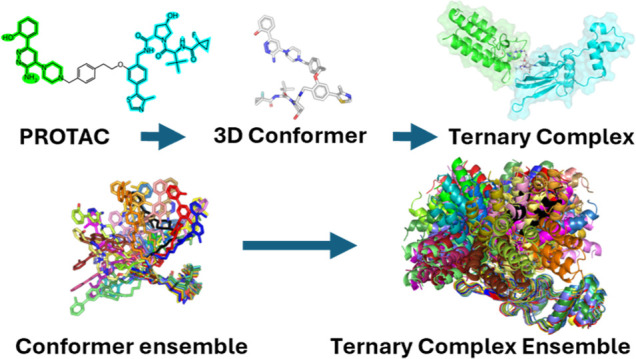

## Introduction

### Background and Current Landscape of PROTAC Modeling

Following the discovery of the first peptide degrader,^1^ scientists have dedicated significant effort to developing potent, selective, and safe proteolysis-targeting chimeras (PROTACs). PROTACs are heterobifunctional molecules that contain a warhead targeting a protein of interest (POI), an E3 ubiquitin ligase (E3) recruiting group, and a linker connecting the two moieties. Unlike traditional inhibitors that typically block the activity of a protein, PROTACs lead to the effective removal of the POI from the cell by hijacking the ubiquitin-proteasome machinery.^1–9^ The linker plays a critical role in mediating the assembly of a ternary complex (TC) with the necessary stability and orientation to enable proximity-induced ubiquitination and subsequent degradation of the POI.^7–9^

Since the first PROTACs entered clinical trials for the treatment of breast and prostate cancer in 2019,^10,11^ the opportunities for using these new therapeutic modalities to tackle undrugged targets have been widely recognized. For example, PROTACs have recently been explored for potential applications in infectious diseases^12,13^ and agriculture.^14^ This attention to PROTACs in the scientific community has led to a substantial body of literature. A database called PROTAC-DB was developed to catalog all the available information and is periodically updated.^15,16^ Several compounds have demonstrated promising results, with some already in or approaching phase III clinical trials; a recent article provides a summary of the ongoing trials.^17^

PROTAC technology represents an innovative approach to drug discovery,^17–28^ but it also challenges its conventional rules. For instance, typical PROTACs are well beyond Lipinski’s rule-of-five,^29,30^ which might suggest potential drug metabolism and pharmacokinetics risks. Their high molecular weight can lead to poor cellular permeability, solubility, oral bioavailability, and other drug-like properties, although these limitations could be partially mitigated by their ability to behave as molecular chameleons.^31–33^ They require slightly more complex synthetic routes than classical small-molecule drug discovery programs; they present an especially critical step in the linker optimization, responsible for driving potency (as in degradation efficiency) and selectivity, by determining the conformation of the TC.^7–9,34^

In the past few years, several PROTAC-mediated TC structures have been deposited in the Protein Data Bank (PDB),^35^ primarily determined by X-ray diffraction. Although the number of such structures remains limited, they provide valuable high-resolution insights into PROTAC functionality and can inform the design of more selective and drug-like chimeric compounds. Despite potential artifacts introduced by crystal packing and cryogenic conditions, X-ray diffraction remains the most widely available and highest-resolution experimental technique for characterizing these complexes. As such, it currently offers the most practical and informative benchmark for validating and developing new computational tools that need to be adjusted to effectively address these new larger modalities. Looking ahead, techniques such as cryoelectron microscopy (cryoEM), nuclear magnetic resonance (NMR) spectroscopy, and hydrogen/deuterium exchange mass spectrometry (HDX-MS) may offer complementary insights into PROTAC conformational dynamics.

Building on this structural foundation, several computational approaches to model PROTAC-mediated TCs have been reported. However, these modeling efforts remain relatively limited compared with the growing body of experimental studies. Pfizer scientists reported a computational workflow to model TCs with Bruton’s tyrosine kinase (BTK) as POI and Cereblon (CRBN) as E3, employing force-fields (FFs) and conformational searches.^36^ Drummond and co-workers published several methodological protocols to reproduce TC crystal structures using Molecular Operating Environment’s Scientific Vector Language.^37–40^ Other approaches have relied on protein–protein docking, like the study of Nowak et al.^41^ using RosettaDock,^42^ and, more recently, by Weng et al. using a combination of FRODOCK^43^ and RosettaDock with several filtering and rescoring steps.^44^ PRosettaC, a combined protocol taking both the protein–protein interactions (PPIs) and the PROTAC molecule conformational space into account, was presented in 2020.^45^ More recently, Li et al. combined this method with molecular dynamics (MD) and neural networks to try to predict which models would reward POI degradation.^46^ Other studies employed MD in combination with Molecular Mechanics-based methods, such as the General Born^47^ or Poisson–Boltzmann^48^ Surface Area approaches, to estimate binding energies and rescore docking poses. Finally, Wurz et al. recently reported a workflow highlighting the importance of co-operativity for TC formation^49^ and Li et al. a protocol for 3D-based linker design with reinforcement learning.^34^

Although all these computational protocols have certainly pushed PROTAC design capabilities forward, a higher predictive power and retrieval rate of crystal conformations across multiple POIs, E3s, and linkers would clearly be highly desirable. The challenges in the development of PROTAC-related computational tools come from several sources. One reason is the scarcity of reference crystal structures at our disposal and the crystallographic resolution of said structures. Another reason is extending pre-existing computational methods, which have been targeted and validated against more traditional therapeutic agents, to PROTAC-mediated TCs. For example, both protein–ligand and protein–protein docking were developed for other scenarios and, although still useful, are not perfectly suited for PROTACs. Finally, the problem is intrinsically complex due to the flexibility of linkers and the large number of rotatable bonds in PROTAC molecules, leading to a large conformational space that complicates the generation of a robust and diverse ensemble of putative TCs.

Nevertheless, computing and predicting accurate conformers are key steps for the in silico design of PROTACs. Indeed, determining correct and realistic torsion angles is crucial to modeling TCs with proximity between the POI and the E3, allowing favorable orientation for ubiquitination and degradation.^50^ Moreover, PROTAC conformations can also affect chemical properties such as solubility.^30^

### Objectives and Scope of This Study

To address the challenges outlined above, we developed a fast and knowledge-based approach focused on conformational ensemble generation and early stage TC modeling.

In this study, we present the PROTAC Conformer Generator (PCG), a Python tool based on the Cambridge Structural Database (CSD) Python API^51^ and the well-established CSD Conformer Generator.^52^ The PCG efficiently samples the conformational space of PROTACs and related chimeric degraders, by leveraging experimental torsional preferences from the CSD,^53^ which has been extensively used to validate ligand conformations and inform generative models.^54,55^ The PCG generates diverse and geometrically plausible linker conformations while keeping the E3 recruiting group and the POI warhead constrained to their protein-bound geometries.

While our primary goal was to generate conformational ensembles of PROTACs, we also implemented a simple rigid-body assembly of TCs. This was initially conceived as a filtering step to eliminate conformers that are likely to cause severe steric clashes or lack meaningful PPIs. However, we found that this approach often produced biologically plausible models, reproducing the POI-E3 relative orientation observed in the experimental crystal structures. This highlights the potential of PCG as a rapid and practical tool for generating starting models for further refinement. An overview of the PCG workflow is shown in Figure 1A–C.

**Figure 1:**
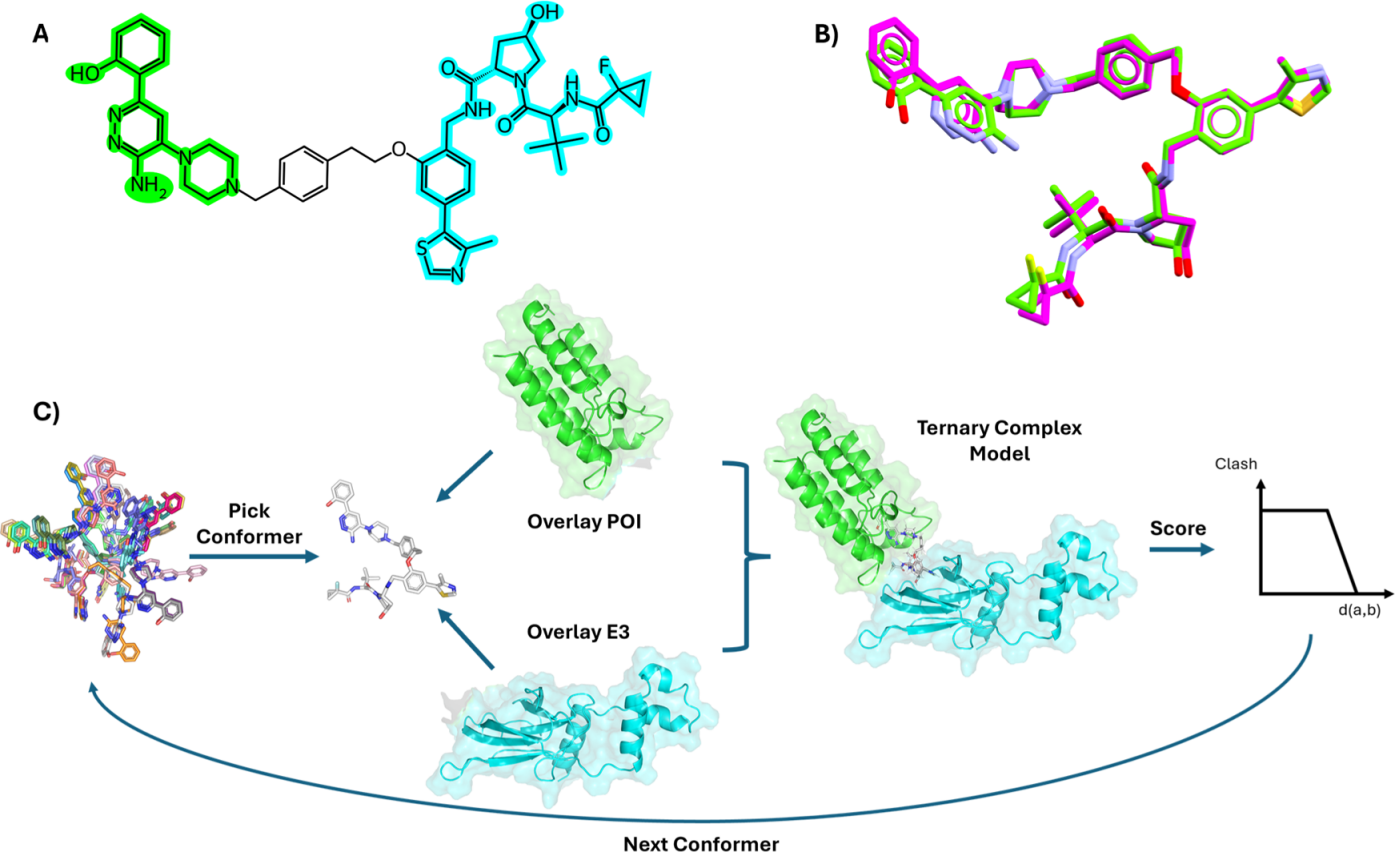
(A) Representation of a PROTAC molecule from Farnaby et al.^56^ (PROTAC2, corresponding to ligand FWZ from PDB structure 6HAX) that targets the bromodomain of human SMARCA2 and recruits the von Hippel-Lindau (VHL) E3. The warhead and E3 recruiting group are highlighted in green and cyan, respectively, and their internal coordinates are kept constrained to their protein-bound pose during conformer generation. (B) Superimposition of the FWZ ligand from 6HAX crystal structure (green) and the closest conformation generated by the PCG (magenta). (C) Schematic of the PCG workflow, showing the superimposition of the POI (green, PDB 6HAZ) and the E3 (cyan, PDB 5NVX) onto a conformer from the generated ensemble. The TC models are scored based on atom–atom pairwise distances.

To evaluate the performance of the PCG, we validated it using two complementary datasets: (1) a set of 34 experimentally determined PROTAC structures from the PDB and (2) a set of 116 “PROTAC-like” molecules from the CSD, selected based on geometric similarity to PROTACs, as exemplified in Figure 2. This validation allowed us to assess both the accuracy of conformer generation and the plausibility of the TC models.

**Figure 2:**
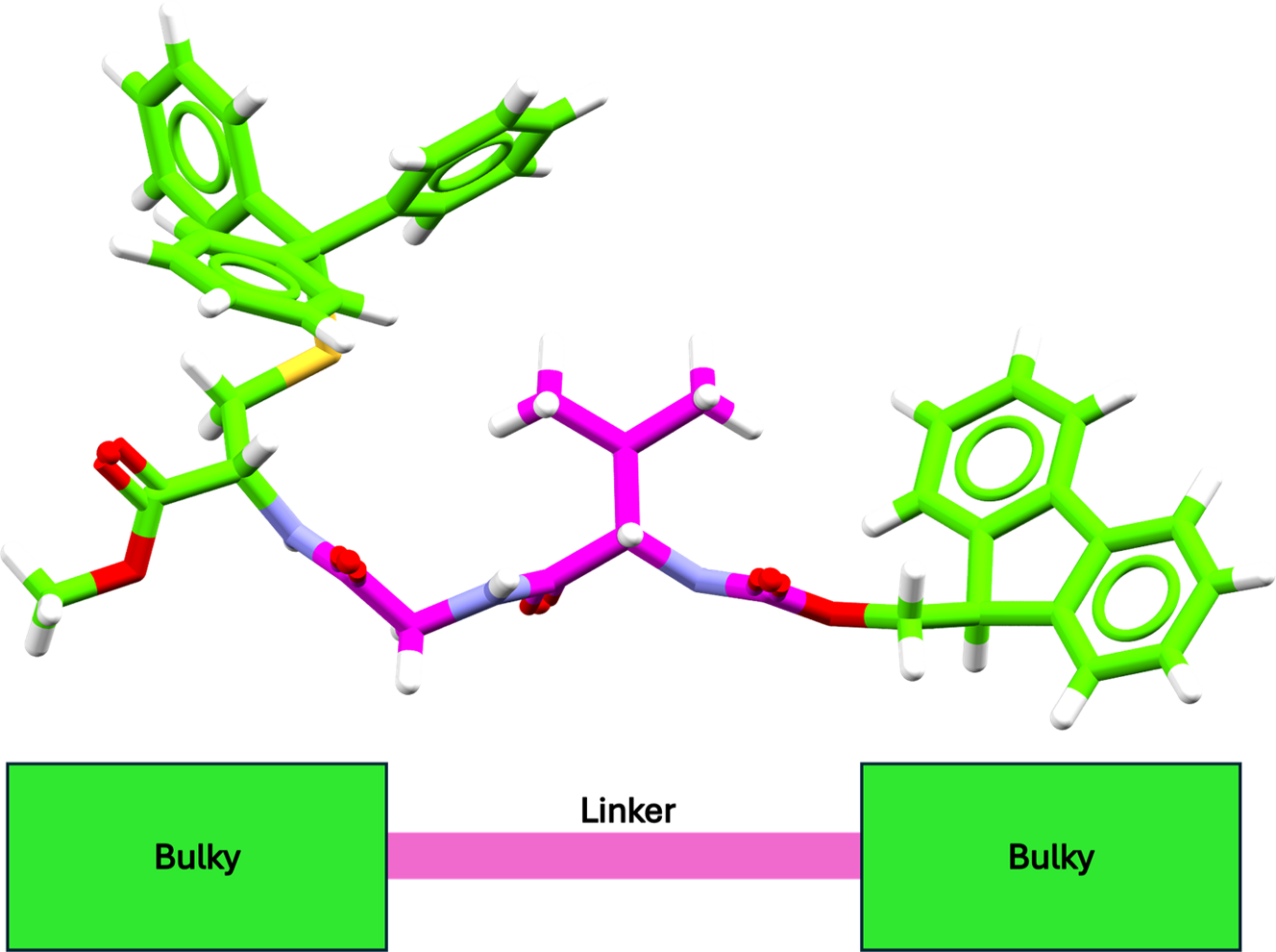
Schematic representation of our PROTAC-like definition and example of a PROTAC-like molecule retrieved from the CSD (Refcode HEDLOU). These molecules resemble PROTAC structures by having two bulky chemical moieties connected by a more flexible linker-like region.

To support downstream applications, we also implemented a clustering and selection strategy to reduce the ensemble size while preserving structural diversity.

### Terminology and Definitions

To ensure clarity in the following sections, we define several key terms used throughout this work. *PROTAC-like* molecules have already been defined as compounds resembling PROTACs in topology. A conformer *rank* refers to the order in which a conformer is generated, reflecting its likelihood based on its torsional preferences. *lig_RMSD* is the root-mean-square deviation (RMSD) between a generated PROTAC conformer and its experimentally observed structure, considering only the PROTAC ligand atoms. In contrast, *pp_RMSD* quantifies the deviation between the modeled and experimental protein–protein complexes, considering only protein C_α_ atoms. The *clash-score* quantifies steric overlaps between protein atoms, while the *surface-score* estimates the extent of protein–protein contact mediated by the PROTAC. We used the two scores to define a *plausibility window*, loosely denoting the range of score values typically associated with sterically and geometrically plausible TC models. Finally, the *compromise-score* is a weighted metric combining clash and surface-scores to guide the selection of representative TC models within this window. These metrics and concepts are described in detail in the Materials and Methods section.

## Materials and Methods

### PCG Workflow and Required Inputs

The PCG is implemented as a script using the CSD Python API,^51^ building upon the CSD Conformer Generator.^52^ Its key steps are summarized in Figure 3.

**Figure 3:**
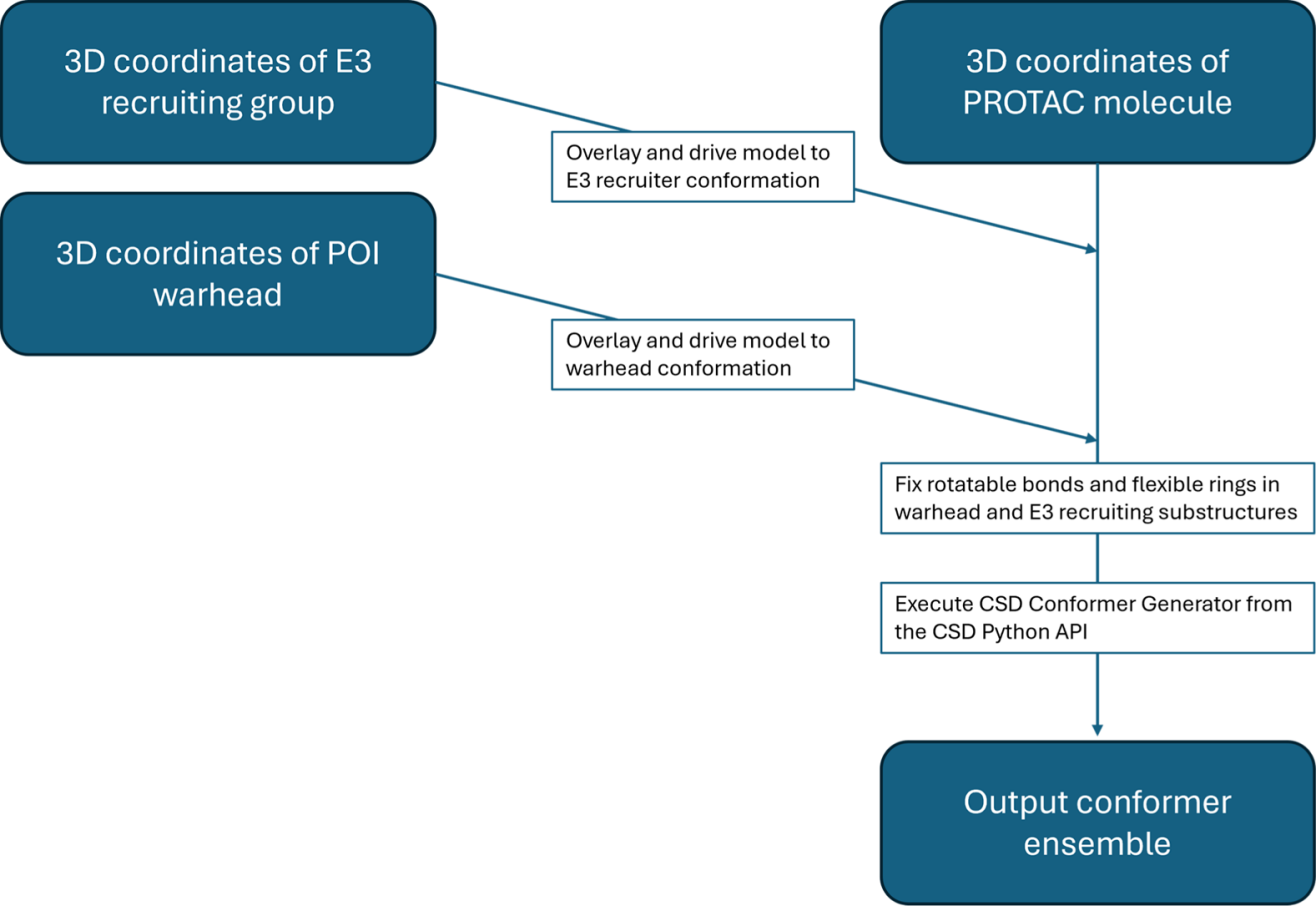
Schematic overview of the PCG workflow. The required inputs include a 3D model of the full PROTAC molecule and the protein-bound conformations of its warhead and E3 recruiting group. Optionally, the structures of the POI and E3 protein–ligand complexes can be provided to generate TC models.

The minimal input required by the PCG consists of initial 3D coordinates of: (1) the full PROTAC molecule model, (2) the E3 recruiting group protein-bound conformation, and (3) the POI warhead protein-bound conformation. If the 3D structure of the PROTAC molecule is unknown, it is possible to generate one from an SMILES representation using the CSD Python API. The protein-bound conformations are typically sourced from their monomer protein–ligand complex crystal structures, but they could also be obtained with molecular docking.

Before conformer generation, the acyclic torsion angles of the warhead and E3 recruiting group within the full PROTAC are adjusted to match those of their monomer-bound conformations. These substructures are then internally frozen but remain free to move as rigid bodies relative to the linker during conformer generation.

The PCG invokes the CSD Conformer Generator to sample the linker conformational space by using a systematic, knowledge-based approach. For each rotatable bond, representative torsion angles (typically 3–10) are selected from torsional distributions derived from experimental data in the CSD using kernel density estimation (KDE).

Conformations are generated via a depth-first search strategy, prioritizing torsions in the decreasing order of empirical probability. The search is pruned dynamically based on steric clashes, low-probability torsions, and conformer similarity. This ensures efficient and exhaustive sampling without relying on stochastic or Monte Carlo methods. The maximum number of conformers refers to the final set of unique, diverse conformers retained after clustering (rather than the total number of conformations explored during the search).

If the full structures of the monomeric POI and E3 protein–ligand complexes are provided, then the PCG is also capable of generating TC models. This is done by identifying the maximum common subgraph between the monomer ligands and their corresponding PROTAC substructures, aligning them, and applying the resulting transformation matrix to the associated proteins. As noted in the introduction, this rigid-body superposition was originally implemented primarily to compute geometric scores that assess steric clashes and protein–protein proximity. However, we observed that many of the resulting assemblies were structurally reasonable, which motivated further analysis of their quality and diversity.

Because the alignment does not account for protein flexibility or induced fit, some conformers may yield unrealistic TCs. For example, very compact conformations can lead to severe protein–protein clashes, while extended conformations may result in little or no contact between the proteins. To identify and discard such cases, the PCG computes two geometric scores: clash-score, defined as the number of heavy atom pairs closer than 2 Å, and surface-score, defined as the number of heavy atom pairs between 2 and 4 Å. To isolate the contribution of the PROTAC to PPIs, the corresponding values for the unbound proteins are subtracted from those of the TC. Low clash-scores indicate fewer steric clashes, while high surface-scores reflect greater interfacial contact. For consistency in this analysis, both scores were normalized separately within each ensemble to an arbitrary scale from 1 to 10, where 1 corresponds to the lowest value and 10 corresponds to the highest value observed in that specific ensemble.

The PCG supports parallel execution with a user-defined thread count. The maximum number of conformers can also be specified, although it may not be reached if the conformational space is exhausted earlier. For benchmarking or validation, a reference molecular structure can be provided to compute the RMSD for each generated conformer (lig_RMSD).

### Selection of PROTAC Structures from the PDB

The primary validation dataset consists of 34 PROTAC structures available in the PDB^35^ at the time of the study. These are reported in Table 1 and were retrieved by searching for relevant terms (e.g., PROTAC, LYTAC, AUTAC, and Chimera), followed by a visual inspection to confirm the presence of a chimeric degrader.

**Table 1:** Primary dataset for PCG validation^a^

PROTAC	E3	POI	linker-containing SMARTS substructure
5T35 ^2^	4W9H	3MXF	CC(=O)[N:1]CCOCCOCC[O:2]CC(=O)N
6BN7 ^41^	4TZ4	3MXF	[O:1]CC(=O)NCCCCCCCCN[C:2]=O
6BOY^41^	4TZ4	3MXF	[N:1]CCCCCCCCNC(C[O:2])=O
6HAX^56^	5NVX	6HAZ	[C:1]c1ccc(CC[O:2])cc1
6HAY^56^	5NVX	6HAZ	[O:1]CCOCCOC[C:2]N
6HR2 ^56^	5NVX	6HAZ	[C:1]c1ccc(CC[O:2])cc1
6W7O^58^	4MTI	5P9J	[O:1]Cc1cnc(C[O:2]C=O)cn1
6W8I^58^	4MTI	5P9J	[O:1]CCOCCOCCOCCOCC[O:2]C=O
6ZHC^59^	4W9H	4QVX	[O:1]CCOCCOCCOCCOCCOC[C:2]C=O
7JTO^60^	4W9H	4QL1	[C:1]CNC(=O)CCCCCCCCCC(=O)[N:2]CC = O
7JTP^60^	4W9H	4QL1	N[C:1](CN[C:2]=O)=O
7KHH^61^	4W9H	7KHL	NC(=O)C[C:1]CCCCCCCC[N:2]C=O
7PI4 ^62^	4W9H	4C7T	NC(=O)C[N:1]1CC[N:2]CC1
7Q2J^63^	4W9H	4QL1	O=[C:1]NCC[C:2]CC(=O)N
7S4E^64^	5NVX	6HAZ	C(COc1ccc([C:1]N2CCNCC2)cc1)[O:2]c3ccccc3
7TVA^65^ ^,^ ^b^	4TZC	7TVB	CN([C:1]CCC#[C:2]c1cccc2C(=O)NCc12)C(=O)CCNc3ccc(cc3)
7Z6L^66^	5NVX	7Z78	[O:1]CC[C:2]N1CCCCC1
7Z76 ^66^	5NVX	7Z78	C(=O)N[C:1]COC(C)[C:2]N1CCCCC1
7Z77 ^66^	5NVX	7Z78	O=CNC[C:1]C[C:2]N1CCCCC1
7ZNT^67^	5NVX	3MXF	O=C[N:1]CCCCCC[S:2]C(C)(CC=O)C
8BB2 ^68^	4W9H	4QL1	NC(=O)C[C:1]OCCOCCOCCOCCN[C:2]=O
8BB3 ^68^	4W9H	4QL1	NC(=O)C[C:1]OCCOCCOCCOCCN[C:2]=O
8BB4 ^63^	4W9H	4QL1	NC(=O)C[C:1]CN[C:2]=O
8BB5 ^63^	4W9H	4QL1	NC(=O)C[c:1]1ccc(CN[C:2]=O)cc1
8BDS^69^	8BDJ	7RN2	O=CNC[C:1]C(=O)NCCOCCOCCOCC[N:2]C(=O)C
8BDT^69^	8BDJ	7RN2	O=CNC[C:1]C(=O)NCCOCCOCCOCC[N:2]C(=O)C
8BDX^69^	8BDJ	7RN2	O=CNC[C:1]C(=O)NCCOCCOCCOCC[N:2]C(=O)C
8BEB^69^	8BDL	7RN2	CC(=O)N[C:1]CCCN[C:2](=O)CCc1ccc(cc1)
8DSO^70^ ^,^ ^b^	4HY4	5P9J	[C:1]N1CCN(CCOCC(=O)[N:2])CC1
8EXC^71^ ^,^ ^b^	8EMU	5AMI	O=CNCC[C:1]OCCCCOCCC[N:2]c1cccc2C(=O)NC(=O)c12
8EXG^71^ ^,^ ^b^	8EMU	5AMI	O=CNCCOC[C:1]OCC[N:2]c1cccc2C(=O)NC(=O)c12
8EYL^71^ ^,^ ^b^	8EMU	6R0Q	OC(=O)c1ccccc1[N:1]C[C:2]OCCNC=O
8G1P^49^	5NVX	6HAZ	c1cccc([O:1]CCc2ccc([C:2]N3CCNCC3)cc2)c1
8G1Q^49^	5NVX	6HAZ	CC(C)(C)CN[C:1](=O)c1ccn[c:2](c1)N2CCNCC2

a Each row identifies a different PROTAC case and contains (1) the PDB code of the PROTAC complex, (2) the PDB code of the E3 complex, (3) the PDB code of the POI complex, and (4) a SMARTS^57^ code defining a linker-containing substructure. These codes were manually curated to ensure a single substructure match in the PROTAC. The linker substructure, defined as the region absent from both the warhead and the E3 recruiting group (as observed in their monomer protein complexes), is enclosed between the atoms labeled [:1] and [:2] using an extension to the SMARTS pattern syntax.

b TC assembly not performed for these systems.

Most of the structures are TCs and were used to test both the PCG’s conformational ensemble generation and the quality of TC modeling. A few cases (7TVA, 8EXC, 8EXG, and 8EYL) involve PROTACs in complex with a single protein and were therefore used only for conformer validation. One additional case (8DSO) involves a covalently bound PROTAC, which is not currently supported by our workflow and was excluded from TC modeling.

The dataset is relatively skewed in terms of E3 representation. Most complexes involve VHL E3, with only a few cases using CRBN (6BN7 and 6BOY) or cellular inhibitor of apoptosis protein (cIAP; 6W7O and 6W8I). In contrast, the POI side shows broader diversity, including BRD4 (5T35, 6BN7, 6BOY, 7KHH, 7ZNT, 8BDS, 8BDT, 8BDX, and 8BEB), SMARCA2/4 (6HAX, 6HAY, 6HR2, 7S4E, 7Z6L, 7Z76, 7Z77, 8G1P, and 8G1Q), WDR5 (7JTO, 7JTP, 7Q2J, 8BB2, 8BB3, 8BB4, and 8BB5), BTK (6W7O and 6W8I), BCL-XL (6ZHC), and FAK (7PI4). Notably, several PROTACs recur across multiple entries, either in complex with different POIs or even within the same TC but adopting distinct conformations such as 8BB2 vs 8BB3 or 6HAX vs 8G1P. Each of these entries was treated independently during validation to capture potential conformational variability.

### Extraction of PROTAC-like Molecules from the CSD

To further validate the PCG, a secondary validation set of PROTAC-like molecules was assembled by mining the CSD. These PROTAC-like molecules were defined as compounds featuring two bulky terminal groups connected by a flexible linker region, mimicking the overall topology of the PROTACs but without requiring functional activity or protein binding.

A database of SMILES strings was constructed by combining all available PROTACs from PROTAC-DB (3270 entries at the time of the study) with a filtered subset of the CSD containing nonpolymeric organic molecules with at least seven atoms and fully determined atomic coordinates (780204 entries at the time of the study). For each entry, 124 two-dimensional molecular descriptors were calculated using the RDKit cheminformatics toolkit (version 2023.09.6).^72^ Redundant and highly correlated descriptors were removed using the procedure described by Comesana et al. (2022)^73^ to mitigate multicollinearity. A detailed description of the procedure and the final set of 62 selected features is provided in the Supporting Information.

Principal component analysis (PCA) was then performed using scikit-learn (version 1.3.0)^74^ to reduce dimensionality and identify CSD molecules with structural characteristics like those in PROTAC-DB. A total of 34 principal components (PCs) were retained, accounting for 90% of the total variance. To ensure robust similarity across multiple dimensions of chemical space, only CSD entries falling within the same range as PROTAC-DB entries across all PCs were retained (Figure S35). After additional automatic filtering (Figure S36) and manual inspection, 116 CSD molecules were selected as the final PROTAC-like validation set (the full list is provided in the Supporting Information).

### PCG Validation Workflow and Input Preparation

An auxiliary script based on the CSD Python API was used to automate the preparation of input files and the batch execution of the PCG across all PROTACs in the primary validation set. For each case, the PCG was run using the full PROTAC molecule and the protein-bound conformations of the warhead and E3 recruiting group, extracted from separate monomeric protein–ligand complexes (second and third column of Table 1). These monomer complexes were selected to reflect a typical modeling scenario in which the TC structure is not available. In cases where exact matches were not found, homologous structures were used (e.g., SMARCA2 vs SMARCA4 or BRD4-BD1 vs BRD4-BD2).

The script accepted a CSV file as input, structured according to Table 1, and performed the following steps: (1) download of the relevant PDB entries; (2) extraction of the PROTAC, E3, and POI ligands from the downloaded structures; (3) preparation of the ligands as input files (bond-type recognition and addition of missing hydrogens); (4) protonation and cleaning of protein–ligand complexes with removal of additives, metals, water molecules, and nonessential chains (only protein chains in close proximity to the PROTAC ligand were retained; for example, elongin B and C subunits were removed from VHL complexes); and (5) identification of the E3 recruiting group and warhead substructures in the PROTAC using a SMARTS-based fragmentation procedure. The linker-containing SMARTS codes from Table 1 were used to remove the linker from the PROTAC and match the remaining fragments to the respective monomer ligands with maximum common subgraph (details in the Supporting Information); (6) saving of all prepared input files in mol2 format; and (7) execution of the PCG with a maximum of 5000 conformers per case.

The crystal structure of the PROTAC ligand was used as a reference to compute the lig_RMSD for each generated conformer. MMFF94 energies were calculated using KNIME Analytics Platform (version 5.2.1)^75^ with ChemAxon/Infocom JChem Extensions^76^ and Vernalis^77^ nodes. Intramolecular geometry assessment with Mogul,^78,79^ the CSD knowledge-based tool for validation of intramolecular geometry was used to highlight unusual features in selected cases.

For the secondary validation set, some manual preparation was required. The 3D structure of each PROTAC-like molecule was retrieved from the CSD; head and tail substructures were selected by visual inspection and manually extracted into mol2 files (available in the GitHub repository). The PCG was then executed in batch mode for each case with a maximum of 5000 conformers. The original crystal structure of each entry was used as the reference for computing lig_RMSD.

### Evaluation of Ternary Complex Model Quality

To evaluate the quality of the TC models constructed through rigid-body assembly, we introduced pp_RMSD. This metric quantifies the deviation in the relative orientation of the POI and E3 proteins with respect to the experimental reference.

Unlike other modeling approaches that extract the POI and E3 components directly from the TC crystal structure, our workflow uses separate monomeric protein–ligand complexes as input, making the calculation of RMSD values less straightforward. Each TC model was first superimposed onto its reference by aligning only the E3 chains using the protein superposition functionality of the CSD Python API. To ensure that RMSD calculations were based on structurally equivalent atoms, we generated Cα-only representations of the protein backbones. Pairwise sequence alignments in PyMOL were used to identify a common set of structurally conserved residues between each model and its reference. These Cα-only structures excluded all nonprotein atoms and any residues not present in the common set. Finally, using these prealigned, Cα-only structures, a single RMSD value (pp_RMSD) was computed for the full protein complex (excluding the PROTAC ligand) using the CSD Python API. As the alignment was performed on the E3, the resulting pp_RMSD value provides a focused measure of the deviation in the POI’s orientation within the predicted model relative to the experimental structure.

This analysis was performed for the top 100 conformers (or fewer, if not available) with the lowest lig_RMSD values and with a clash score of ≤1.1 and a surface score of ≤1.2 for each PROTAC case.

### Clustering and Ensemble Reduction Strategy

To reduce the size of the conformational ensemble while preserving structural diversity, we implemented a clustering and selection procedure, applied to 6HAY as a case study. The E3 chains for all the models with clash-score ≤1.1 and surface-score ≤1.2 were aligned to the corresponding chain of the lowest clash-score model using a structure alignment approach. Subsequently, the inertia tensors of all the structures’ POIs were calculated with Python library MDAnalysis (version 2.7.0.).^80,81^ The eigenvectors, representing the principal axes of inertia (Figure S102), were extracted, normalized, and concatenated into a 9 dimensional vector for each object. This representation, capturing the spatial orientation of the principal axes, enabled clustering based on rotational configurations. The elbow-method and silhouette scores were employed to select an optimal k value of 35 to proceed with k-means clustering using the Python scikit-learn library (Figures S103–S106).

To identify representative models within each cluster, a compromise-score was calculated based on the clash- and surface-scores. Both scores were first normalized to a [0, 1] range, ensuring comparability. KDE was performed on the distributions of the normalized clash- and surface-scores from the experimental structures to capture their probability densities (Figure S107). The compromise-score was defined as *S*_compromise_ = 1 – (*w*_clash_ *S*_clash_ + *w*_surface_(1 – *S*_surface_)) where *S*_compromise_ reflects the desirability of a model (values closer to 1 indicate better agreement with experimental reference distributions), *w*_clash_ and *w*_surface_ are the relative weights of the normalized clash- and surface-scores (*S*_clash_ and *S*_surface_, respectively). To determine the optimal weights, an optimization procedure using Python SciPy version 1.8.1 ^82^ optimize.minimize method was employed, maximizing the similarity of selected models to the KDE reference distributions. Regularization in the form of α∑_*i*_ *w*_i_^2^ (where α = 0.1 and *w*_*i*_ is the individual weights) was incorporated to ensure balanced weighting and prevent over-emphasis on a single parameter. Once the optimal weights were determined (*w*_clash_ = 0.62 and *w*_surface_ = 0.38 for the example case 6HAY), the compromise-score was applied to all models, and the highest-scoring model from each cluster was selected to represent the final ensemble.

## Results and Discussion

### Validation of Conformer Generation

The first step in this study was to assess whether PCG can reproduce experimentally observed PROTAC molecular conformations. As discussed in the Introduction section, X-ray structures remain the most detailed reference available for PROTAC validation despite some limitations. We therefore applied the validation protocol described in Materials and Methods to the primary validation set of PROTAC structures from the PDB (Table 1) and the secondary set consisting of PROTAC-like molecules from the CSD. Figure 4 shows the lig_RMSD of the closest conformer for each of the 150 molecular conformational ensembles generated for both sets, plotted against its generation rank. Although the PROTAC-like molecules are not technically ligands, we refer to their RMSD as lig_RMSD for the sake of consistency.

**Figure 4:**
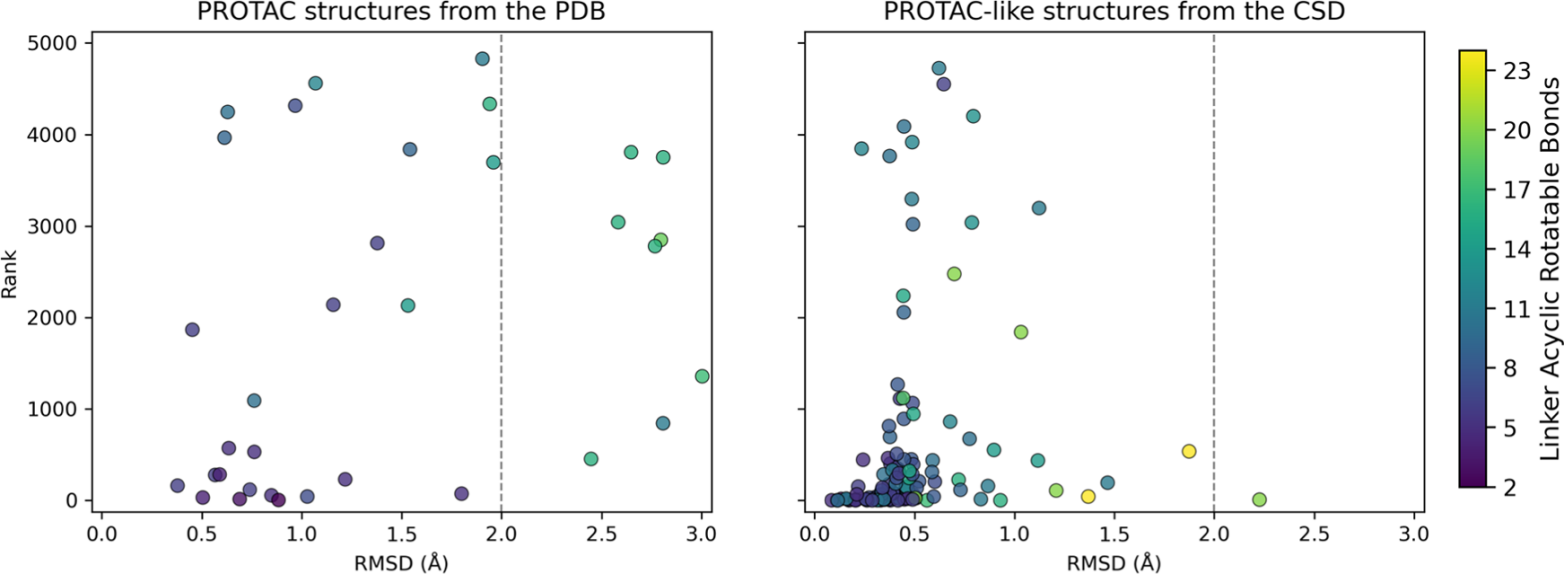
Scatterplots of rank vs lig_RMSD for the closest conformers to the experimental PROTAC crystal structures from the PDB (left) and PROTAC-like structures from the CSD (right). Each point is colored according to the number of acyclic rotatable bonds in the linker, as shown in the color scale.

The PCG performed particularly well on the secondary set (PROTAC-like molecules). In 75% of the cases, the closest conformers had a lig_RMSD below 0.5 Å, in 93% below 1 Å, and in 99% below 2 Å. Moreover, 17% of them were ranked within the top 10, 47% within the top 100 and 84% within the top 1000 structures. These results indicate that, for this set, the PCG not only reproduces experimental structures accurately but also performs acceptably at prioritizing them within the ensemble.

The performance on the primary set was also satisfactory, although it was slightly less favorable than for the secondary set. Among the closest conformers, 6% had a lig_RMSD below 0.5 Å, 46% below 1 Å, and 76% below 2 Å. Ranking was also more challenging in this context: only 3% of the closest conformers appeared in the top 10, 18% in the top 100, and 44% in the top 1000 structures. Nonetheless, the fact that three-quarters of the closest conformers fall below 2 Å lig_RMSD confirms the PCG’s ability to approximate PROTACs conformations in their protein–ligand complex crystal structure. Notably, several cases showed excellent agreement (lig_RMSD ≤1.0 Å), including 6HAX, 6HAY, 6HR2, 7JTP, 7PI4, 7Q2J, 7S4E, 7Z76, 7Z77, 8BB5, 8BEB, 8EXC, 8EYL, 8G1P, and 8G1Q.

Given that the two validation sets pose comparable challenges, as indicated by the number of acyclic rotatable bonds in the linker (Figures 4 and S37), the observed performance gap likely stems from differences in the environments (protein vs molecular crystal) and the quality of the reference structures. Since the conformer ranking is based solely on internal geometry and does not account for the protein context of the reference structures, it is not surprising that many of the closest conformers in the primary set are poorly ranked, as shown in Table 2. Nevertheless, other lower-rank conformers for those cases are still close to the reference structures. Reducing the conformational sampling to 1000 conformers resulted in only a marginal increase in mean lig_RMSD across the primary validation set closest conformers, from 1.46 Å (with 5000 conformers) to 1.61 Å. Only 2 cases showed an increase greater than 0.5 Å (Table S2 and Figures S39 and S40). This suggests that depending on the application, a narrower conformational search may be sufficient. However, given the relatively low computational cost of a broader sampling, users may still prefer to generate larger ensembles. On average, the PCG required only 6 min per PROTAC to generate 5000 conformers on a workstation with 24 GB RAM and 16 Intel­(R) Xeon­(R) Gold 5218R physical cores (32 threads with hyper-threading).

**Table 2:** Summary of PCG results^a^

PROTAC	rank	lig_RMSD (Å)	Δ*E* (kcal/mol)	clash-score	surface-score
5T35	4828	1.90	–22	1.46	1.52
6BN7	3695	1.96	–61	2.91	2.93
6BOY	2132	1.53	–11	1.02	1.02
6HAX	279	0.57	6	1.00	1.08
6HAY	4246	0.63	–17	1.04	1.21
6HR2	162	0.38	7	1.13	1.27
6W7O	2814	1.38	–7	1.00	1.05
6W8I	1358	3.00	–26	3.10	3.26
6ZHC	2849	2.80	–4	1.16	1.20
7JTO	4334	1.94	–27	1.07	1.05
7JTP	56	0.85	58	1.82	1.69
7KHH	844	2.81	0	1.00	1.01
7PI4	5	0.88	–1	2.02	1.70
7Q2J	1867	0.45	5	1.02	1.04
7S4E	4315	0.97	6	1.03	1.11
7TVA^b^	74	1.80	6		
7Z6L	572	0.64	–8	1.18	1.24
7Z76	1092	0.76	9	1.00	1.07
7Z77	32	0.51	–1	1.00	1.05
7ZNT	3965	0.61	8	1.02	1.04
8BB2	3043	2.58	–18	1.01	1.04
8BB3	454	2.45	–2	1.14	1.22
8BB4	231	1.22	18	1.00	1.03
8BB5	531	0.76	2	1.01	1.06
8BDS	3750	2.81	0	3.87	3.96
8BDT	3806	2.65	–69	1.10	1.15
8BDX	2781	2.77	–20	2.02	2.08
8BEB	43	1.03	–8	1.00	1.01
8DSO^b^	3837	1.54	–21		
8EXC^b^	4560	1.07	–5		
8EXG^b^	2140	1.16	3		
8EYL^b^	284	0.59	–1		
8G1P	118	0.74	2	1.24	1.36
8G1Q	15	0.70	–3	1.14	1.25

a For each PROTAC, the table reports the rank and lig_RMSD of the closest conformer, the MMFF94 energy difference relative to the experimental structure (*E*_conf_ – *E*_exp_), and the clash- and surface-scores of the corresponding TC model.

b TC assembly not performed for these systems.

The efficiency of the PCG stems from its knowledge-based nature. Unlike energetics-based methods that rely on FFs to identify low-energy molecular geometries, our approach leverages experimental data from the CSD to generate conformers with realistic geometric parameters. Although not explicitly optimized for energy, PCG still produces low-energy conformations. Table 2 reports the MMFF94 energy differences between the experimentally observed PROTAC ligands and their closest PCG-generated conformers. Consistently with their geometric similarity, we observe a strong linear correlation between their energies (*R* ^2^ = 0.97; Figure S41). Notably, even conformers with higher lig_RMSD often exhibit comparable energies to their experimental counterparts, further supporting the PCG’s ability to reproduce conformations observed in protein–ligand complex crystal structures, despite not being trained on protein-bound data.

The ligand RN3 in PDB structure 6BN7 represents the most notable outlier, with the closest PCG-generated conformer exhibiting a 2-fold lower energy than its experimental reference (61 vs 122 kcal/mol; Table S3). Inspection of the crystal structure revealed missing electron density in the linker region, prompting a Mogul^78,79^ geometry check that revealed several highly unusual torsion angles (Figure S42). By contrast, the PCG-generated conformer has no unusual features, consistent with its lower energy.

Two additional outliers are 8BDT and 7JTP (Figure S43). In the case of 8BDT, the closest conformer appears to be energetically more favorable than the crystallographic structure, likely due to a more extended geometry increasing the distance between the warhead and the E3 binding group, consistent with its relatively high lig_RMSD value of 2.65 Å. For 7JTP, the closest conformer is geometrically similar to the experimental structure (lig_RMSD = 0.85 Å), but the energy differs significantly. This discrepancy appears to stem from the torsion angle around the bond connecting the two amide groups in the linker. The PCG favored a more open conformation with the amide nitrogens in an *anti* arrangement. A search of the CSD indicates that both *anti* and *syn* conformations are equally accessible in such systems. In the 7JTP crystal structure, the conformation appears to be facilitated by water mediation between the linker amide carbonyls and the two monomer proteins.

In summary, the results presented in Figure 4 and Table 2 demonstrate that the PCG is capable of generating conformations that are both geometrically and energetically consistent with the experimentally observed PROTAC structures. This confirms its utility in producing conformers that approximate protein-bound states. This is particularly relevant for cases where we lack structural information for the PROTAC ligand itself, but we have information on the POI and E3 ligand complexes. The PCG can be used to sample the linker conformational space and derive a low-energy conformer ensemble, resembling potential bound-state PROTAC conformations.

### Scoring-Based Filtering Enabled by Ternary Complex Assemblies

As shown in Figure 5, some TC models generated by rigid-body alignment can closely resemble experimental structures. Others, however, may suffer from severe steric clashes or lack meaningful PPIs. These issues typically arise from overly compact or extended conformers, respectively.

**Figure 5:**
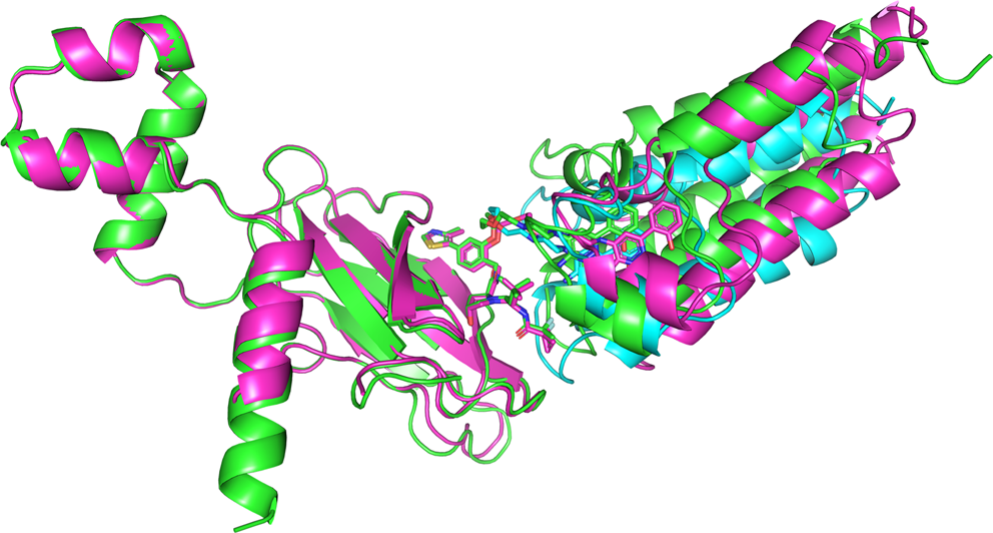
Structural overlay of the 6HAY PROTAC-mediated TC crystal structure (green) and two PCG-generated models: one based on the closest conformer (cyan, lowest lig_RMSD) and one with the best POI-E3 orientation (magenta, lowest pp_RMSD). The structures were aligned on the E3 using PyMOL (Cα atoms; ligand excluded).^83^

To identify and discard such cases, we employed the clash-score and surface-score defined in the Materials and Methods section. Both scores were normalized within each ensemble and used to define a “plausibility window” for model selection. Their values for the models based on the closest conformers are reported in Table 2. Figure 6 illustrates the distribution of clash and surface scores for the full ensemble of 6HAY (similar plots for all PROTAC cases are provided in Figures S44–S72). As expected, the two scores are heavily correlated, but the spread of surface-score values in the low clash-score region is particularly informative. Focusing on the low clash-score values allows us to filter out models with steric clashes, while higher values of the surface-score for that specific region should highlight models with a higher degree of PPIs.

**Figure 6:**
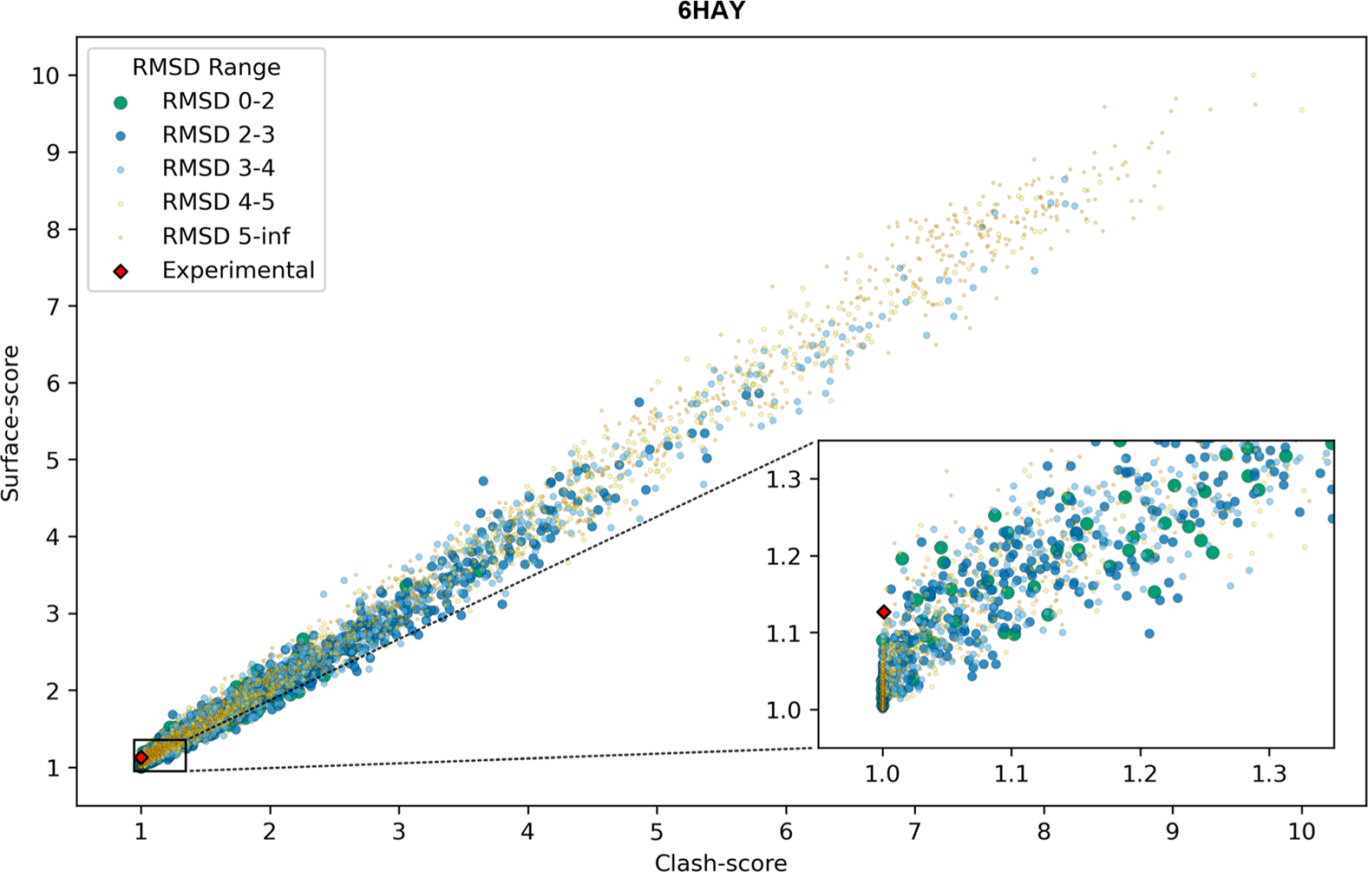
Surface-score vs clash-score scatterplot for the full conformational ensemble of 6HAY. Each point represents a model generated by the PCG. Point color, size, and transparency reflect a scale of lig_RMSD (Å). The red diamond marks the scores of the experimental TC structure for reference. This plot highlights the distribution of models across the steric plausibility space and illustrates how the low-clash, high-surface-score window in the inset is enriched in conformers closer to the experimental geometry.

To confirm this hypothesis, we calculated the clash and surface scores for all experimentally determined TC structures in the primary set. Figure 7A–D shows the combined distributions of these scores.

**Figure 7:**
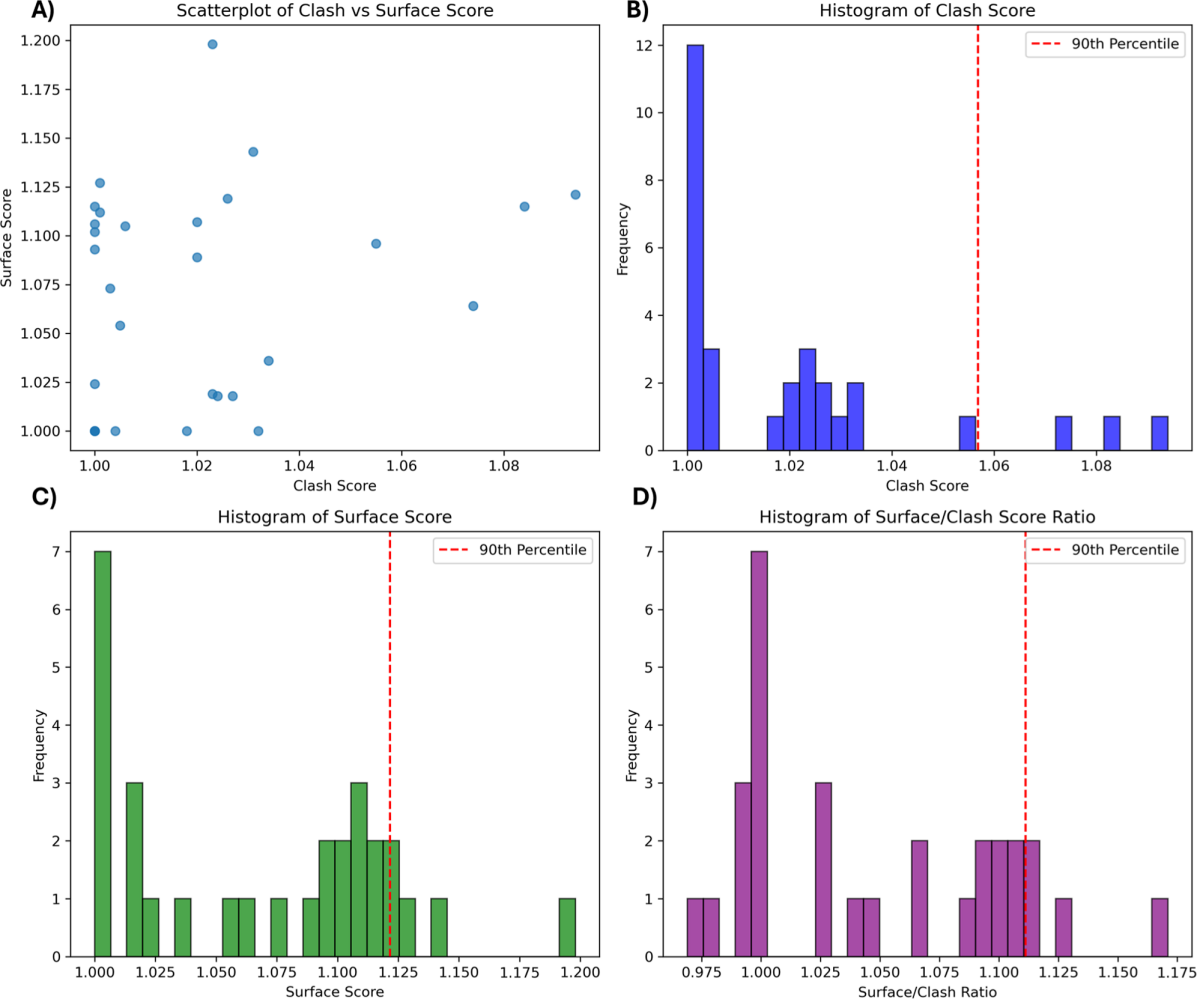
Distribution of clash-score and surface-score for the experimental TC structures. (A) Surface-score vs clash-score scatterplot. (B) Histogram of clash-score. (C) Histogram of surface-score. (D) Histogram of surface-to-clash ratio.

All experimental TCs fall within a narrow range: clash-score between 1.0 and 1.1 and surface-score between 1.0 and 1.2. In most cases, the surface-to-clash ratio exceeds 1, in line with our original hypothesis. However, a few exceptions show ratios close to or below 1, indicating that selecting models solely based on surface-to-clash ratio may not always be optimal. This is further illustrated in Figures S44–S72, which report surface-score vs clash-score scatterplots for all PROTACs, including the scores of their corresponding experimental references. While the experimental surface-scores typically lie in the upper range of values observed for their clash-score, some cases deviate from this trend. These observations help define a plausibility window, guiding the selection of conformers that are most likely to yield viable TC assemblies.

Comparing the clash- and surface-scores of PCG-generated models built upon the closest conformers (Table 2) to the experimental reference range reveals that many fall within the plausibility window but some exhibit significantly higher values. This underscores the fact that lowest lig_RMSD conformers do not necessarily yield the best TC models, especially when the closest conformers do not align perfectly to their experimental reference.

To illustrate this, we examine the 8BDS case depicted in Figure 8. The closest conformer (lig_RMSD = 2.81 Å) yields a model with quite compact geometry, severe steric clashes (clash score = 3.9), and poor agreement with the experimental TC. In contrast, the model with the lowest pp_RMSD is clash-free and closely reproduces the POI-E3 orientation.

**Figure 8:**
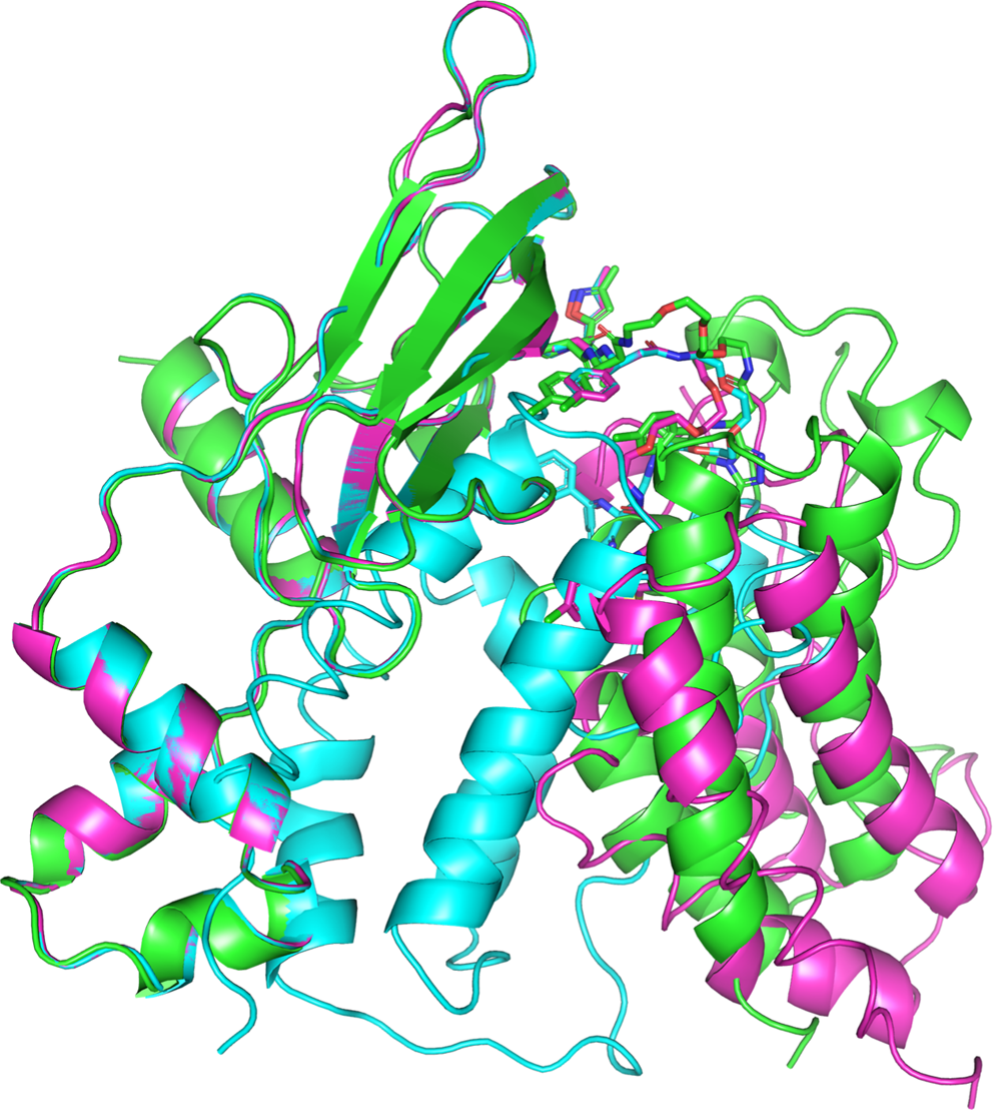
Structural overlay of the 8BDS PROTAC-mediated TC crystal structure (green) and two PCG-generated models: one based on the closest conformer (cyan) and one with the lowest pp_RMSD (magenta). The closest conformer model shows severe steric clashes, while the lowest pp_RMSD model is clash-free and closely reproduces the experimental POI-E3 orientation. The structures were aligned on the E3 using PyMOL (Cα atoms; ligand excluded).^83^

### Evaluation of Ternary Complex Models

As discussed above, the closest conformer does not always yield the most accurate TC model. To properly assess the quality of the generated assemblies, we first identified plausible models within each ensemble, as detailed in the Materials and Methods section. We computed pp_RMSD for the 100 lowest lig_RMSD conformers within the plausibility window for each case, which allowed us to identify alternative and more accurate TC models across the dataset.

In several cases (5T35, 6W8I, 6ZHC, 7JTP, 7PI4, 8BDX, and 8G1P), the closest conformer led to models with significant steric clashes (Table 2), but pp_RMSD enabled the selection of clash-free models with an improved POI-E3 orientation elsewhere in the ensemble. For other systems such as 8BB2, 8BB3, 8BB4, and 8BEB, the closest conformer models were already clash-free, yet alternative conformers yielded better agreement with the experimental POI-E3 geometry. In most remaining cases, the closest conformer produced a TC model that was both sterically plausible and structurally consistent with the crystal structure. Slightly better models were found in all but two cases, 6HAX and 7Z76, where the closest conformer also yielded the model with the lowest pp_RMSD (Figures S73–S101).

The only case in which the PCG failed to generate a clash-free TC model was that of 6BN7, highlighting a key limitation of our current approach. All generated models placed the linker in steric conflict with a loop of the CRBN protein (Figure S74). This happens because the proteins are rigidly superimposed without considering the position of the linker. The unusual linker conformation seen in the experimental structure, which avoids the loop, is not sampled by the PCG because it falls outside the typical torsional preferences. As a result, no model of acceptable quality could be generated in this case. Interestingly, the pp_RMSD values for several models were relatively low, which would have suggested a good match if they were considered alone. However, the clash-score clearly revealed that these models were not physically plausible.

All of the best pp_RMSD values for each PROTAC, along with their rank and sampling statistics, can be found in Table 3.

**Table 3:** Lowest pp_RMSD (Å) observed for each PROTAC, along with the rank of the corresponding conformer, the number of models with pp_RMSD <10 Å (*n* < 10 Å), and the number of sampled conformers (*n*_sampled)

PROTAC	pp_RMSD (Å)	rank	*n* < 10 Å	*n*_sampled
5T35	3.36	2836	52	100
6BN7	6.71	4528	6	21
6BOY	10.97	2131	0	100
6HAX	2.57	279	75	100
6HAY	3.60	624	53	100
6HR2	3.04	120	51	100
6W7O	4.26	3021	17	100
6W8I	13.96	151	0	100
7JTO	7.72	4566	2	100
7JTP	5.16	68	3	17
7PI4	8.84	46	1	8
7Q2J	2.22	1415	39	100
8BB2	7.03	4422	1	100
8BB3	9.81	2513	2	100
6ZHC	6.10	2827	2	100
8BB4	9.57	313	2	100
8BB5	3.22	191	32	100
7KHH	9.41	1083	2	100
7S4E	3.20	4267	35	100
7Z6L	2.46	579	51	100
7Z76	1.41	1092	72	100
7Z77	4.26	34	11	98
7ZNT	2.75	2582	67	100
8BDS	5.07	4899	37	100
8BDT	3.85	4483	77	100
8BDX	4.25	3164	44	100
8BEB	6.89	2113	5	100
8G1P	2.15	292	62	100
8G1Q	4.21	35	3	6

Drummond et al.^38^ and later Zaidman et al.^45^ adopted an RMSD threshold of 10 Å to define crystal-like models. While there are slight differences in their RMSD definition and our pp_RMSD, we decided to adopt the same threshold to have a useful reference point for evaluating model quality.

It is important to note that this analysis was performed on a subset of the ensemble, specifically the 100 conformers with the lowest lig_RMSD within the plausibility window. As such, the statistics reported in Table 3 do not reflect the full conformational space explored by the PCG, and the actual number of crystal-like models may be higher. Nevertheless, the results provide a practical way to assess the presence and rank of plausible TC models across the dataset. Most PROTACs yielded at least one model with pp_RMSD below 10 Å, and in several cases, a substantial fraction of the sampled models fell within this range, suggesting that the method is generally effective at generating crystal-like TC geometries. Only two systems, 6BOY and 6W8I, did not yield any models below the threshold, but 6BN7 should also be considered a failure due to steric clashes in all generated models. This means that overall the PCG produced at least one crystal-like model in the sampled subset in 90% of cases.

The rank of the best model, however, varies considerably, often falling well outside of the top 1000. This confirms that while the method is capable of generating high-quality models, identifying them remains difficult. Improving model ranking remains a key area for future development, particularly through the integration of additional scoring functions.

### Clustering and Ensemble Reduction Strategy

Given the variability in model quality and the difficulty in identifying the best conformers based on ranking alone, we explored strategies to reduce the ensemble size while retaining structural diversity and plausibility. This is particularly important in real-case modeling scenarios where no experimental structure is available and users require a compact yet representative set of models for further analysis. To address this, we developed a clustering and selection workflow that focuses on the most promising region of the plausibility window while maximizing the coverage of POI-E3 orientations.

We applied this workflow, described in the Materials and Methods section, to 6HAY as a case study. Filtering out the models outside the plausibility window reduced the ensemble size from 5000 to approximately 1600. The remaining models yielded 35 clusters based on the POI’s principal axes of inertia after alignment on the E3. Each cluster groups a distinct rotational configuration of the POI-E3 orientation.

Figure 9A shows the resulting subset composed of one representative model per cluster. Figure 9B displays these representatives in a “hedgehog-like” spatial distribution, highlighting the range of POI orientations relative to the fixed E3. The representative models were selected using the previously described compromise score, which proved more effective than using the surface-to-clash ratio in matching the KDE distribution of experimental structures (Figures S108 and S109).

**Figure 9:**
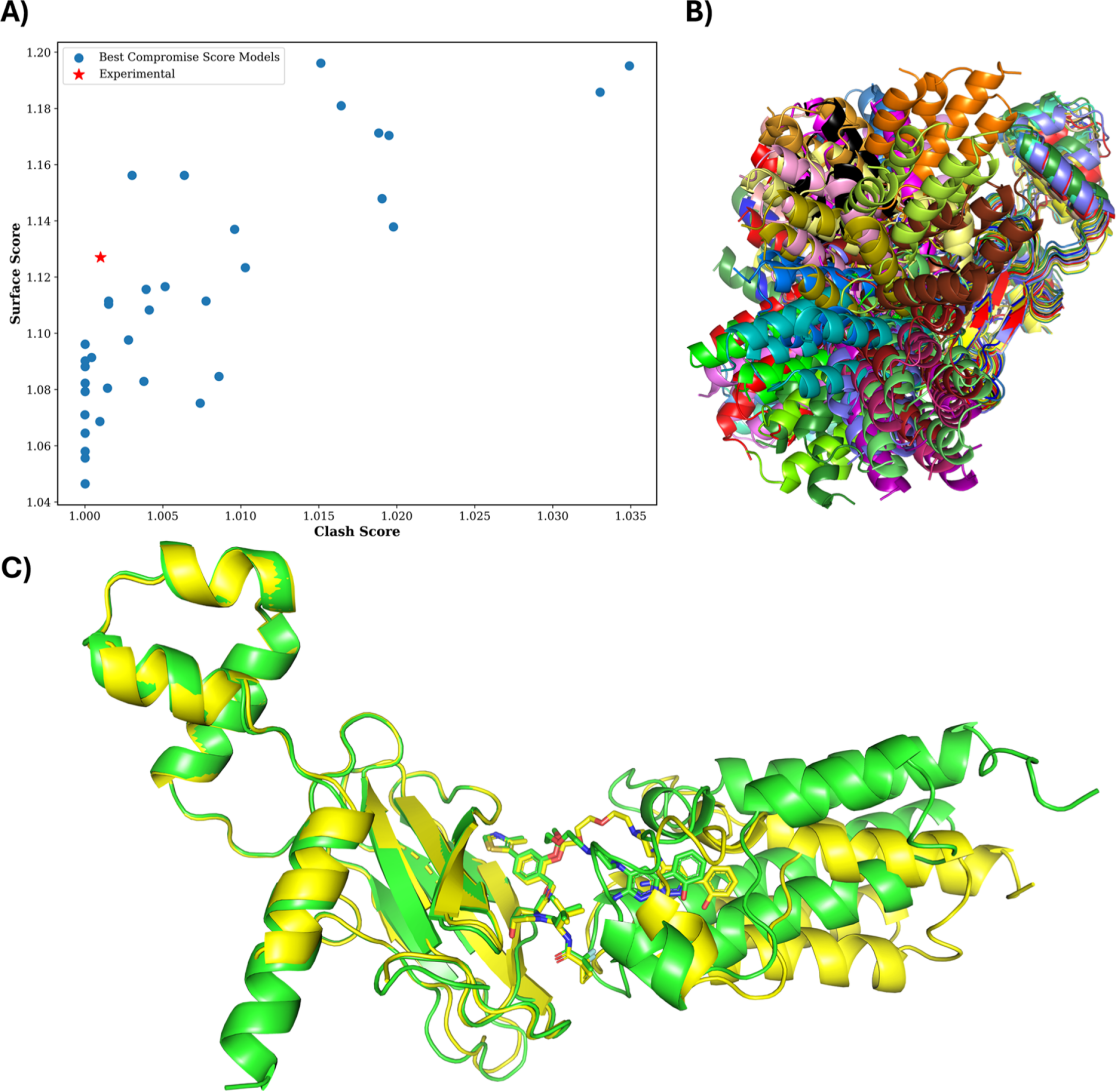
Ensemble reduction for 6HAY. (A) Surface-score vs clash-score scatterplot of the subset models, i.e., those with the best compromise-score for each cluster. (B) Hedgehog-like distribution of the selected models aligned on the E3, illustrating POI orientation diversity. (C) Structural overlay of the TC crystal structure (green) and the most similar model among the selected subset (yellow). The structures were aligned on the E3 using PyMOL (Cα atoms; ligand excluded).^83^

Notably, the lowest pp_RMSD model is not among the selected ones. However, as shown in Figure 9C, one of the selected models presents a POI-E3 orientation very similar to the experimental structure, with a pp_RMSD of 5.12 Å.

If no prior information about the TC structure of interest is available, this method provides a reliable way to obtain good quality models with diverse relative E3-POI orientations, increasing the chance of including a conformation close to the one inducing POI degradation. The reduced ensemble size should also facilitate downstream modeling, such as MD to recover the protein flexibility that the PCG lacks or energy-based relaxation and scoring methods.

### Complementarity with Other Modeling Approaches

Several other methods have been developed to model PROTAC-mediated TCs, and they typically adopt more sophisticated strategies than our rigid-body approach. Notable examples include Method 4B by Drummond et al.,^38^ PRosettaC,^45^ PROTAC-Model,^44^ and PRODE.^48^ Each of these tools integrates protein–protein docking, conformational sampling, and scoring in different ways, often with a focus on structural refinement and energy-based evaluation.

It should be stressed that our goal is not to compete with these methods but rather to provide a complementary tool. Our approach is particularly well-suited for generating diverse and plausible starting models quickly, which can be valuable for downstream applications such as MD simulations or for linker modeling within protein–protein docking workflows. The ability of our method to produce crystal-like TC models that align with those accepted by more advanced protocols is encouraging, especially considering the speed and simplicity of our workflow. Future improvements in scoring and ranking, along with the incorporation of protein flexibility, could make the PCG an even more powerful asset for early stage PROTAC design and modeling.

## Conclusions

We have presented PCG: a knowledge-based protocol and tool for modeling the conformational space of chimeric degraders and producing simple rigid-body-assembled TC models. It enables the fast and robust generation of degraders’ conformational ensembles by leveraging CSD-derived torsional preferences to efficiently explore conformational space.

Our thorough validation using real PROTAC structures from the PDB and PROTAC-like molecules from the CSD demonstrates the reliability of our approach across both experimental and proxy datasets. The scale and detail of this validation help establish a reference framework and offer practical guidance for degrader modeling. Despite the size and complexity of the systems and the simplicity of our approach, the PCG reproduced PROTAC experimental structures in 76% of the cases (lig_RMSD <2 Å) and generated TC models with correct POI-E3 orientation in 90% of the cases (pp_RMSD <10 Å). These models can be obtained quickly and inexpensively, providing a strong starting point for PROTAC drug discovery pipelines involving further refinement steps (e.g., protein relaxation, energetics, dynamics, and property calculation). The PCG can also support lead optimization, enabling users to explore analogues with alternative linker designs.

Thanks to its speed, the PCG allows users to generate very large ensembles and to prioritize the most plausible models with the provided scores. We also proposed a clustering-based workflow to downsize ensembles while preserving the POI-E3 orientation diversity, increasing the likelihood of capturing degradation-relevant conformations in the absence of prior structural information.

Future improvement efforts will aim to address current limitations, particularly by incorporating protein flexibility (e.g., via MD or local minimization) and enhancing model ranking and scoring (e.g., via improved metrics, scoring function, or energy-based methods). Another interesting direction would be to estimate which conformations are more likely to result in POI ubiquitination with machine learning approaches. Finally, it would be valuable to reassess the PCG performance as new structural data become available, especially to evaluate performance across diverse E3-POI systems and to assess the potential influence of crystal packing using cryoEM or solution-phase techniques (e.g., HDX-MS or NMR) derived structures.

## Supplementary Material



## Data Availability

The PROTAC Conformer Generator code and the structures of the PROTAC-like molecules extracted from the CSD are available on GitHub at https://github.com/ccdc-opensource/science-paper-protac-conformer-generator-2025.

## Notes

Structural formulas of PROTACs in the PCG primary validation dataset; geometric and topological properties of PROTAC linkers; automated identification of warhead and E3 recruiting group substructures; selection of PCA features for mining of PROTAC-like molecules from the CSD; selection of PROTAC-like molecules from PCA results; comparison of linker complexity between PROTACs and PROTAC-like molecules; lig_RMSD of closest conformers at different conformational sampling extent; comparison between experimental structures and closest conformers energies; surface-score vs clash-score scatterplots for all TC ensembles; overlay of generated TC models to the experimental structures; and clustering and ensemble reduction for 6HAY (PDF)

## †

Vanderbilt University, Department of Cell and Development Biology, Medical Research Building III, 465 21st Avenue South Nashville, Tennessee 37232, United States

## Abbreviations

BTK: Bruton’s tyrosine kinase
CSD: Cambridge Structural Database
CRBN: cereblon
cryoEM: cryo-electron microscopy
E3: E3 ubiquitin ligase
FF: force-field
HDX-MS: hydrogen/deuterium exchange mass spectrometry
KDE: kernel density estimation
MD: molecular dynamics
NMR: nuclear magnetic resonance
PCG: PROTACs conformer generator
PCA: principal component analysis
PC: principal component
PDB: protein data bank
POI: protein of interest
PROTAC: proteolysis-targeting chimera
RMSD: root-mean-square deviation
PPIs: protein–protein interactions.
