# Supplementary material for: Data-Driven Generation of Conformational Ensembles and Ternary Complexes for PROTAC and Other Chimera Systems
Source: J Chem Inf Model. 2025 Sep 5;65(18):9541–56. doi: 10.1021/acs.jcim.5c00880 (PMC12703653; doi:10.1021/acs.jcim.5c00880)
Supplement: Supplementary file 1 [file ci5c00880_si_001.pdf]

Supporting Information for "Data-driven generation of conformational ensembles and ternary complexes for PROTAC and other Chimera systems"

*Fabio Montisci, Laura Friggeri, Kepa K. Burusco-Goni, Patrick McCabe, Bojana Popovic, Jason C. Cole**

Cambridge Crystallographic Data Centre, 12 Union Road, Cambridge CB2 1EZ, UK.

* Email: cole@ccdc.cam.ac.uk

Table of Contents

Structural formulas of PROTACs in the PCG primary validation dataset 2
Geometric and topological properties of PROTAC linkers..18
Automated identification of warhead and E3 recruiting group substructures 20
Selection of PCA features for mining of PROTAC-like molecules from the CSD......................21
Selection of PROTAC-like molecules from PCA results 23
Comparison of linker complexity between PROTACs and PROTAC-like molecules 27
Lig_RMSD of closest conformers at different conformational sampling extent 29
Comparison between experimental structures and closest conformers energies..........................33
Surface-score vs clash-score scatterplots for all ternary complex ensembles 38
Overlay of generated ternary complex models to the experimental structures 67
Clustering and ensemble reduction for 6HAY 98

Structural formulas of PROTACs in the PCG primary validation dataset

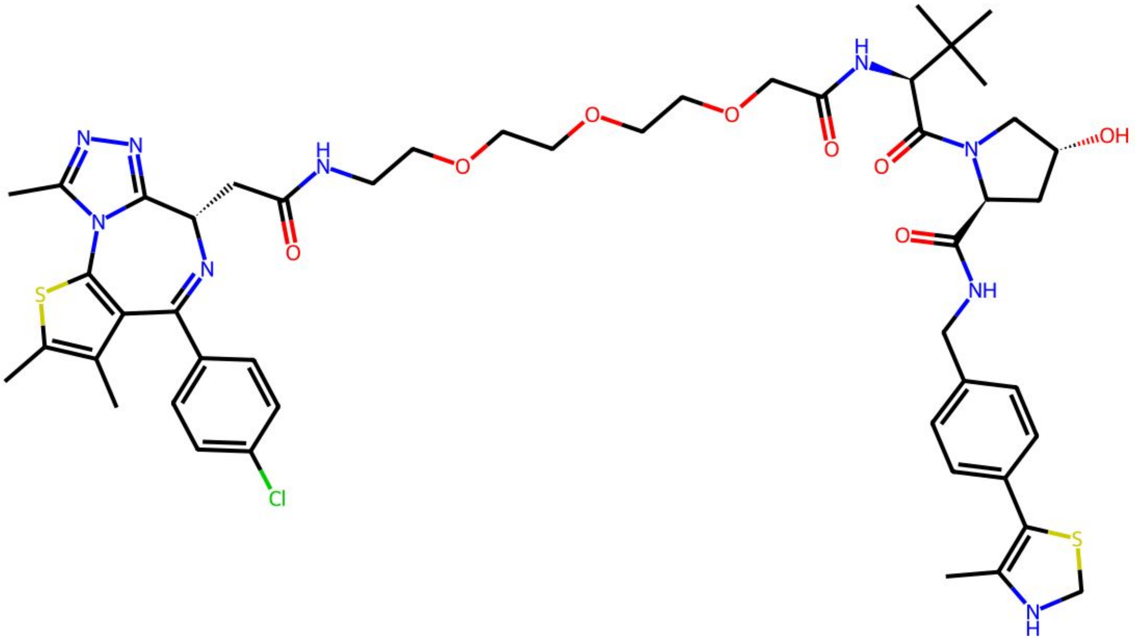

Figure S1. 5T35 PROTAC ligand structure.

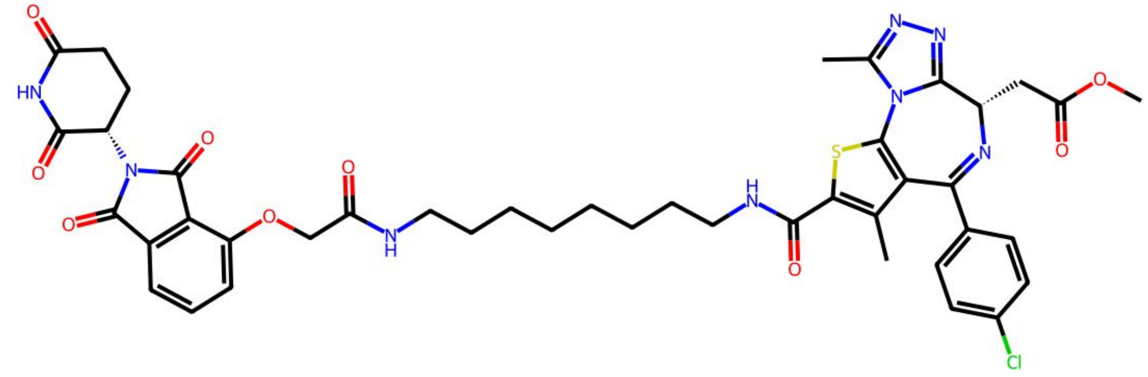

Figure S2. 6BN7 PROTAC ligand structure.

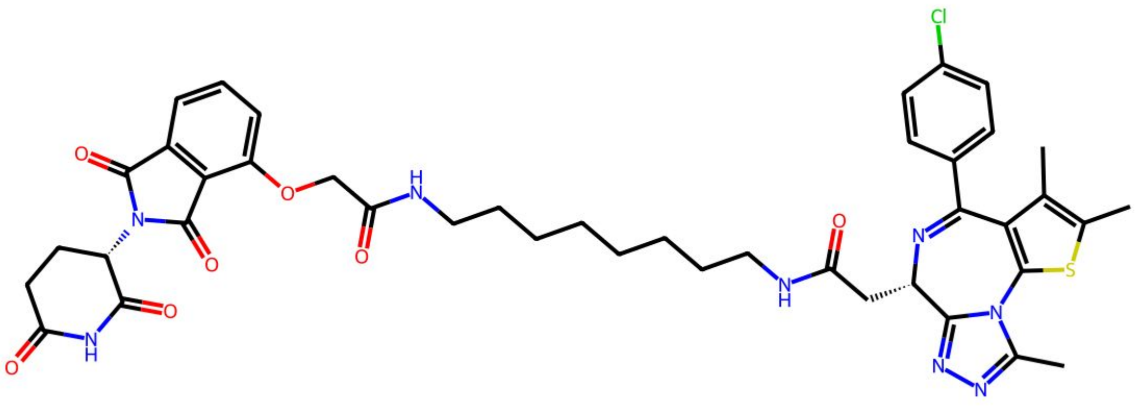

Figure S3. 6BOY PROTAC ligand structure.

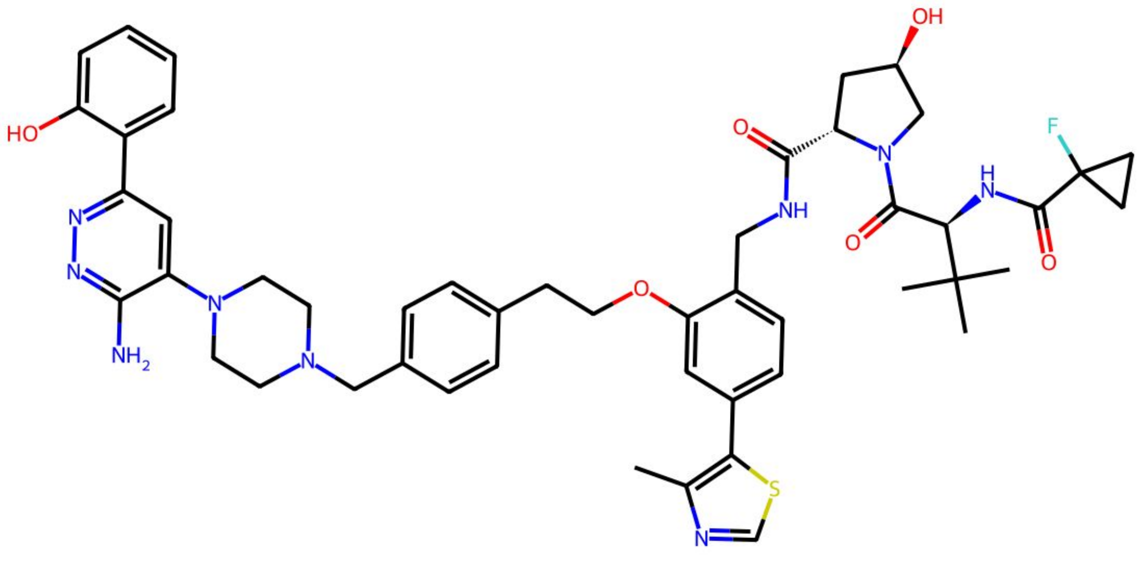

Figure S4. 6HAX PROTAC ligand structure.

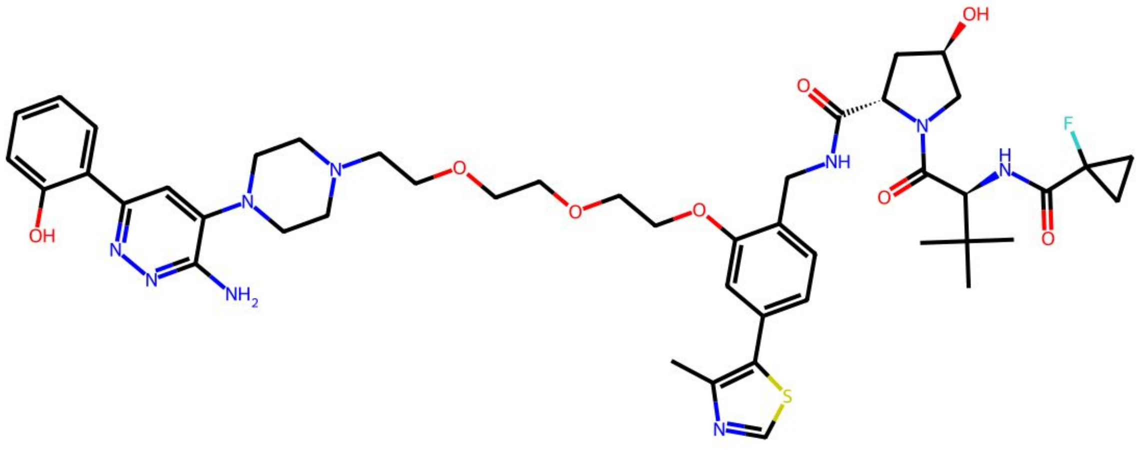

Figure S5. 6HAY PROTAC ligand structure.

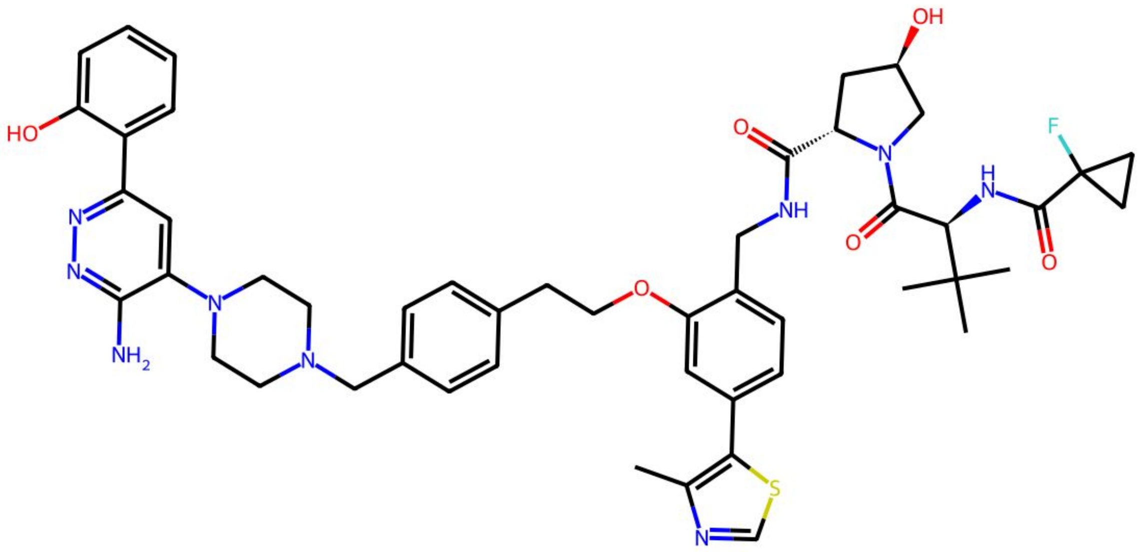

Figure S6. 6HR2 PROTAC ligand structure.

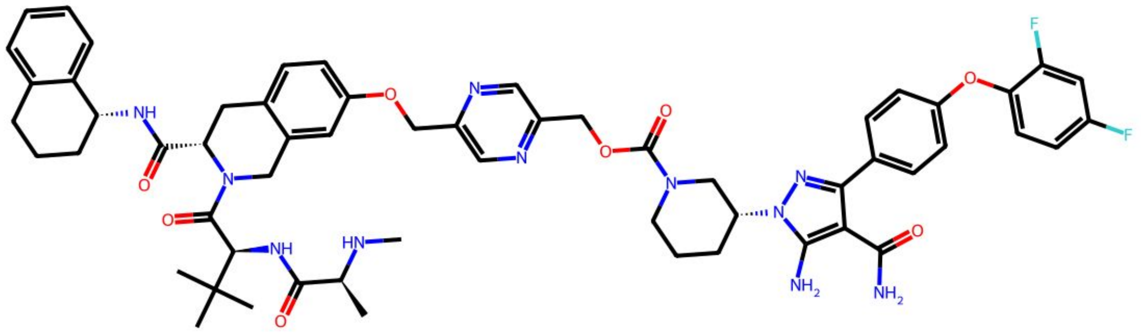

Figure S7. 6W7O PROTAC ligand structure.

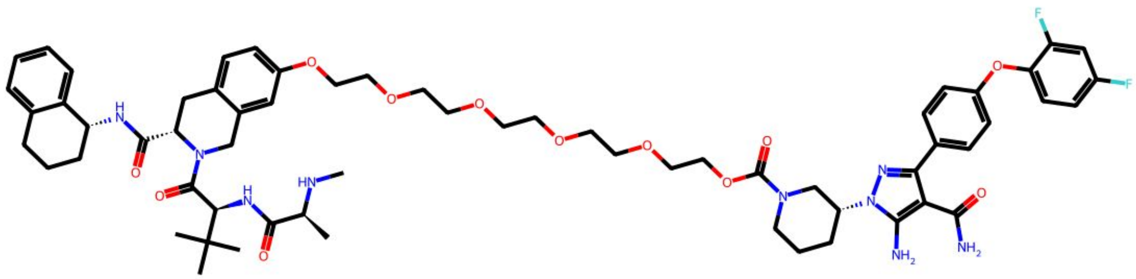

Figure S8. 6W8I PROTAC ligand structure.

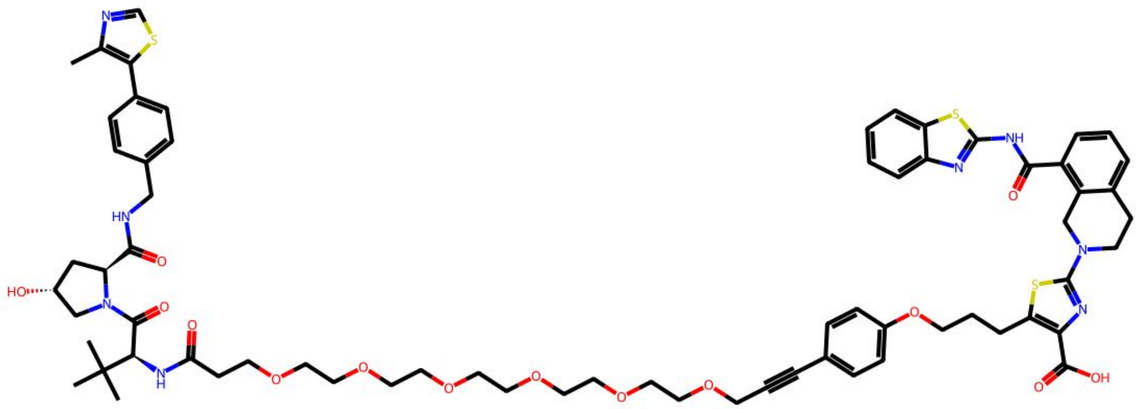

Figure S9. 6ZHC PROTAC ligand structure.

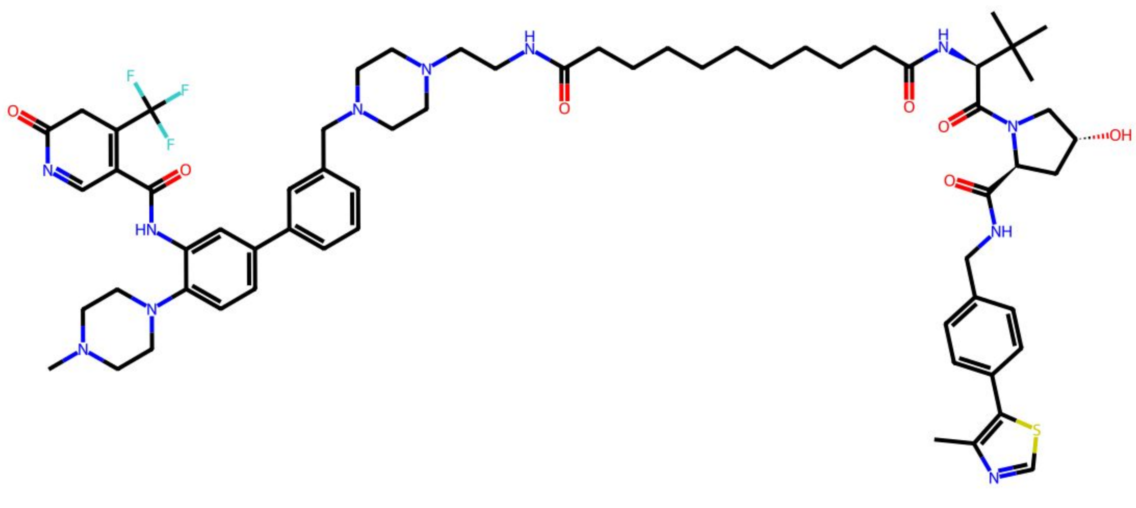

Figure S10. 7JTO PROTAC ligand structure.

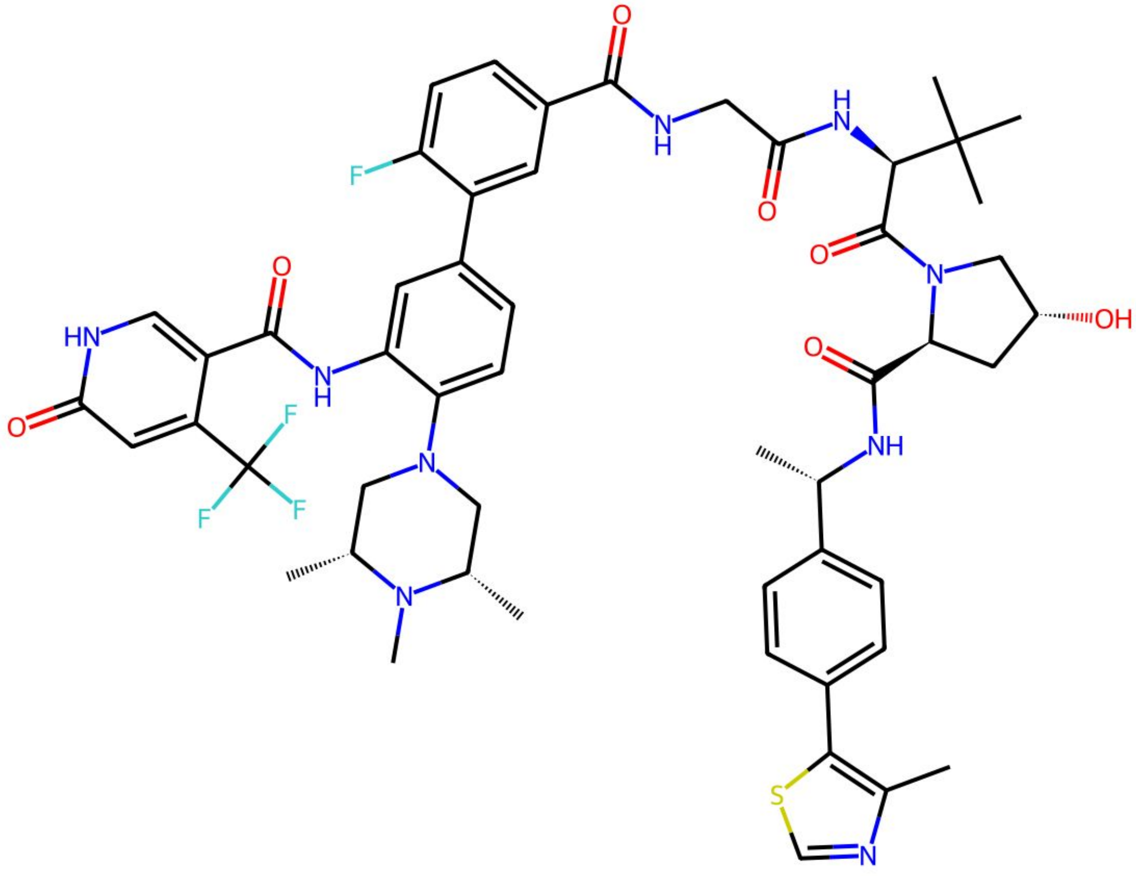

Figure S11. 7JTP PROTAC ligand structure.

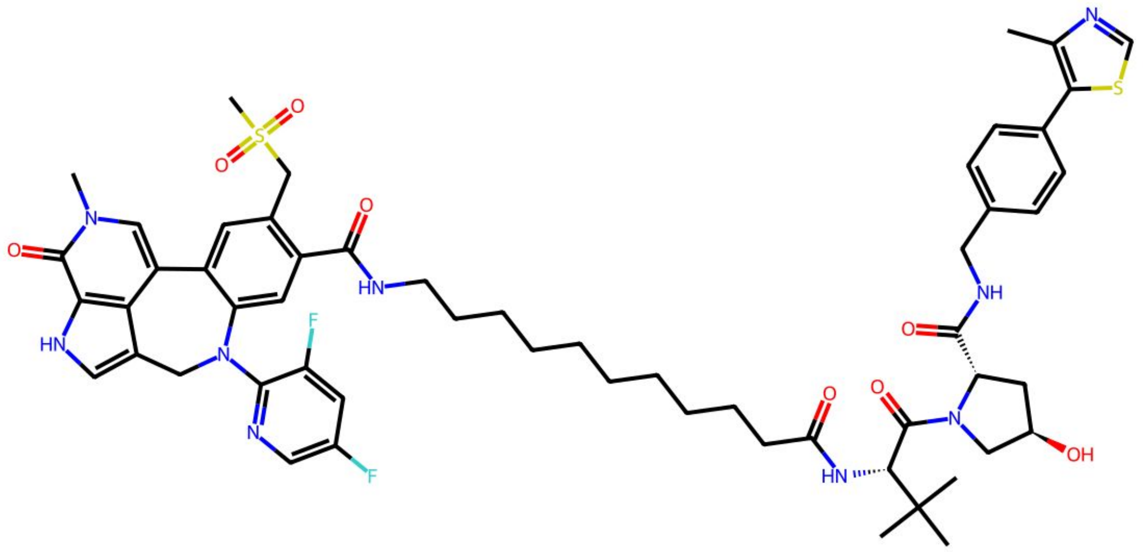

Figure S12. 7KHH PROTAC ligand structure.

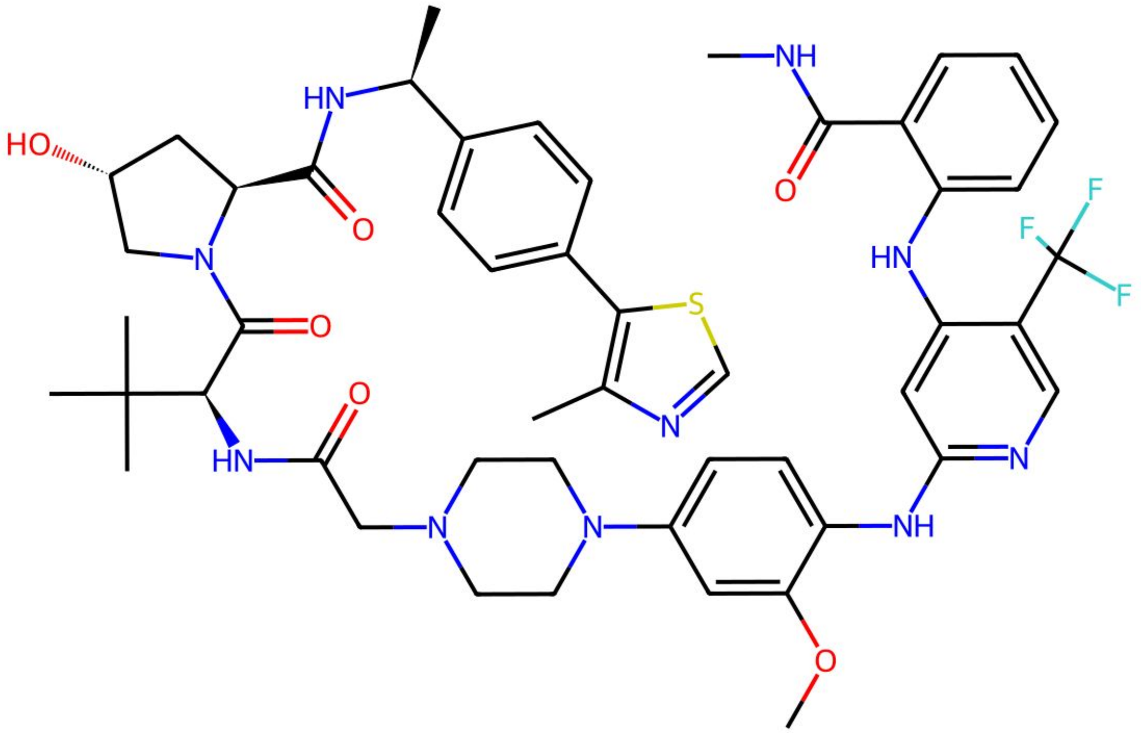

Figure S13. 7PI4 PROTAC ligand structure.

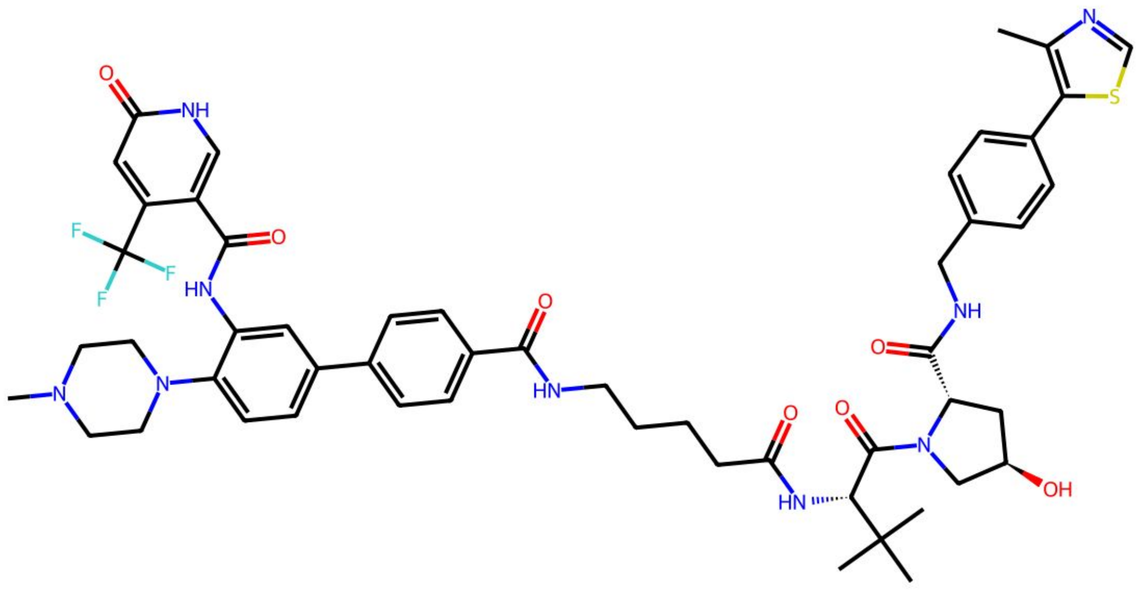

Figure S14. 7Q2J PROTAC ligand structure.

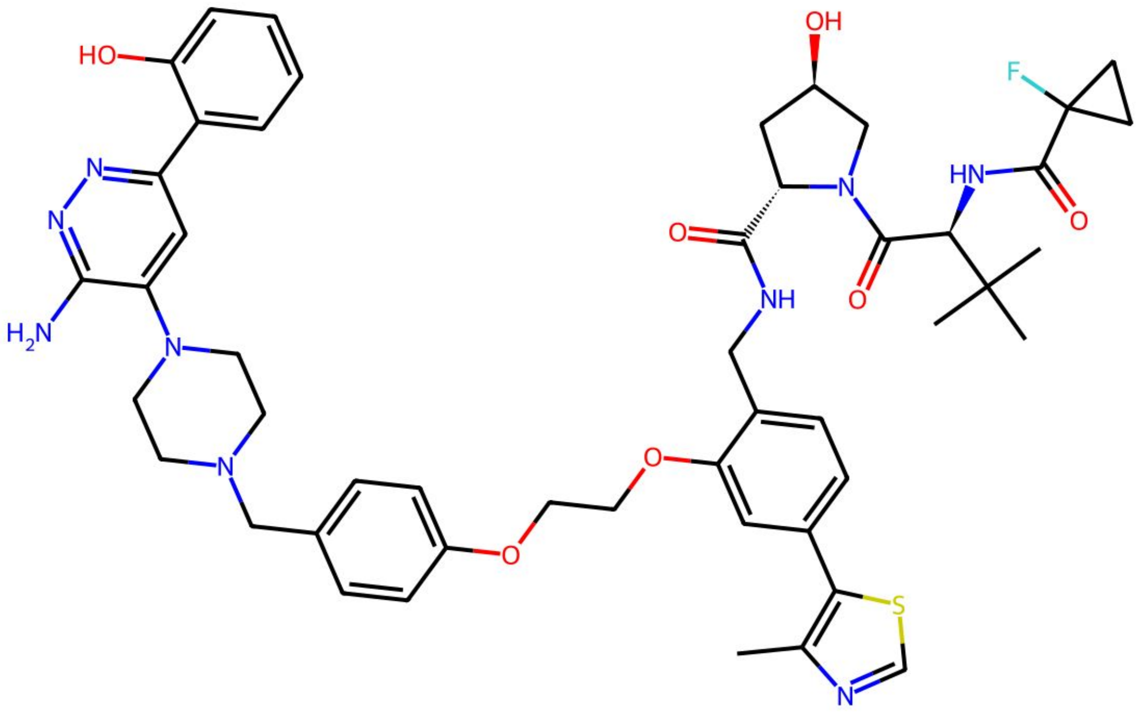

Figure S15. 7S4E PROTAC ligand structure.

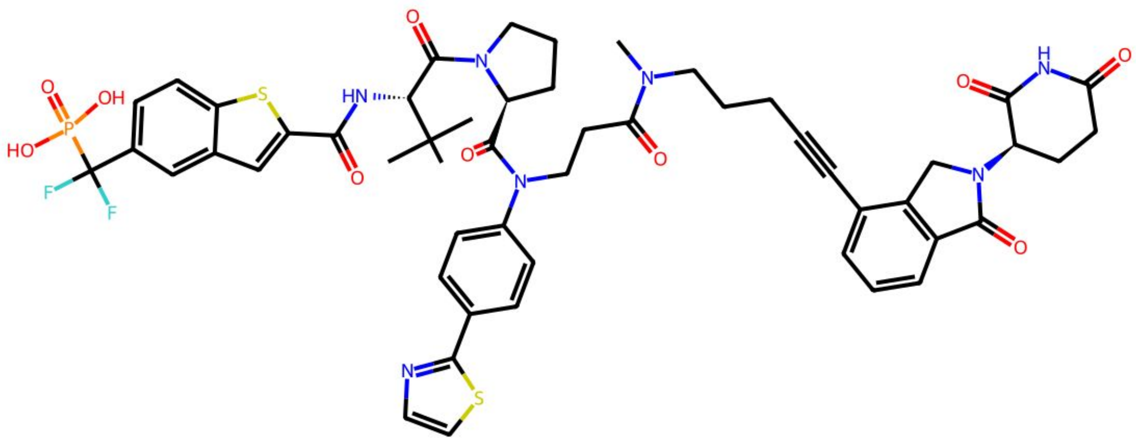

Figure S16. 7TVA PROTAC ligand structure.

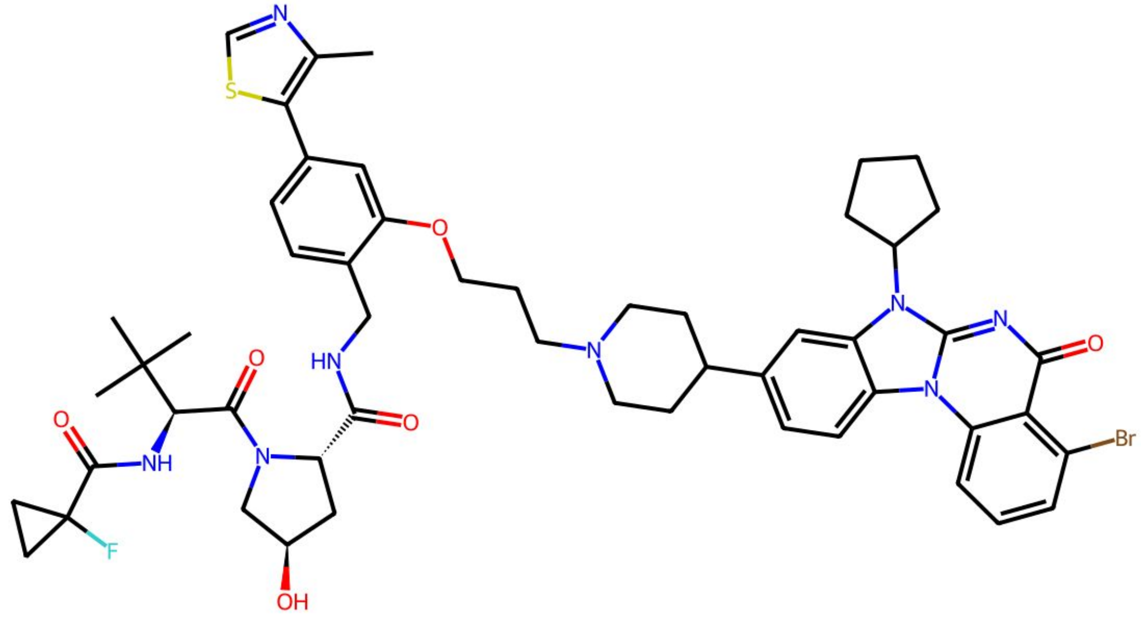

Figure S17. 7Z6L PROTAC ligand structure.

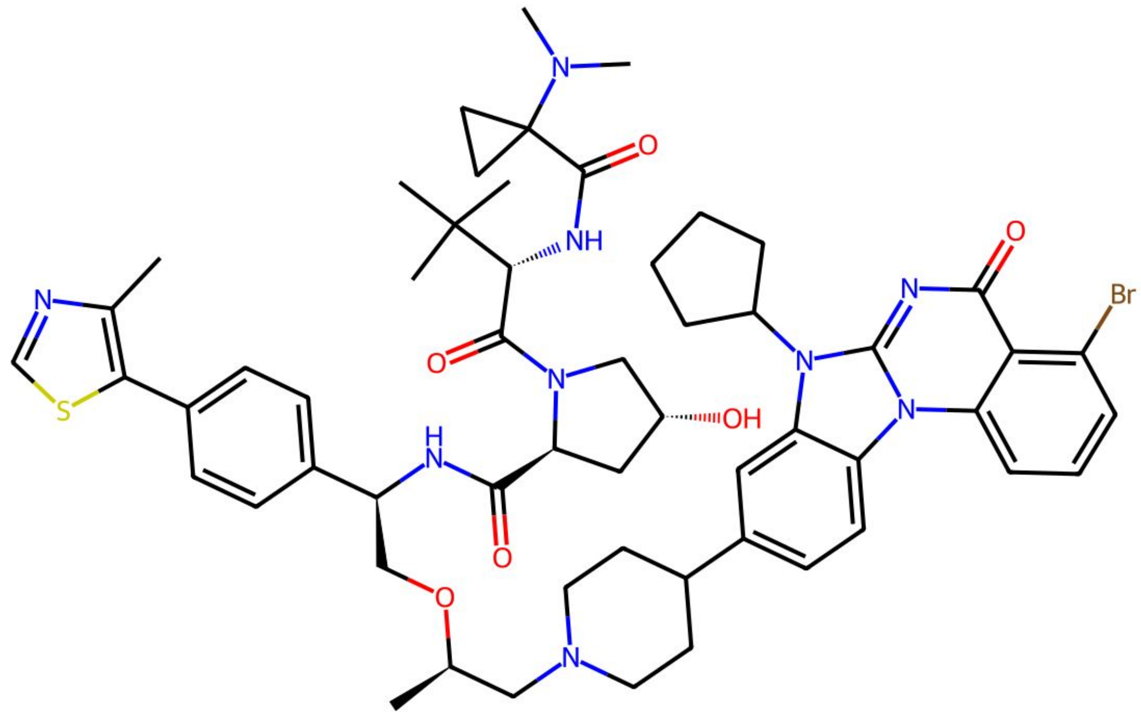

Figure S18. 7Z76 PROTAC ligand structure.

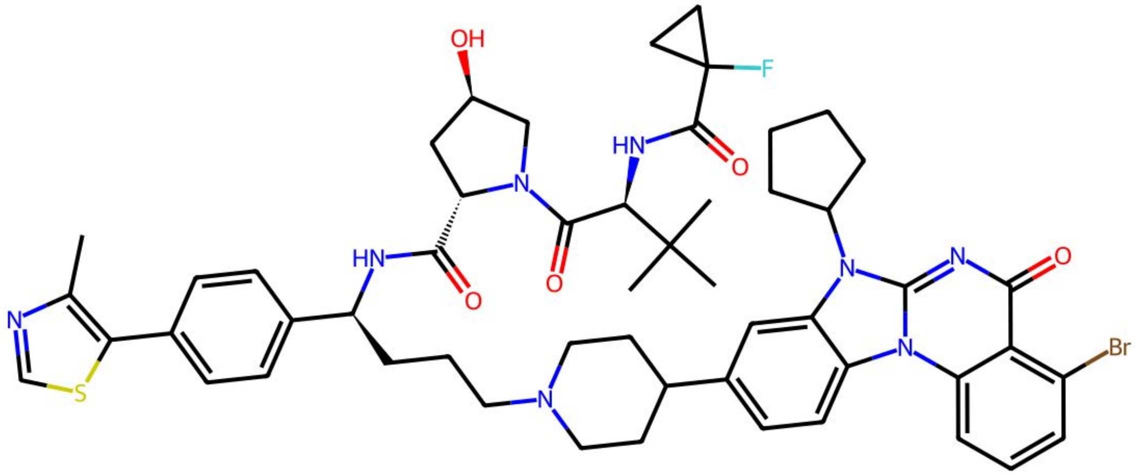

Figure S19. 7Z77 PROTAC ligand structure.

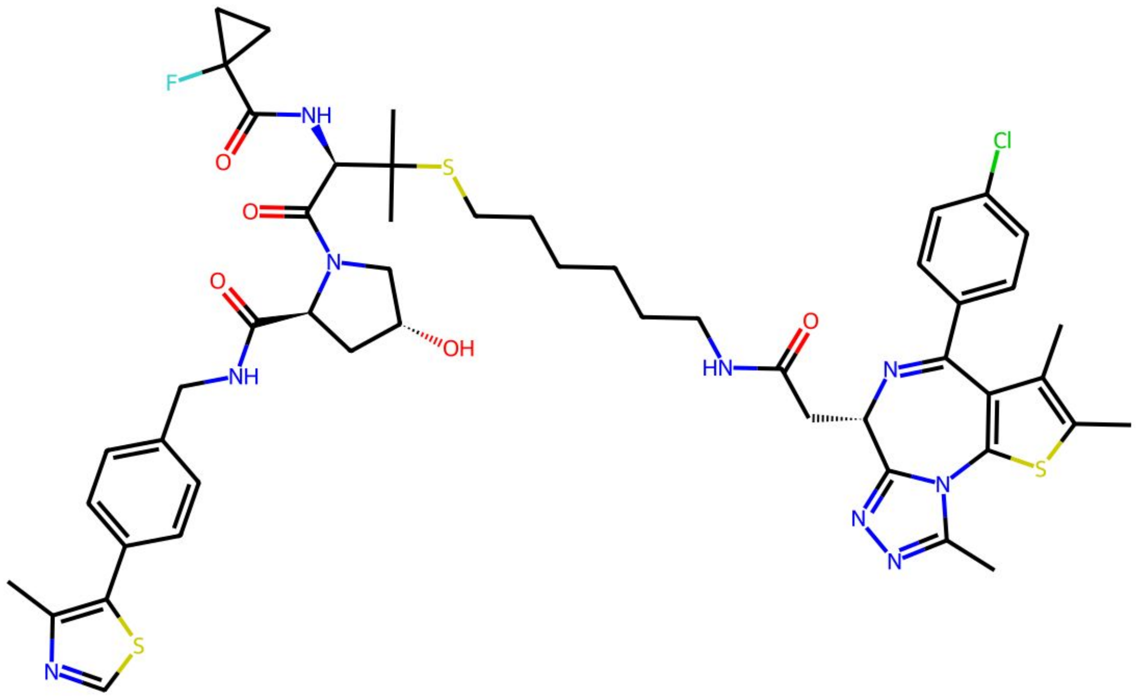

Figure S20. 7ZNT PROTAC ligand structure.

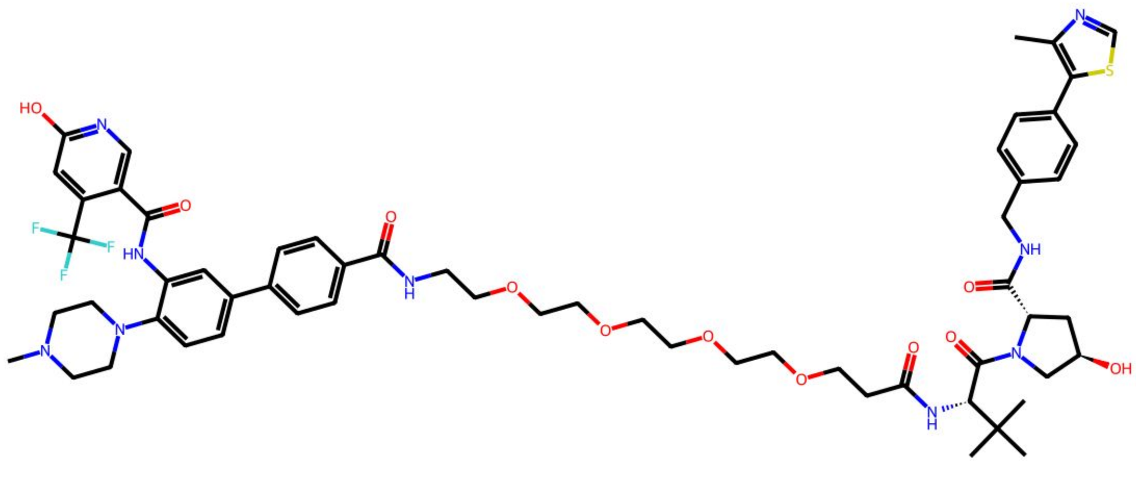

Figure S21. 8BB2 PROTAC ligand structure.

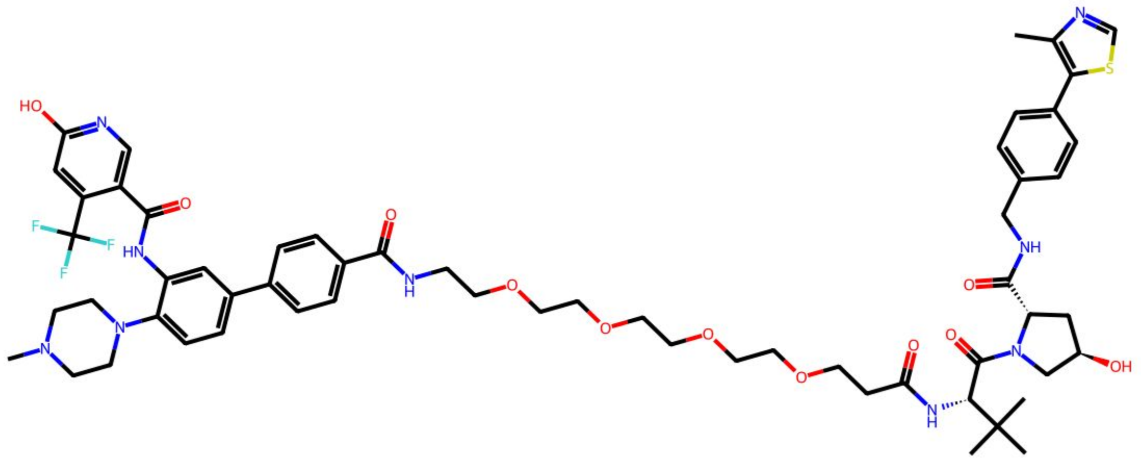

Figure S22. 8BB3 PROTAC ligand structure.

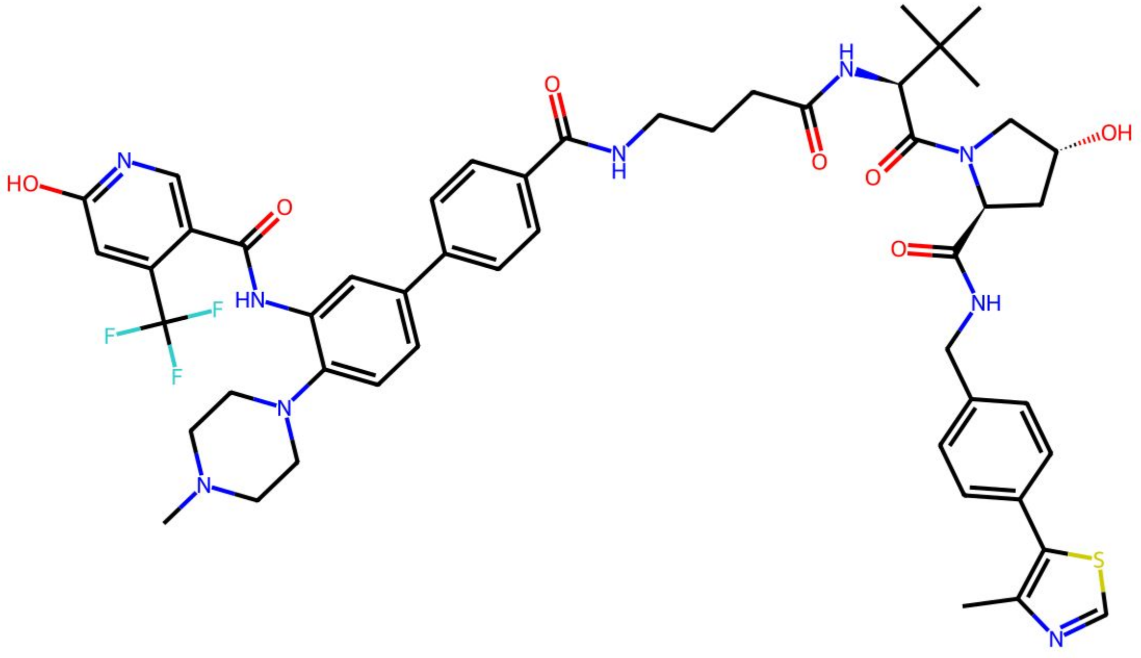

Figure S23. 8BB4 PROTAC ligand structure.

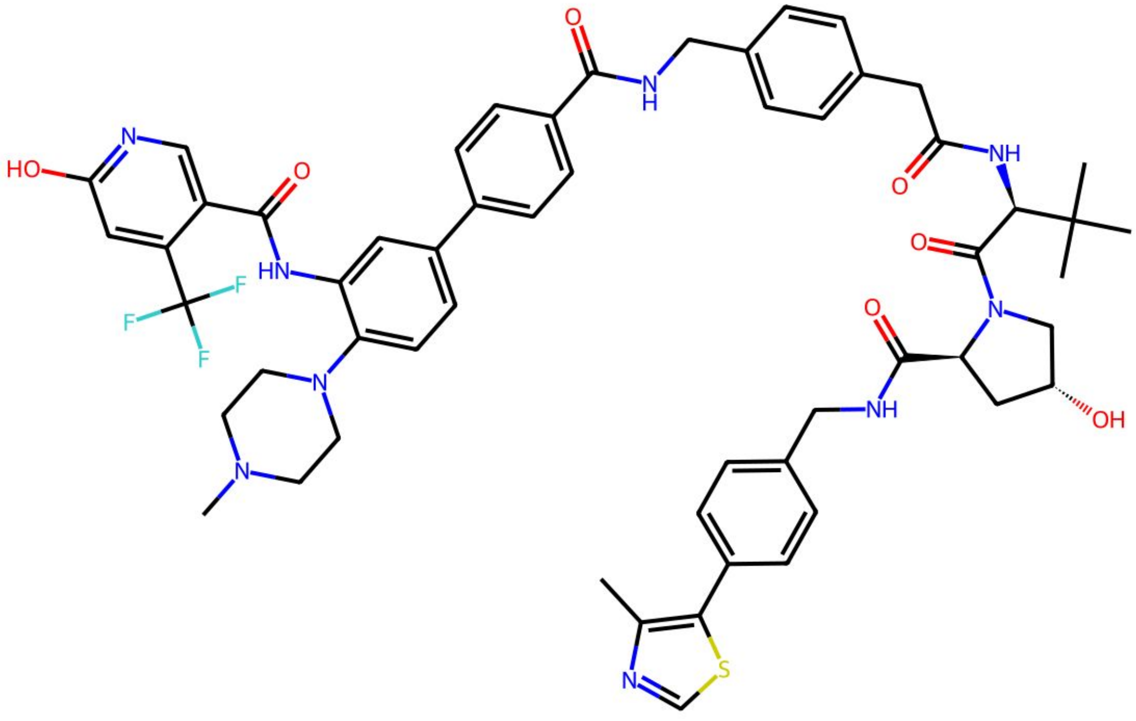

Figure S24. 8BB5 PROTAC ligand structure.

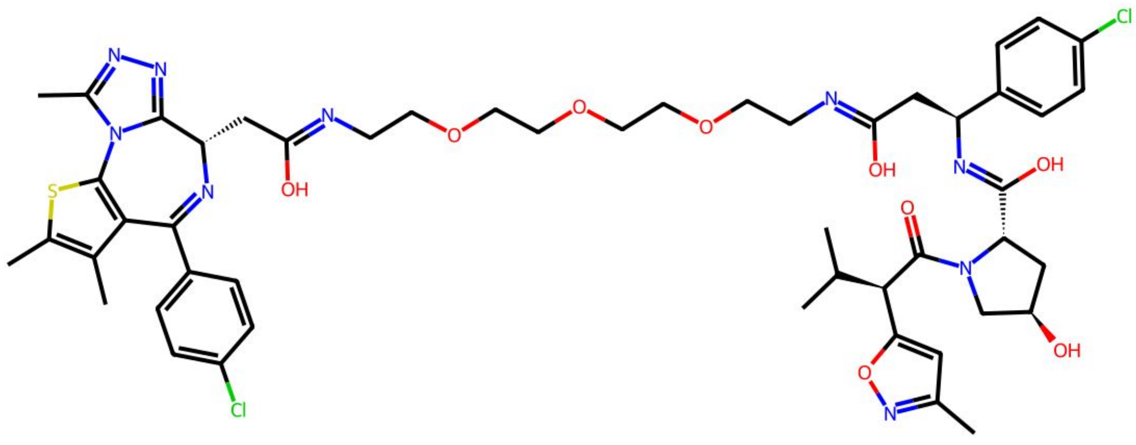

Figure S25. 8BDS PROTAC ligand structure.

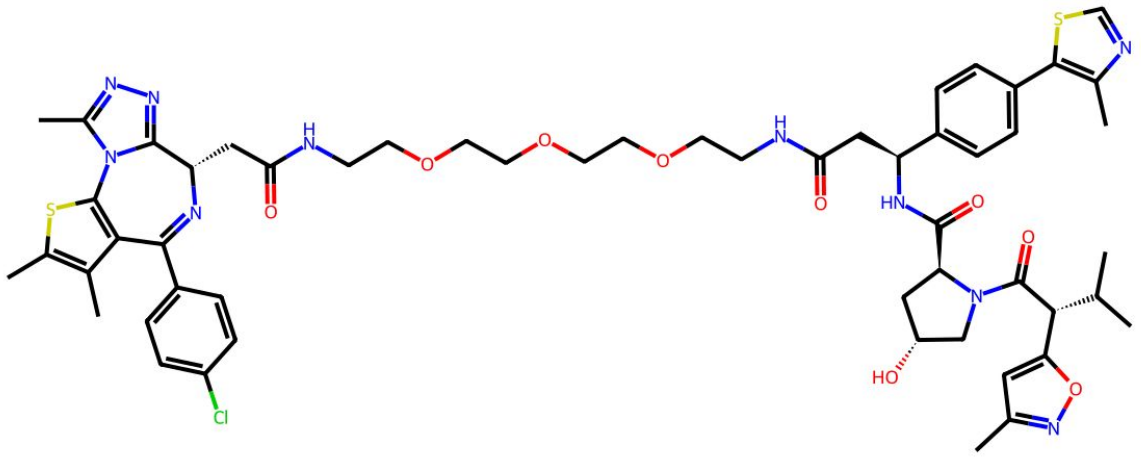

Figure S26. 8BDT PROTAC ligand structure.

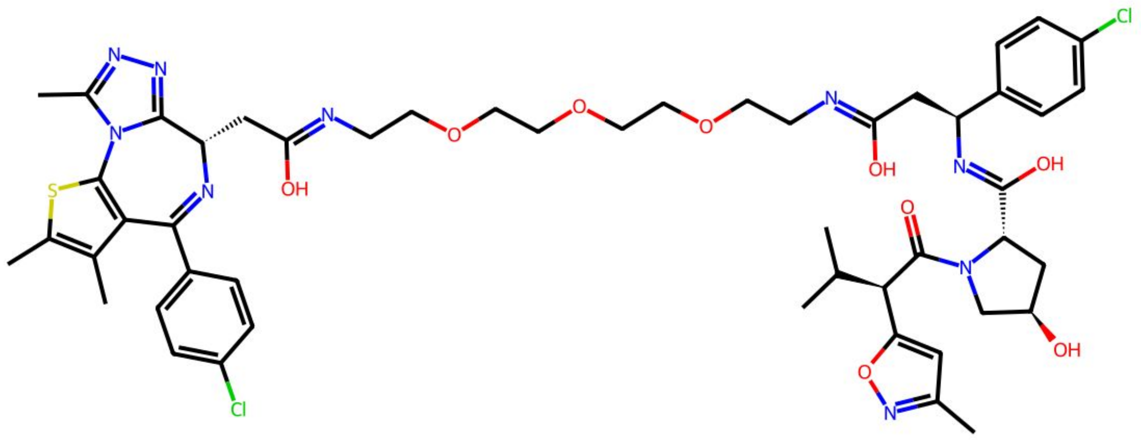

Figure S27. 8BDX PROTAC ligand structure.

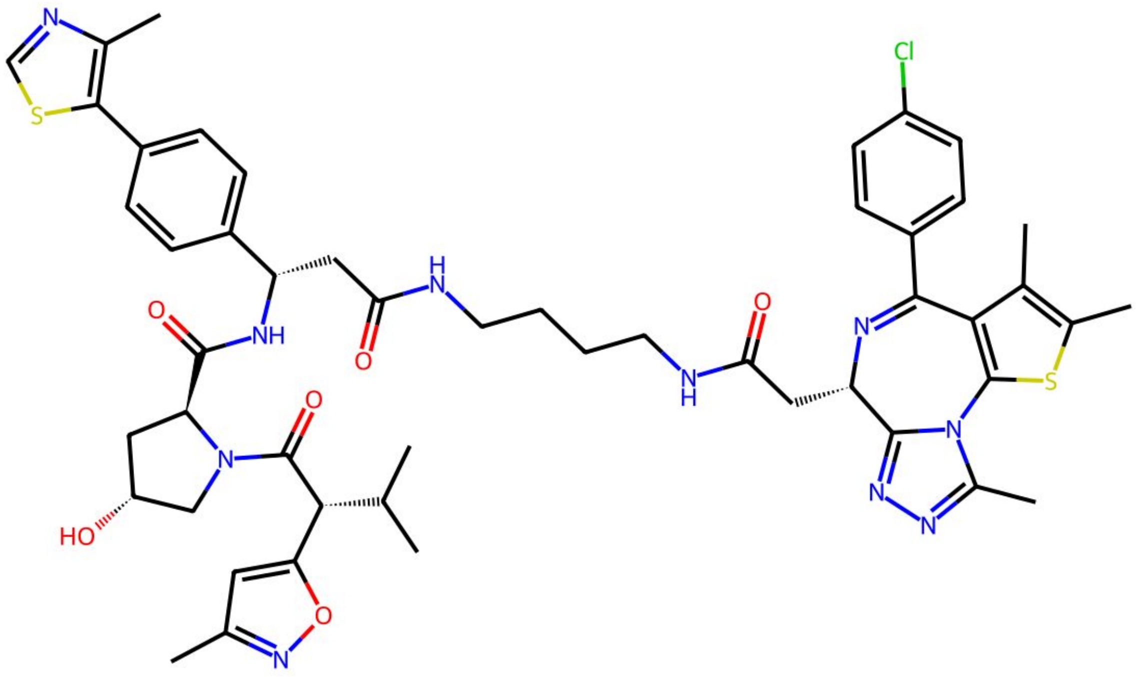

Figure S28. 8BEB PROTAC ligand structure.

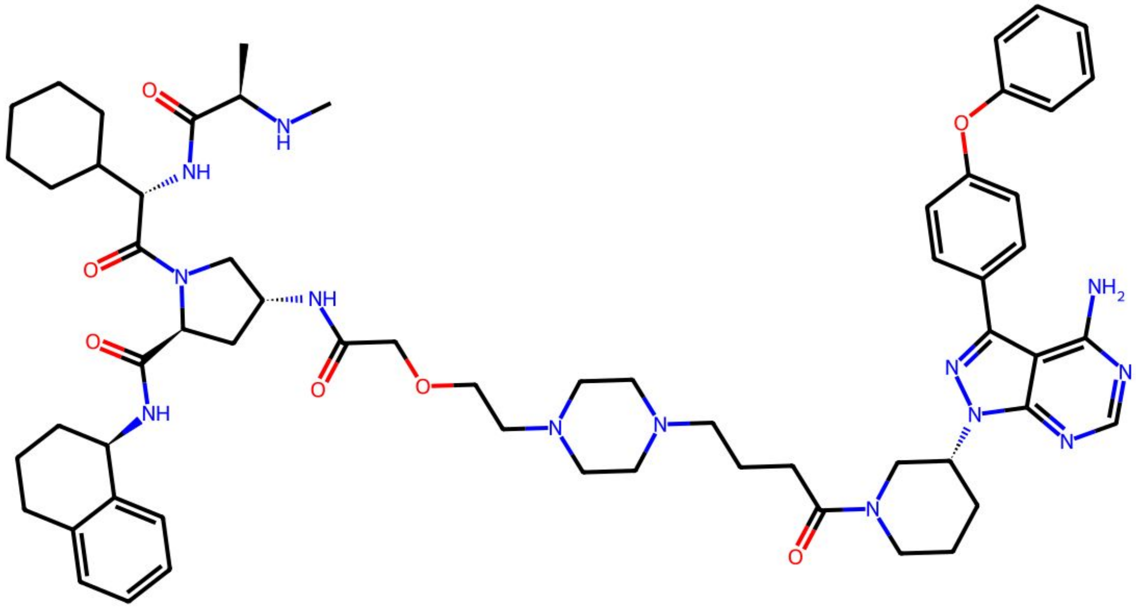

Figure S29. 8DSO PROTAC ligand structure.

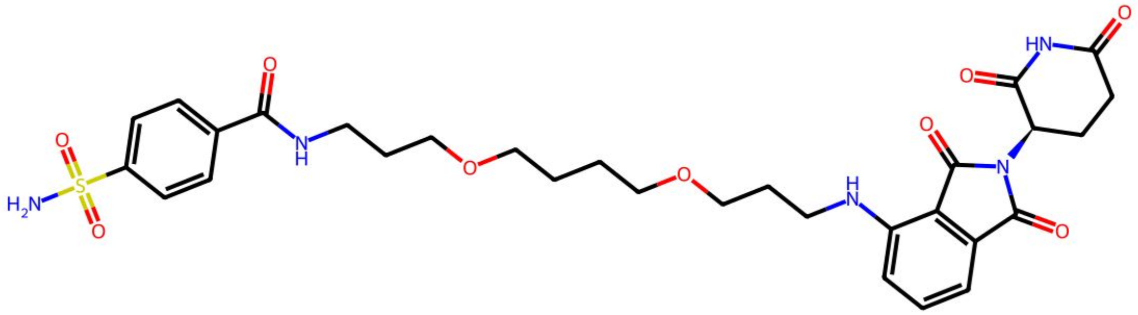

Figure S30. 8EXC PROTAC ligand structure.

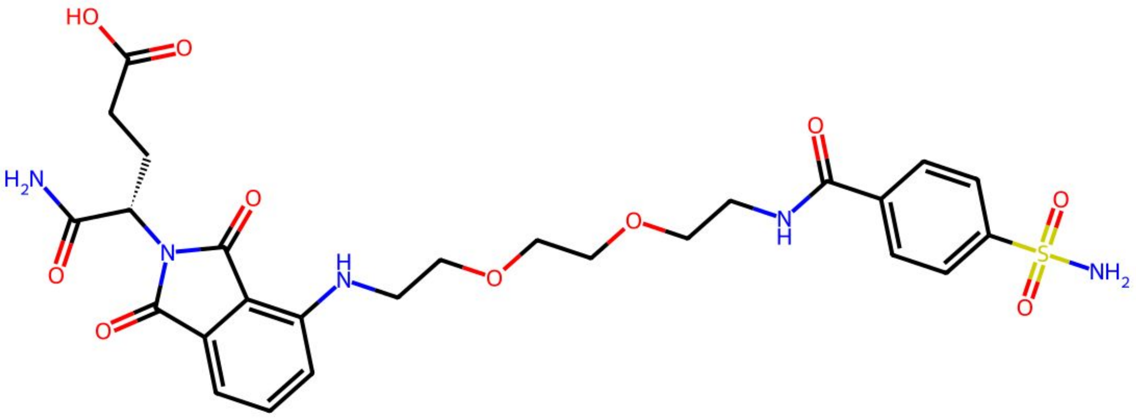

Figure S31. 8EXG PROTAC ligand structure.

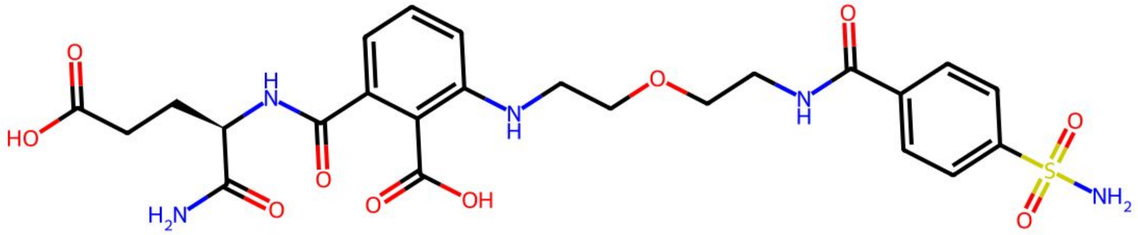

Figure S32. 8EYL PROTAC ligand structure.

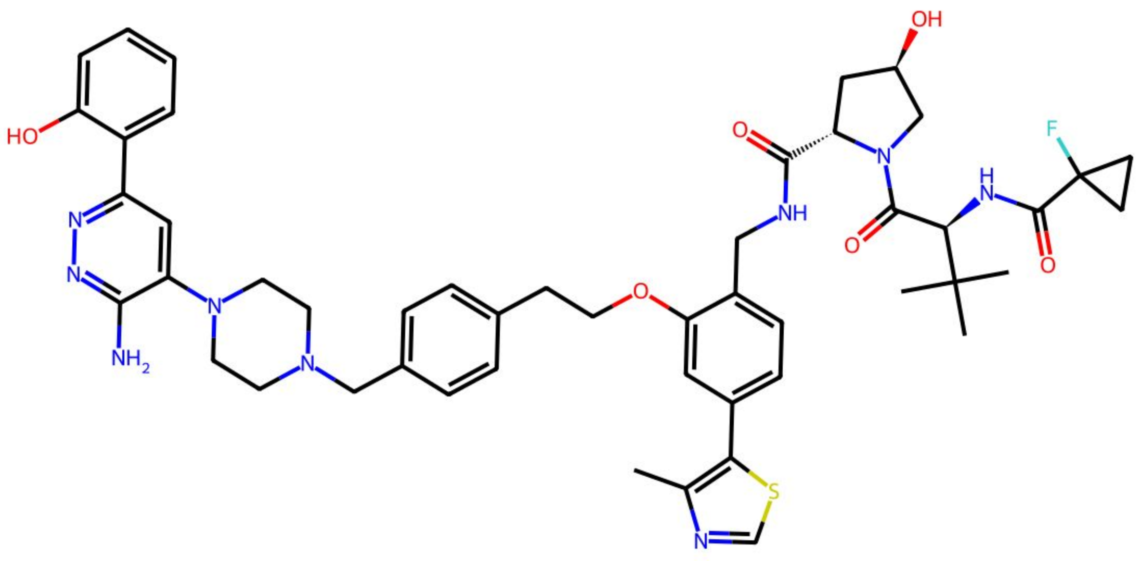

Figure S33. 8G1P PROTAC ligand structure.

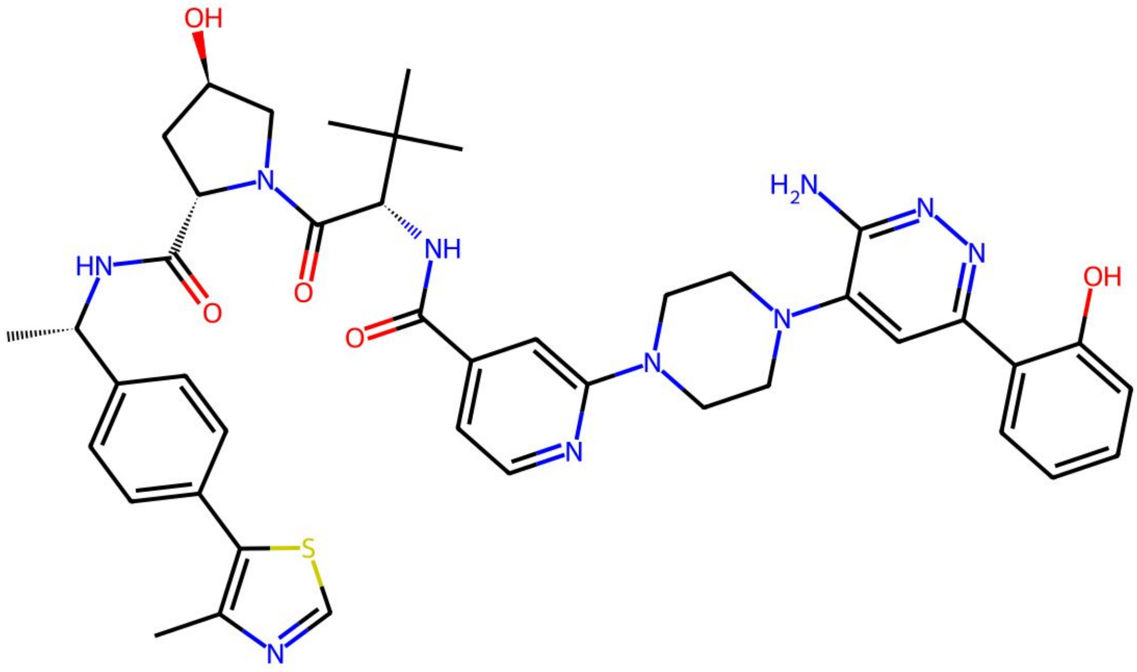

Figure S34. 8G1Q PROTAC ligand structure.

Geometric and topological properties of PROTAC linkers

To quantify the spatial and structural characteristics of PROTAC linkers in our dataset, we computed a set of complementary geometric descriptors using the CSD Python API. The *linker diameter* is defined as the maximum Euclidean distance between any two heavy atoms in the linker, capturing its spatial extent in the crystallographic conformation. The *longest bond path* represents the maximum cumulative bond length along any covalently connected path of heavy atoms, reflecting the potential maximum span of the linker backbone across different conformations. These geometric descriptors are complemented by topological parameters, including the number of heavy atoms, acyclic rotatable bonds, and cyclic bonds. Together, these metrics provide a chemically meaningful summary of linker complexity and conformational reach.

Table S1. Summary of linker properties for each PROTAC in the primary validation set. The linker diameter is the maximum interatomic distance within the linker's 3D structure, while the longest bond path is the maximum cumulative bond length along a covalent path. The number of heavy atoms, acyclic rotatable bonds, and cyclic bonds are also reported to characterize linker complexity.

	Diameter (Å)	Longest bond path (Å)	Heavy atoms	Acyclic rotatable bonds	Cyclic bonds
**5T35**	8.8	16.0	12	11	0

6BN7	11.1	20.5	16	14	0
6BOY	13.7	20.5	16	14	0
6HAX	7.9	13.0	12	6	6
6HAY	8.3	14.5	11	10	0
6HR2	7.9	13.0	12	6	6
6W7O	8.5	12.7	12	6	6
6W8I	13.5	24.5	18	17	0
6ZHC	13.7	27.8	20	19	0
7JTO	12.2	24.3	19	16	0
7JTP	5.9	7.1	8	5	0
7KHH	9.1	16.5	12	11	0
7PI4	5.6	8.9	8	2	6
7Q2J	6.8	8.9	8	6	0
7S4E	9.8	14.3	13	7	6
7TVA	6.9	8.6	7	5	0
7Z6L	5.0	7.4	6	5	0
7Z76	12.9	22.2	20	10	11
7Z77	4.5	6.1	5	4	0
7ZNT	9.6	14.0	10	9	0
8BB2	13.5	23.3	18	16	0
8BB3	11.6	23.2	18	16	0
8BB4	5.9	7.4	7	5	0
8BB5	8.1	12.8	12	5	6
8BDS	10.2	23.2	18	16	0
8BDT	8.0	23.1	18	16	0

**8BDX**	8.5	23.2	18	16	0
**8DSO**	7.0	10.3	9	7	0
**8G1P**	12.0	17.6	16	9	6
**8G1Q**	13.6	17.9	13	12	0

Automated identification of warhead and E3 recruiting group substructures

The input files for the PCG were prepared automatically using a Python API script, following the procedure described in the Materials and Methods section of the main manuscript. This section expands on the steps used to programmatically identify which of the two PCG protein inputs corresponds to the POI and which to the E3 ligase, and to extract the corresponding warhead and E3 recruiting group fragments from the PROTAC ligand. First, a substructure search using the linker-containing SMARTS code provided as input is performed. A tag is attached to the atoms neighboring the "link-atoms", i.e., the first and last atoms of the linker (labeled [:1] and [:2] in the SMARTS code). The link-atoms are then removed, splitting the PROTAC ligand into three fragments. The fragment containing the highest number of tagged neighbor atoms is identified as the linker and excluded from further processing. Crossed maximum common substructure searches between the two remaining fragments and the original E3 ligase and POI ligands are used to assign the warhead and E3 recruiting group labels, after which the fragments are protonated and saved as mol2 files.

Selection of PCA features for mining of PROTAC-like molecules from the CSD

The following 124 2D molecular descriptors were calculated using RDKit for both the PROTACs from PROTAC-DB and the selected set of molecules from the CSD crystal structures:

MaxAbsEStateIndex, MaxEStateIndex, MinAbsEStateIndex, MinEStateIndex, qed, MolWt, HeavyAtomMolWt, ExactMolWt, NumValenceElectrons, NumRadicalElectrons, MaxPartialCharge, MinPartialCharge, MaxAbsPartialCharge, MinAbsPartialCharge, FpDensityMorgan1, FpDensityMorgan2, FpDensityMorgan3, BCUT2D_MWHI, BCUT2D_MWLOW, BCUT2D_CHGHI, BCUT2D_CHGLO, BCUT2D_LOGPHI, BCUT2D_LOGPLOW, BCUT2D_MRHI, BCUT2D_MRLOW, AvgIpc, BalabanJ, BertzCT, Chi0, Chi0n, Chi0v, Chi1, Chi1n, Chi1v, Chi2n, Chi2v, Chi3n, Chi3v, Chi4n, Chi4v, HallKierAlpha, Ipc, Kappa1, Kappa2, Kappa3, LabuteASA, PEOE_VSA1, PEOE_VSA10, PEOE_VSA11, PEOE_VSA12, PEOE_VSA13, PEOE_VSA14, PEOE_VSA2, PEOE_VSA3, PEOE_VSA4, PEOE_VSA5, PEOE_VSA6, PEOE_VSA7, PEOE_VSA8, PEOE_VSA9, SMR_VSA1, SMR_VSA10, SMR_VSA2, SMR_VSA3, SMR_VSA4, SMR_VSA5, SMR_VSA6, SMR_VSA7, SMR_VSA8, SMR_VSA9, SlogP_VSA1, SlogP_VSA10, SlogP_VSA11, SlogP_VSA12, SlogP_VSA2, SlogP_VSA3, SlogP_VSA4, SlogP_VSA5, SlogP_VSA6, SlogP_VSA7, SlogP_VSA8, SlogP_VSA9, TPSA, EState_VSA1, EState_VSA10, EState_VSA11, EState_VSA2, EState_VSA3, EState_VSA4, EState_VSA5,

EState_VSA6, EState_VSA7, EState_VSA8, EState_VSA9, VSA_EState1, VSA_EState10, VSA_EState2, VSA_EState3, VSA_EState4, VSA_EState5, VSA_EState6, VSA_EState7, VSA_EState8, VSA_EState9, FractionCSP3, HeavyAtomCount, NHOHCount, NOCount, NumAliphaticCarbocycles, NumAliphaticHeterocycles, NumAliphaticRings, NumAromaticCarbocycles, NumAromaticHeterocycles, NumAromaticRings, NumHAcceptors, NumHDonors, NumHeteroatoms, NumRotatableBonds, NumSaturatedCarbocycles, NumSaturatedHeterocycles, NumSaturatedRings, RingCount, MolLogP, MolMR.

After descriptor calculation, preprocessing steps were applied to ensure uniformity and reliability prior to PCA. Descriptors with non-numeric values, missing values (NA), or low variance across the dataset (>95% identical values) were excluded. The IPC descriptor was also excluded, as it was not meaningful for PROTAC-sized molecules and produced numerically unstable values.

The remaining 115 descriptors were standardized to zero mean and unit variance using the StandardScaler from the scikit-learn Python library. A correlation matrix was then constructed to quantify pairwise linear relationships between descriptors. For each descriptor, the number of other descriptors with an absolute correlation coefficient ≥ 0.7 was counted, and descriptors were ranked accordingly. The top-ranked descriptor was selected as a feature, and all descriptors highly correlated with it were removed from the pool. This procedure was repeated iteratively until all descriptors were either selected or discarded. The final set of 62 selected features was:

MaxAbsEStateIndex, MinAbsEStateIndex, MinEStateIndex, FpDensityMorgan1, BCUT2D_MWHI, BCUT2D_MWLOW, BCUT2D_CHGLO, BCUT2D_MRLOW, AvgIpc, BalabanJ, Chi4v, Kappa3, PEOE_VSA10, PEOE_VSA11, PEOE_VSA12, PEOE_VSA13, PEOE_VSA14, PEOE_VSA2, PEOE_VSA3, PEOE_VSA4, PEOE_VSA5, PEOE_VSA8, PEOE_VSA9, SMR_VSA10, SMR_VSA2, SMR_VSA3, SMR_VSA5, SMR_VSA6, SMR_VSA9, SlogP_VSA1, SlogP_VSA11, SlogP_VSA3, SlogP_VSA4, SlogP_VSA6, SlogP_VSA7, SlogP_VSA8,

EState_VSA1, EState_VSA10, EState_VSA11, EState_VSA2, EState_VSA3, EState_VSA4, EState_VSA5, EState_VSA6, EState_VSA7, EState_VSA8, EState_VSA9, VSA_EState1, VSA_EState10, VSA_EState3, VSA_EState4, VSA_EState5, VSA_EState7, VSA_EState9, FractionCSP3, NOCount, NumAliphaticCarbocycles, NumAromaticHeterocycles, NumHDonors, NumRotatableBonds, NumSaturatedHeterocycles, MolMR.

Selection of PROTAC-like molecules from PCA results

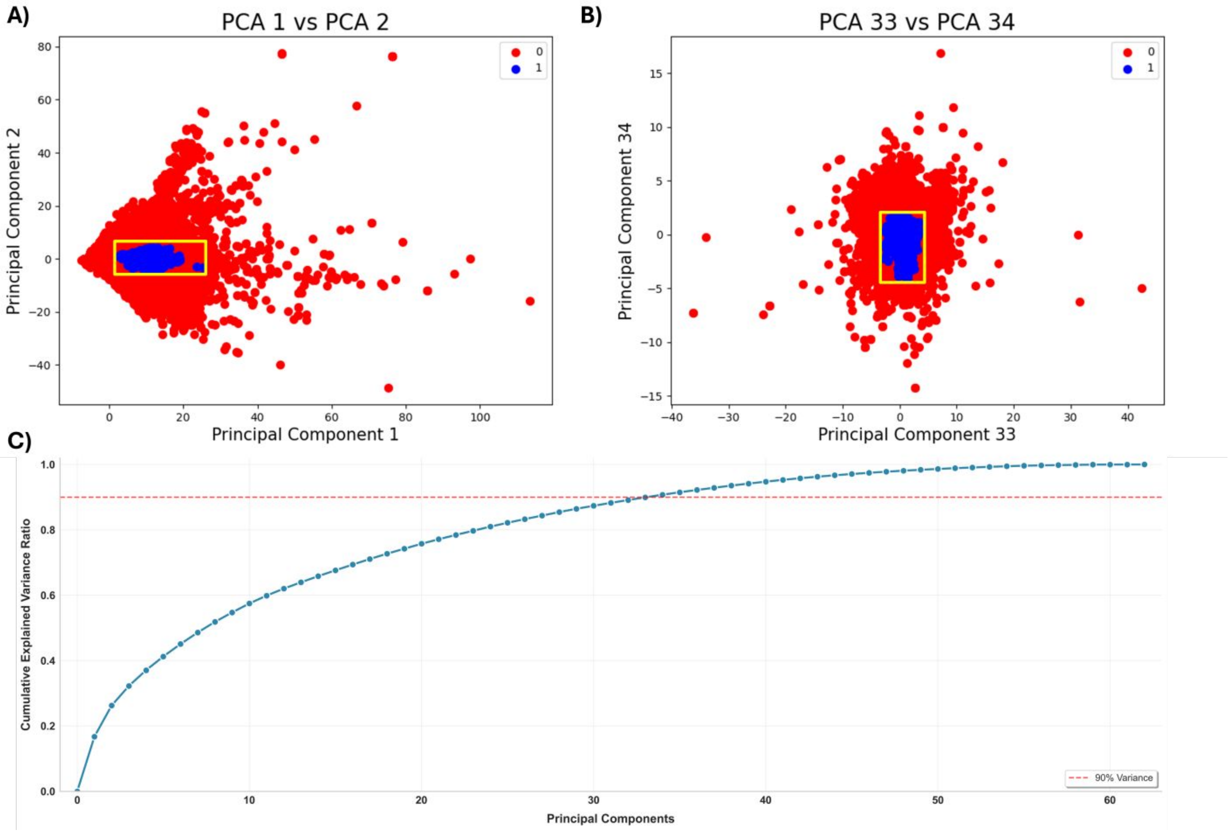

Figure S35. Scatterplots of the two highest (A) and two lowest (B) principal components (PCs). Red points represent molecules extracted from CSD entries; blue points represent PROTACs from PROTAC-DB. Yellow rectangles indicate the PC ranges spanned by the PROTACs, defined by their minimum and maximum values. For each PC, CSD molecules falling within this range were identified, and only those present in all PC-specific lists were retained, yielding 18,559 entries. (C) Explained variance ratio as a function of the number of PCs. The selected 34 PCs account for 90.7% of the total variance.

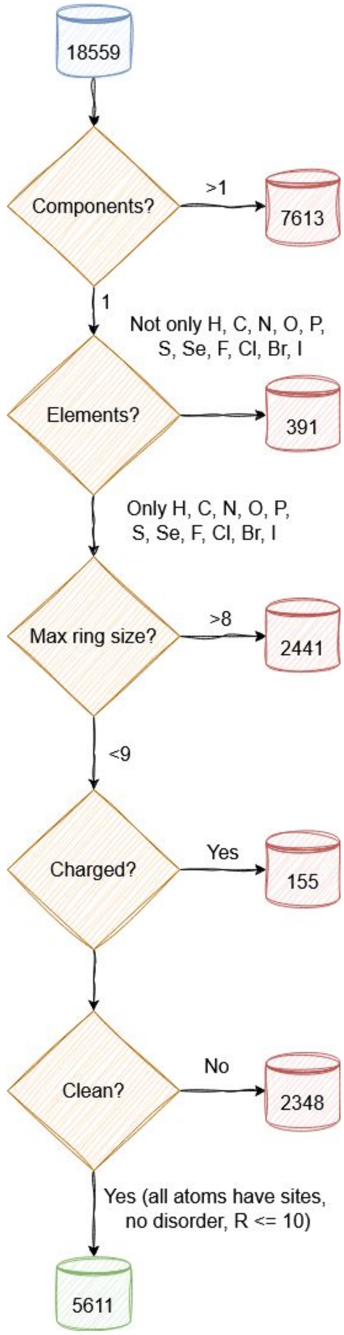

Figure S36. Flowchart of the CSD Python API-based filtering pipeline, which reduced the number of molecules from 18,559 (blue bin) to 5,611 (green bin). Filtering steps retained only molecules that were single-component, non-macrocyclic, uncharged, composed of permitted elements (H, C,

N, O, P, S, Se, F, Cl, Br, I), and of high crystallographic quality. Red bins indicate the number of molecules excluded at each step.

After visual inspection of the 5,611 filtered entries, 116 PROTAC-like molecules were selected for the PCG secondary validation set:

ABAQED, ACEZAK, ACEZEP, ACOQIS, ACUHOV, ACUHUB, AFIFIF, AHIHAC, AKETAO, AKOYEE, ANUHEX, AWEZIO, AXEFUG, AYEMOJ, BALGEA, BAVMUK, BAWWOM, BCGPLG10, BELMOX, BEMZEZ, BEWYIN, BEYQUR, BEYVUY, BICYOF, BOTYIU, BUBFUZ, BUTCIE, CAGYAK, CALFOK, CAWJUJ, COCHOT, COCZIF, DADQAA01, DAKNIN, DAZPAY, DICJIJ, DUPBEY, EHIZON, EMIPEX, FUGHAQ, FURGOR, FUWHEL, GASCIO, GAXKIC, GEJRIY, HEDLOU, HEYQOS, HOLXEL, ILEZEG, IMUGAB, IMUROX, ITIDAT, IXITOZ, LENJUK, LIGKAN, MAHXIC, MAHXOI, MOWNER01, MUDKUT, NATPEG, NEZWAR, OBEBIG, OFINUP, OGEZUX, OGIWAE, OHUMOV, OTUYEK, OXEYEY, PAMHOA, PATCES, PAYLOT, PEZFUW, PIFXEI, POVVII, QAMROL, QASGIC, QEXXOK, QEXXUQ, QEZWOI, QOFYER, RAFROG, RAGBAF, RAHZUY, RAKWOQ, RAKXIL, RAWKIL, RENDUL, REVHOQ, RIGPEC, ROCPAB, RODPAD, ROPVUN, RURYUB, SAJVIM, SEFTAA, SENVUE, SERMOT, SIJHEZ, SUVGUO, SUVGUO01, TIVXEE, TOKCIG, UVUNEH, VARTEP, WAJYUC, WAMYAO, WASVAN, WIFQUY, WIVBIO, XIBXEM, XOPRAX, YAPMUY, YOWJUS, ZEZXAH, ZILWIC, ZINYOO.

Comparison of linker complexity between PROTACs and PROTAC-like molecules

To assess whether the PROTAC-like molecules selected from the CSD represent a comparable conformational challenge to real PROTACs, we analyzed and compared their linker complexity. Specifically, we examined the distribution of acyclic rotatable bonds in the linker region, a key determinant of conformational flexibility. Additionally, we investigated the relationship between linker flexibility and conformer generation performance by plotting lig_RMSD and conformer rank against the number of acyclic rotatable bonds.

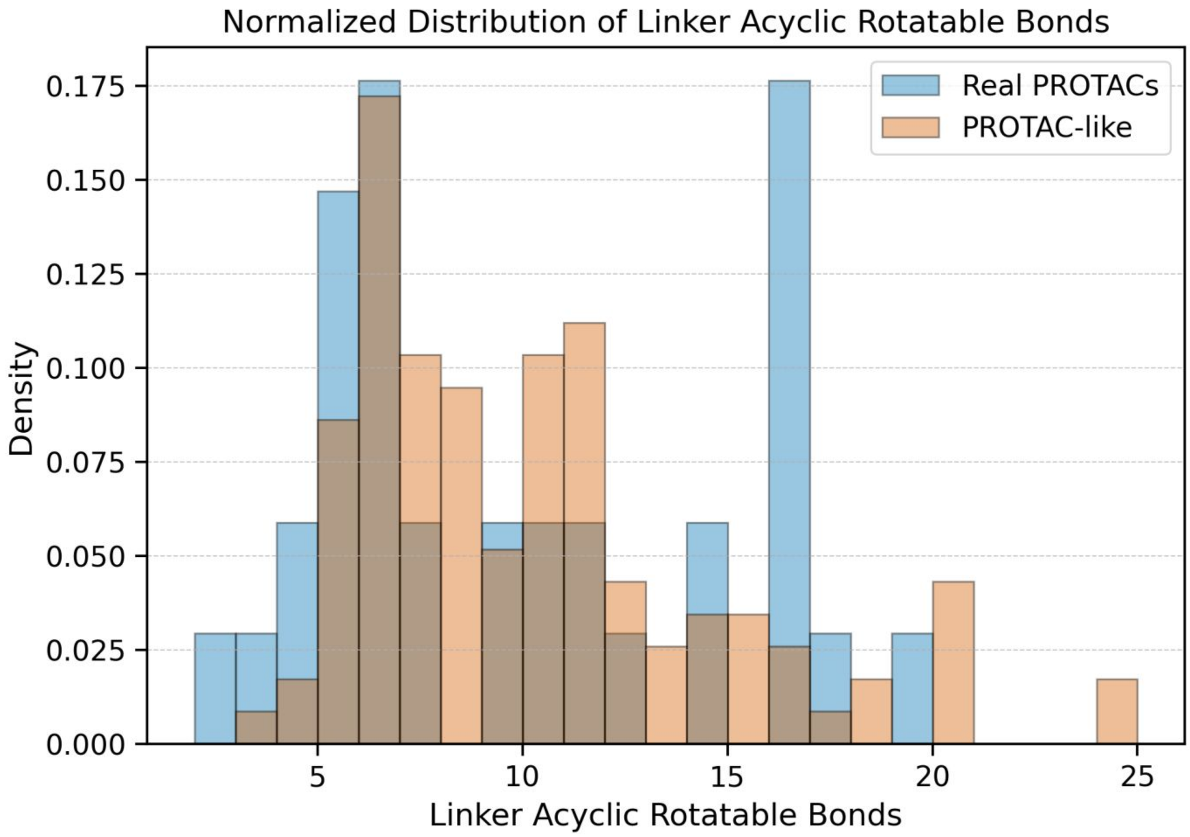

Figure S37. Normalized distribution of acyclic rotatable bonds in the linker region for real PROTACs (blue) and PROTAC-like molecules (orange). Histograms are overlaid using consistent binning and normalized density to account for differences in dataset size. The distributions show similar means and ranges, supporting the use of PROTAC-like molecules as a proxy for evaluating conformational sampling performance.

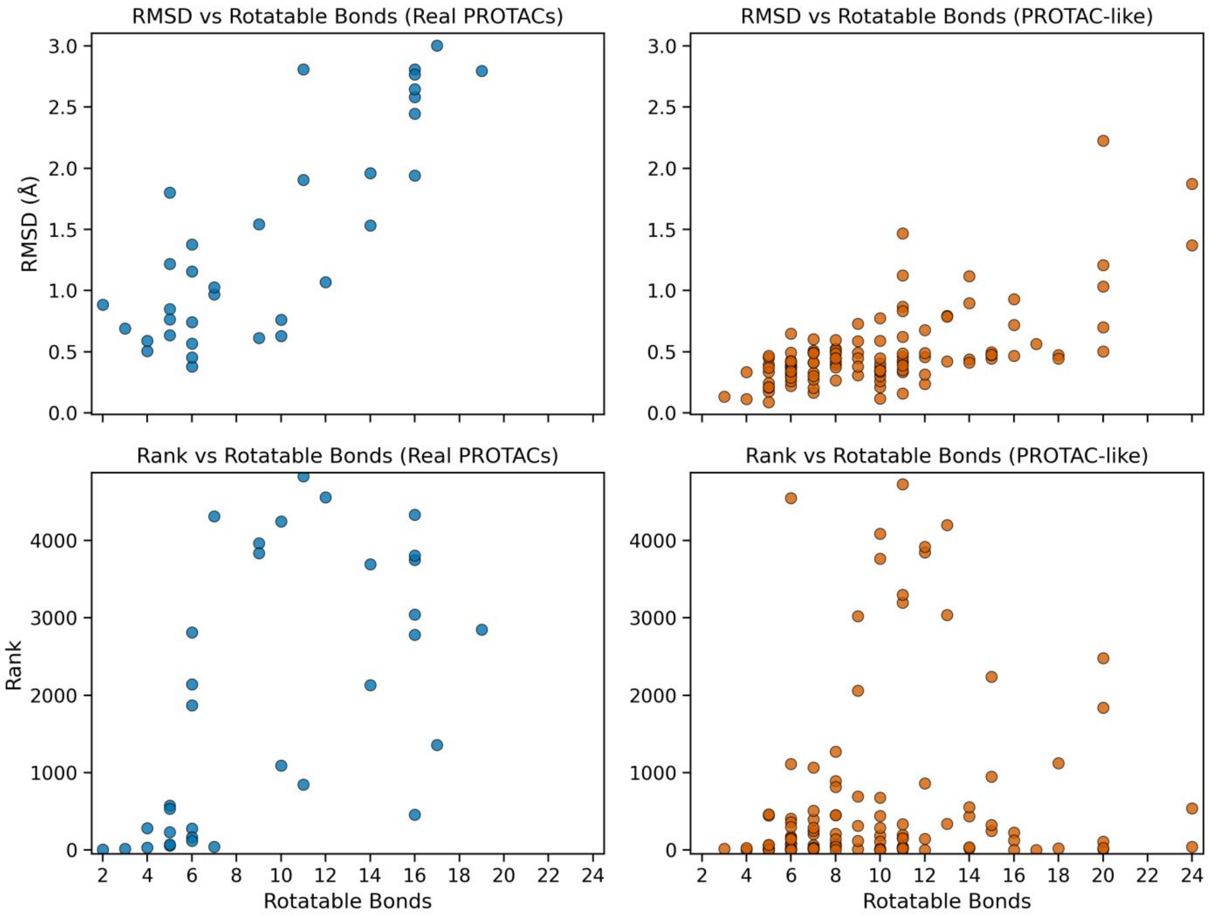

Figure S38. Scatterplots showing the relationship between the number of acyclic rotatable bonds in the linker and conformer generation performance. The top row shows lig_RMSD of the closest conformer; the bottom row shows its rank. The left column corresponds to real PROTACs (blue), and the right to PROTAC-like molecules (orange). A general trend of increasing lig_RMSD with linker flexibility is observed, suggesting that higher rotatable bond counts pose greater challenges for conformational accuracy. The relationship with conformer rank is less pronounced.

Lig_RMSD of closest conformers at different conformational sampling extent

Table S2. Lig_RMSD of the closest conformer to each experimental PROTAC ligand structure, reported for different ensemble sizes (number of generated conformers).

	1	10	50	100	250	500	1000	2500	5000
**5T35**	3.48	2.93	2.39	2.39	2.33	2.33	2.17	2.15	1.90
**6BN7**	4.23	2.74	2.54	2.54	2.16	2.16	2.05	2.02	1.96
**6BOY**	5.10	2.32	2.23	2.23	2.13	2.01	1.78	1.53	1.53
**6HAX**	6.84	4.20	1.18	0.85	0.85	0.57	0.57	0.57	0.57
**6HAY**	5.08	1.67	0.77	0.77	0.77	0.77	0.77	0.77	0.63
**6HR2**	6.81	4.14	0.96	0.83	0.38	0.38	0.38	0.38	0.38
**6W7O**	5.45	2.97	2.95	2.94	2.21	2.21	1.52	1.52	1.38
**6W8I**	5.59	4.26	3.36	3.36	3.01	3.01	3.01	3.00	3.00
**6ZHC**	4.83	3.69	3.69	3.69	3.69	3.37	3.20	3.08	2.80
**7JTO**	6.20	5.33	3.12	3.04	2.91	2.74	2.67	2.09	1.94
**7JTP**	4.20	3.04	1.42	0.85	0.85	0.85	0.85	0.85	0.85
**7KHH**	7.27	4.78	3.40	3.40	3.40	2.94	2.81	2.81	2.81
**7PI4**	3.84	0.88	0.88	0.88	0.88	0.88	0.88	0.88	0.88
**7Q2J**	3.46	2.20	1.21	1.21	1.21	1.12	0.61	0.45	0.45
**7S4E**	2.19	2.19	1.25	1.25	1.25	1.24	1.24	1.20	0.97
**7TVA**	5.32	2.32	2.10	1.80	1.80	1.80	1.80	1.80	1.80
**7Z6L**	3.09	1.37	1.37	0.91	0.91	0.74	0.64	0.64	0.64
**7Z76**	1.89	1.18	0.93	0.82	0.82	0.82	0.82	0.76	0.76
**7Z77**	2.80	1.16	0.51	0.51	0.51	0.51	0.51	0.51	0.51
**7ZNT**	4.27	3.45	1.26	0.84	0.84	0.84	0.84	0.84	0.61
**8BB2**	5.25	3.43	3.43	2.76	2.76	2.67	2.67	2.67	2.58

**8BB3**	4.68	3.69	3.15	2.91	2.91	2.45	2.45	2.45	2.45
**8BB4**	3.45	2.53	1.83	1.83	1.22	1.22	1.22	1.22	1.22
**8BB5**	3.69	2.06	1.18	0.92	0.92	0.85	0.76	0.76	0.76
**8BDS**	6.68	5.27	3.40	3.40	3.24	3.19	3.01	2.88	2.81
**8BDT**	5.73	4.28	3.93	3.93	3.66	3.55	3.30	3.08	2.65
**8BDX**	5.72	3.94	3.94	3.74	3.42	3.42	3.25	2.87	2.77
**8DSO**	4.39	3.86	1.03	1.03	1.03	1.03	1.03	1.03	1.03
**8G1P**	4.77	2.75	2.41	2.41	1.95	1.95	1.95	1.64	1.54
**8G1Q**	7.06	2.84	1.02	1.02	0.74	0.74	0.74	0.74	0.74

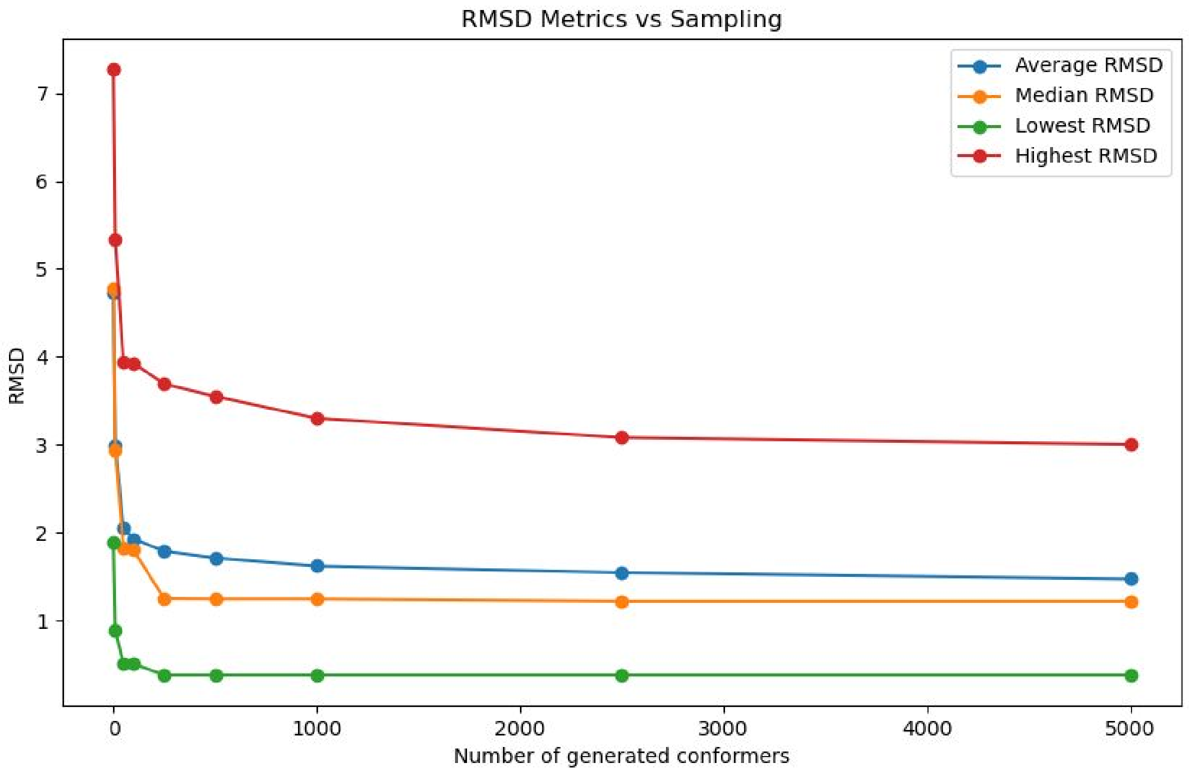

Figure S39. Decrease of lig_RMSD with increasing conformational sampling (number of generated conformers). Blue, orange, green, and red lines represent the average, median, lowest, and highest lig_RMSD across all PROTAC cases, respectively.

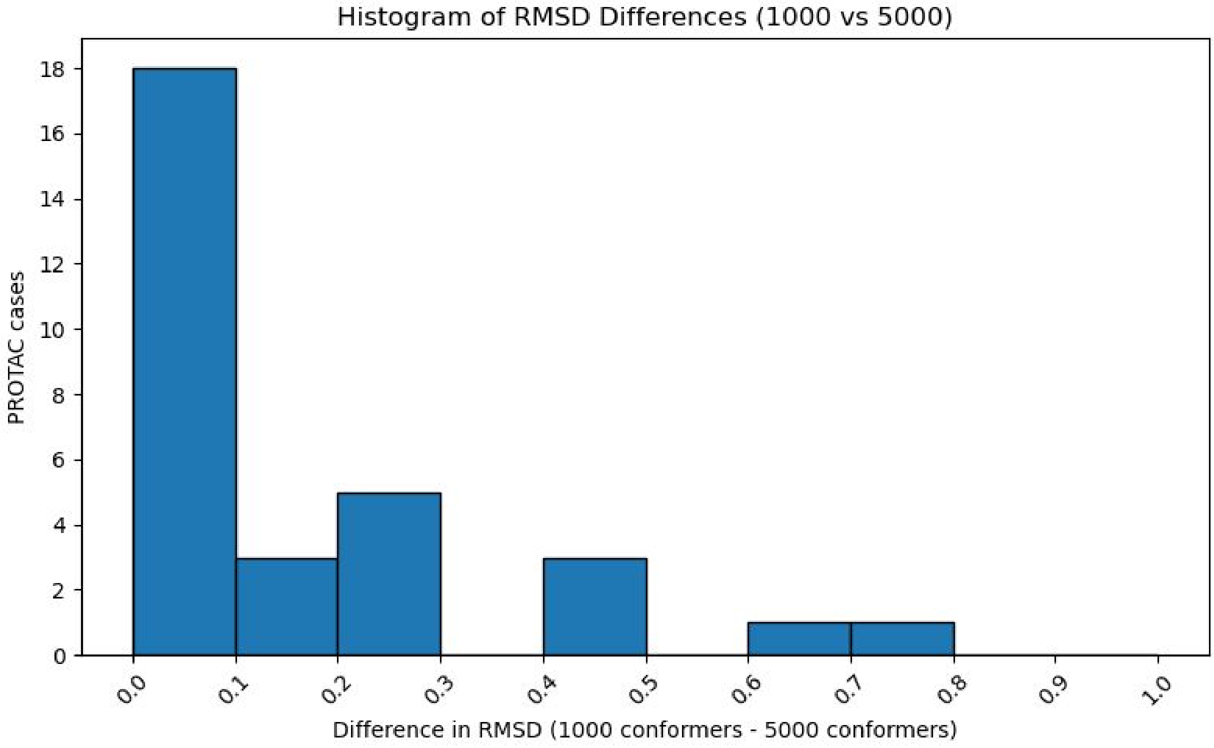

Figure S40. Histogram showing the distribution of lig_RMSD differences between ensembles of 1000 and 5000 conformers. A difference greater than 0.5 Å was observed in only two cases, indicating that reduced sampling may still yield comparable accuracy in most instances.

Comparison between experimental structures and closest conformers energies

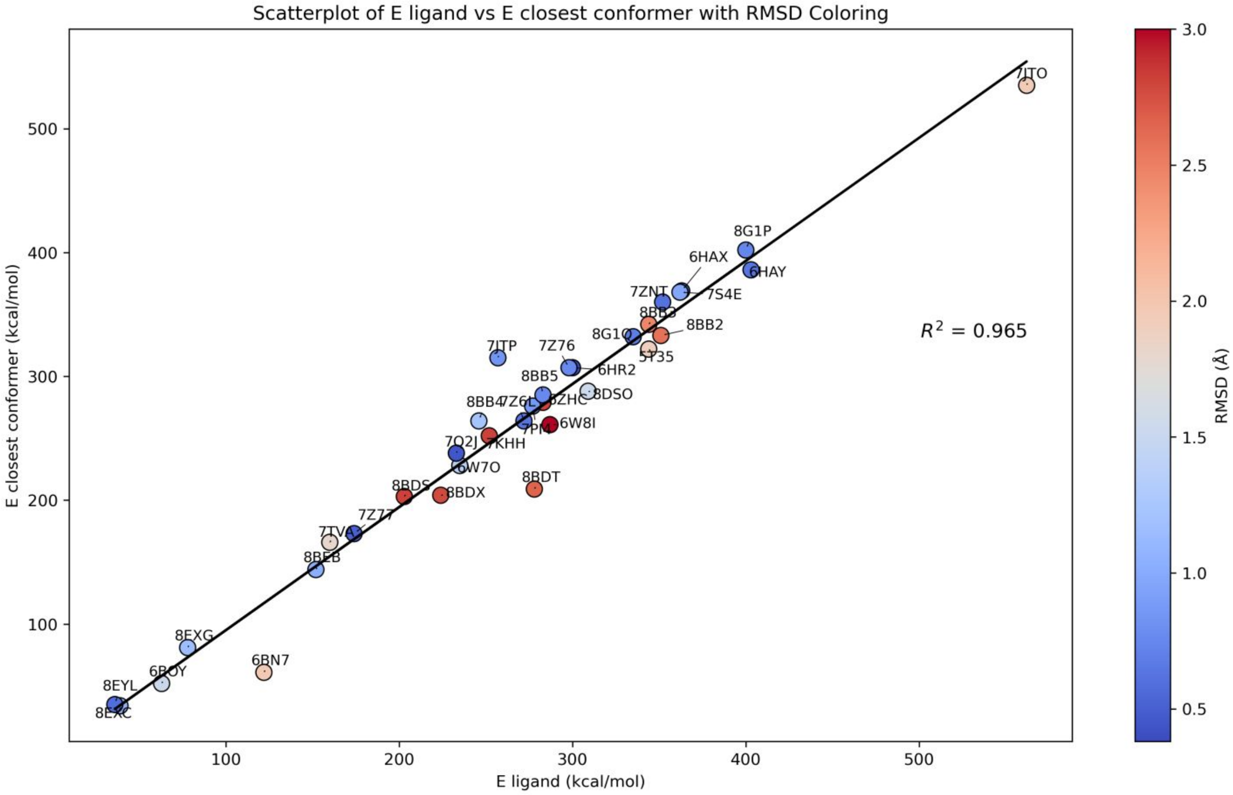

Figure S41. Scatterplot comparing MMFF94 force field energies of the experimentally observed PROTAC ligand conformations and their closest conformers generated by the PCG. Points are labeled by PDB code and colored according to their lig_RMSD, as indicated by the color scale. A trendline is shown as a solid black line. The R^2 value indicates strong linear correlation between experimental and modeled energies.

Table S3. MMFF94 force field energies calculated for the experimentally observed PROTAC ligand conformations and their closest conformers among the 5000 generated by the PCG. The corresponding lig_RMSD values are also reported.

	Lig_RMSD (Å)	E (kcal/mol) PROTAC	E (kcal/mol) closest conformer
**5T35**	1.90	344	322
**6BN7**	1.96	122	61
**6BOY**	1.53	63	52
**6HAX**	0.57	363	369
**6HAY**	0.63	403	386
**6HR2**	0.38	300	307
**6W7O**	1.38	235	228
**6W8I**	3.00	287	261
**6ZHC**	2.80	283	279
**7JTO**	1.94	562	535
**7JTP**	0.85	257	315
**7KHH**	2.81	252	252
**7PI4**	0.88	277	276
**7Q2J**	0.45	233	238
**7S4E**	0.97	362	368
**7TVA**	1.80	160	166
**7Z6L**	0.64	272	264
**7Z76**	0.76	298	307
**7Z77**	0.51	174	173
**7ZNT**	0.61	352	360

**8BB2**	2.58	351	333
**8BB3**	2.45	344	342
**8BB4**	1.22	246	264
**8BB5**	0.76	283	285
**8BDS**	2.81	203	203
**8BDT**	2.65	278	209
**8BDX**	2.77	224	204
**8BEB**	1.03	152	144
**8DSO**	1.54	309	288
**8EXC**	1.07	39	34
**8EXG**	1.16	78	81
**8EYL**	0.59	36	35
**8G1P**	0.74	400	402
**8G1Q**	0.69	335	332

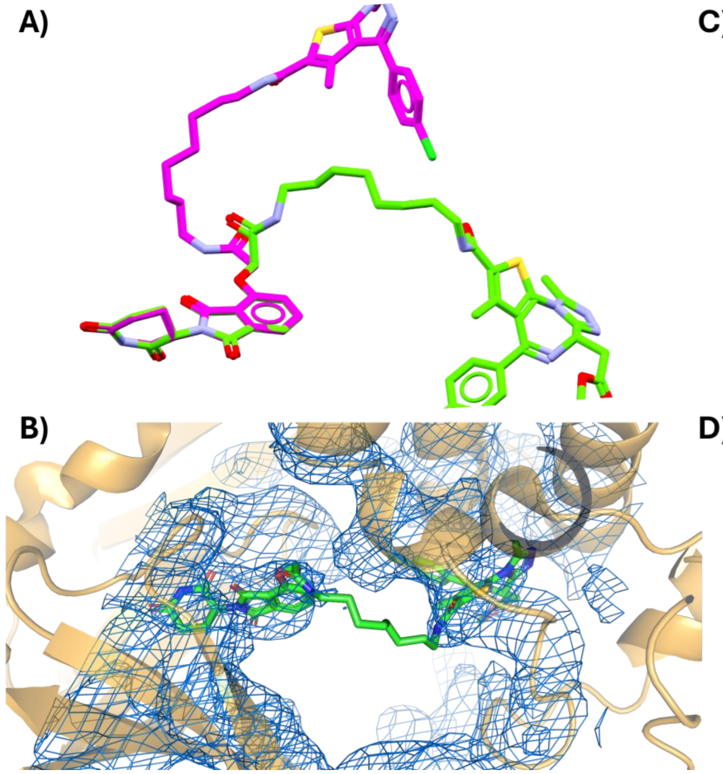

C)

C24 C23 C22 C21	Not unusual (enough hits)	10000	-75.123
C22 C23 C24 C25	Not unusual (enough hits)	10000	164.727
C24 C25 N4 C26	Not unusual (enough hits)	3765	-136.140
C20 C19 C18 N3	Unusual (enough hits)	4225	100.883
C21 C20 C19 C18	Unusual (enough hits)	10000	-142.282
C22 C21 C20 C19	Unusual (enough hits)	10000	-87.711
C23 C22 C21 C20	Unusual (enough hits)	10000	-128.111
C23 C24 C25 N4	Unusual (enough hits)	4225	-104.239
O2 C27 C26 N4	Unusual (enough hits)	1386	104.429
O7 C26 C27 O2	Unusual (enough hits)	1627	-75.619
O8 C17 N3 C18	Unusual (enough hits)	1755	-30.796
C19 C18 N3 C17	Unusual (enough hits)	3765	57.443
C27 C26 N4 C25	Unusual (enough hits)	799	151.414
O7 C26 N4 C25	Unusual (enough hits)	2858	-28.536

D)

C20 C19 C18 N3	Not unusual (enough hits)	4225	-179.998
C21 C20 C19 C18	Not unusual (enough hits)	10000	67.002
C22 C21 C20 C19	Not unusual (enough hits)	10000	179.998
C23 C22 C21 C20	Not unusual (enough hits)	10000	-180.000
C24 C23 C22 C21	Not unusual (enough hits)	10000	-67.002
C22 C23 C24 C25	Not unusual (enough hits)	10000	-179.998
C23 C24 C25 N4	Not unusual (enough hits)	4225	-65.001
O2 C27 C26 N4	Not unusual (enough hits)	1386	0.040
O7 C26 C27 O2	Not unusual (enough hits)	1627	179.999
O8 C17 N3 C18	Not unusual (enough hits)	1755	1.029
C19 C18 N3 C17	Not unusual (enough hits)	3765	-178.000
C24 C25 N4 C26	Not unusual (enough hits)	3765	-81.994
C27 C26 N4 C25	Not unusual (enough hits)	799	179.950
O7 C26 N4 C25	Not unusual (enough hits)	2858	-0.007

Figure S42. A) Overlay of the experimental PROTAC ligand (green) and its closest conformer (magenta) for PDB structure 6BN7, aligned on the E3 recruiting group. B) 2Fo-Fc electron density map of the binding site contoured at 0.8σ, showing missing density around the linker. C) Mogul geometry analysis of the experimental linker conformation, highlighting several unusual torsion angles. D) Mogul analysis of the PCG-generated closest conformer, showing no unusual torsions. The last two columns in panels C and D report the number of observations and the query value, respectively.

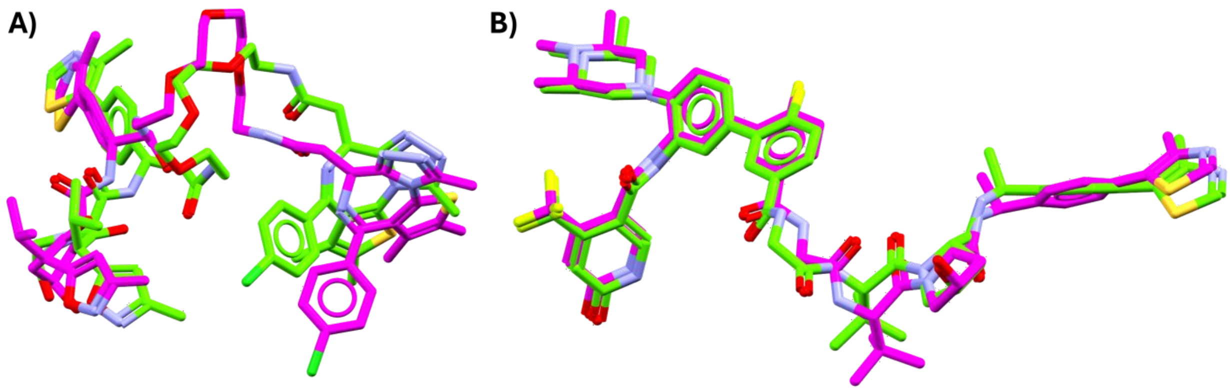

Figure S43. A) Overlay of the experimental PROTAC ligand (green) and its closest conformer (magenta) for PDB structure 8BDT. B) Overlay of the experimental PROTAC ligand (green) and its closest conformer (magenta) for PDB structure 7JTP.

Surface-score vs clash-score scatterplots for all ternary complex ensembles

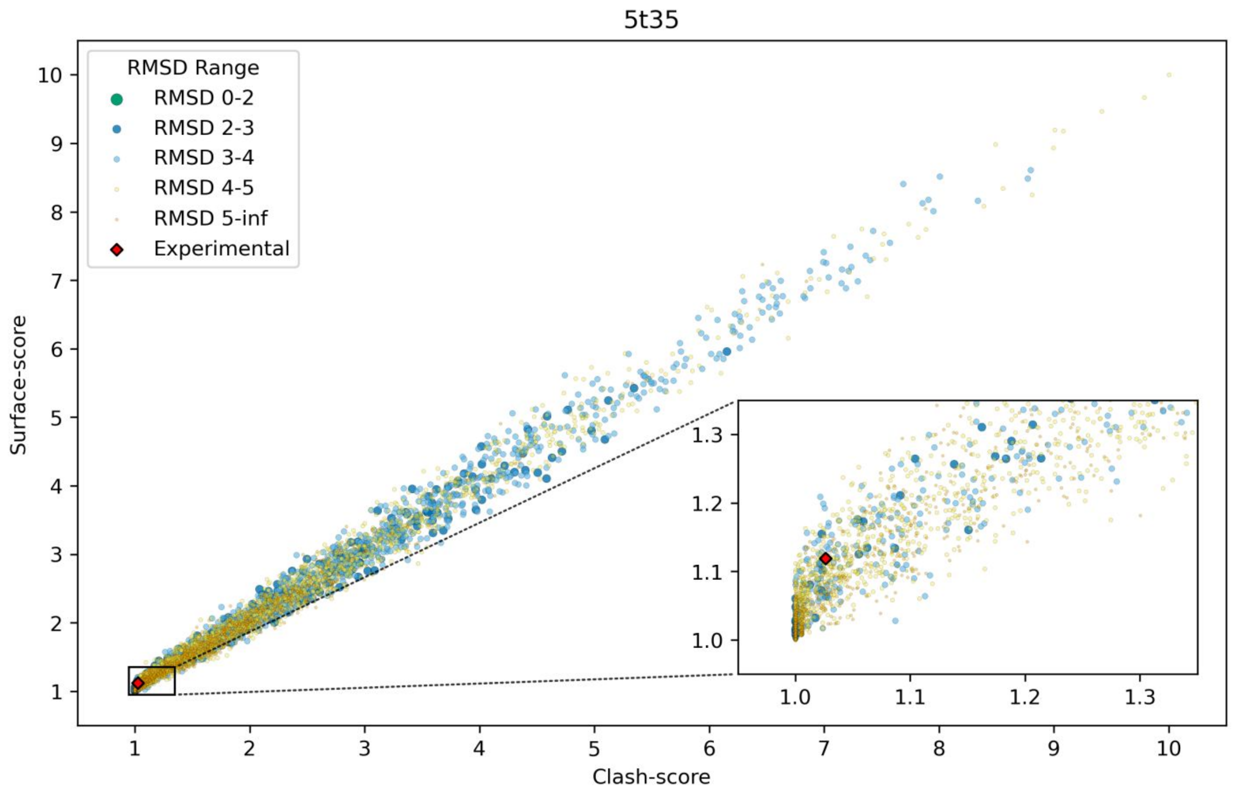

Figure S44. Surface-score vs clash-score scatterplot for the full conformational ensemble of 5T35. Each point represents a model generated by the PCG. Point color, size, and transparency reflect a scale of lig_RMSD (in Å). The red diamond marks the scores of the experimental ternary complex structure for reference.

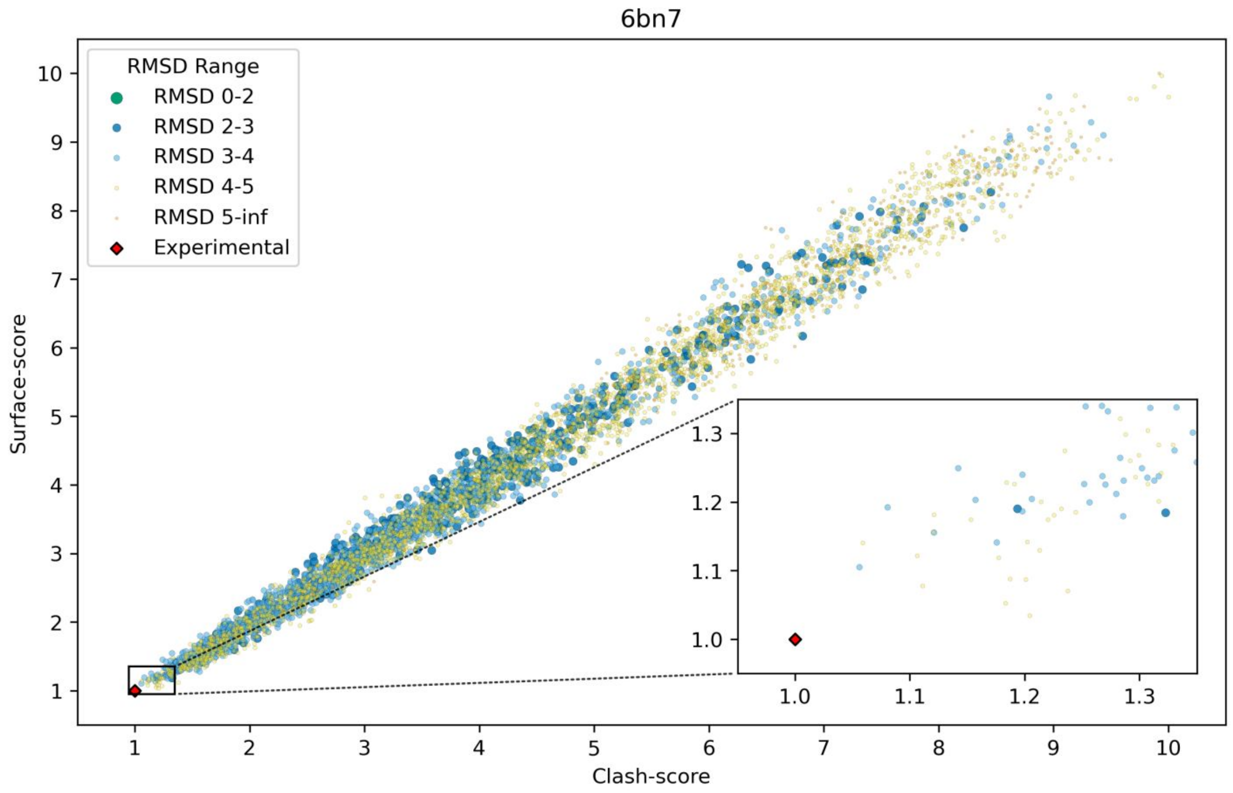

Figure S45. Surface-score vs clash-score scatterplot for the full conformational ensemble of 6BN7. Each point represents a model generated by the PCG. Point color, size, and transparency reflect a scale of lig_RMSD (in Å). The red diamond marks the scores of the experimental ternary complex structure for reference.

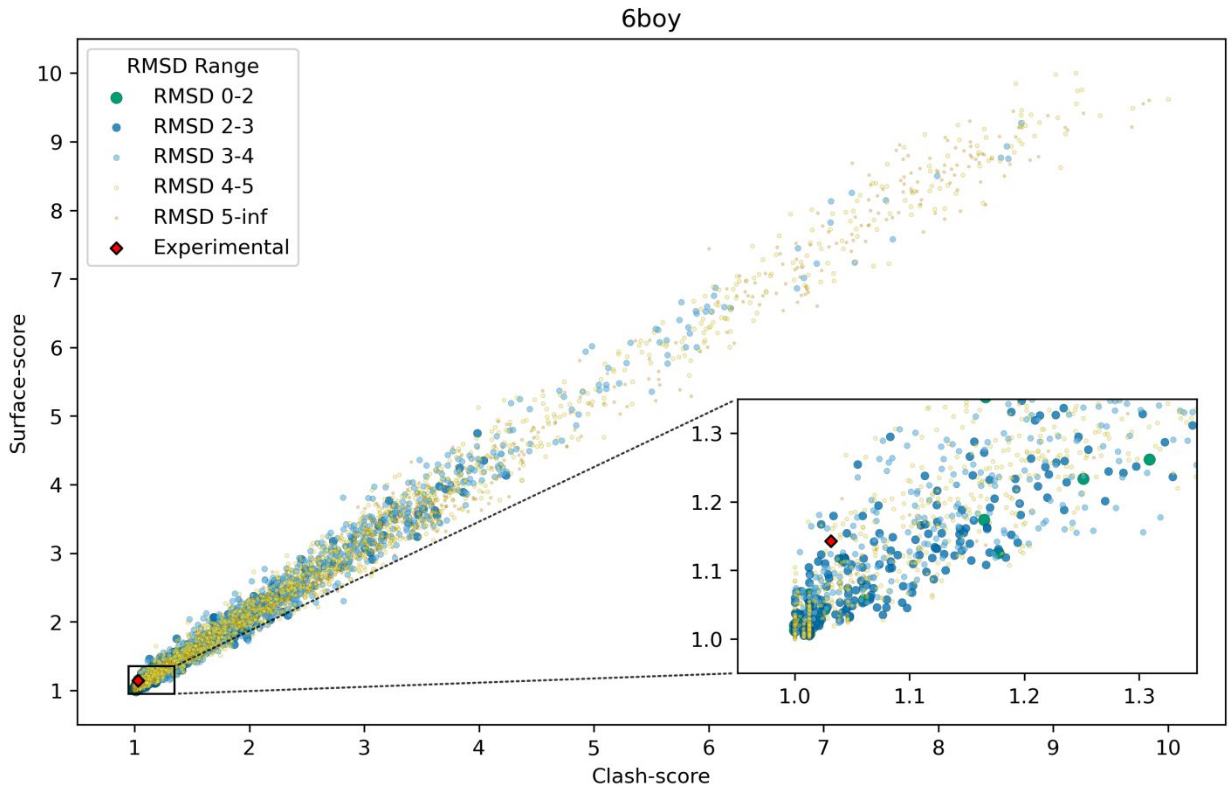

Figure S46. Surface-score vs clash-score scatterplot for the full conformational ensemble of 6BOY. Each point represents a model generated by the PCG. Point color, size, and transparency reflect a scale of lig_RMSD (in Å). The red diamond marks the scores of the experimental ternary complex structure for reference.

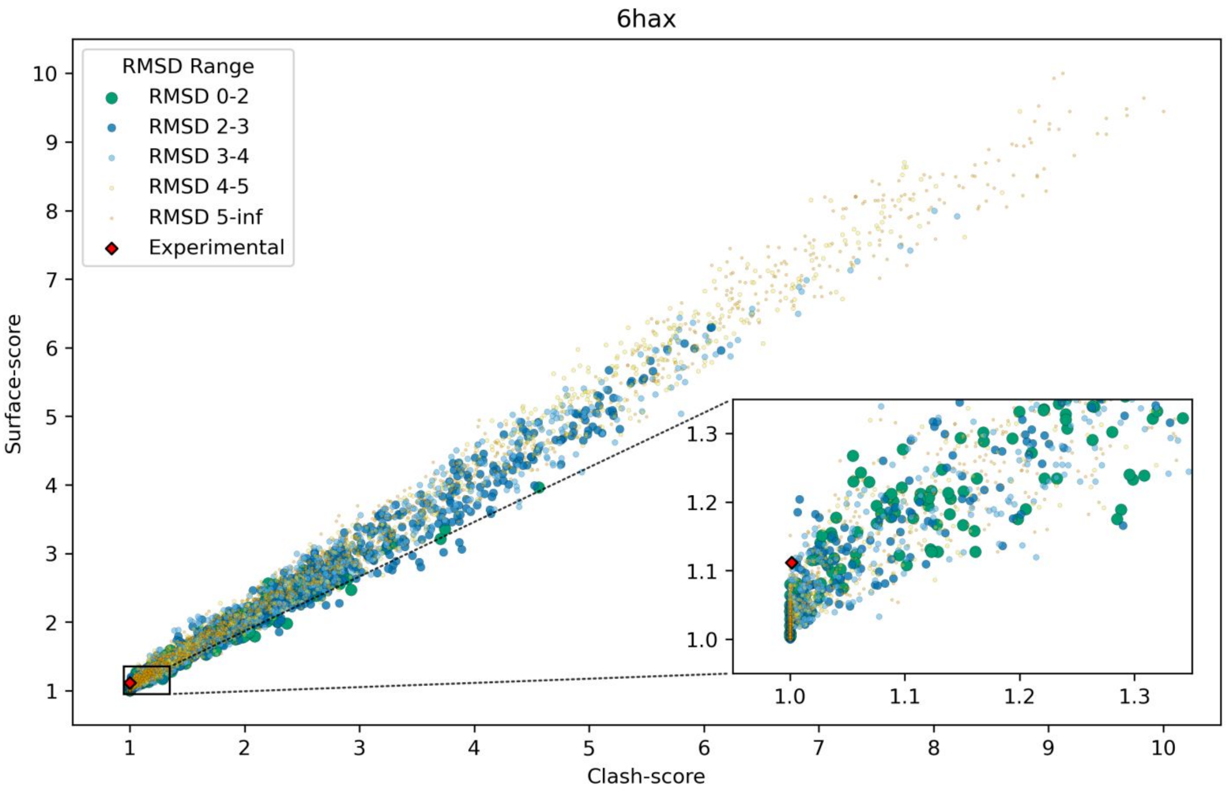

Figure S47. Surface-score vs clash-score scatterplot for the full conformational ensemble of 6HAX. Each point represents a model generated by the PCG. Point color, size, and transparency reflect a scale of lig_RMSD (in Å). The red diamond marks the scores of the experimental ternary complex structure for reference.

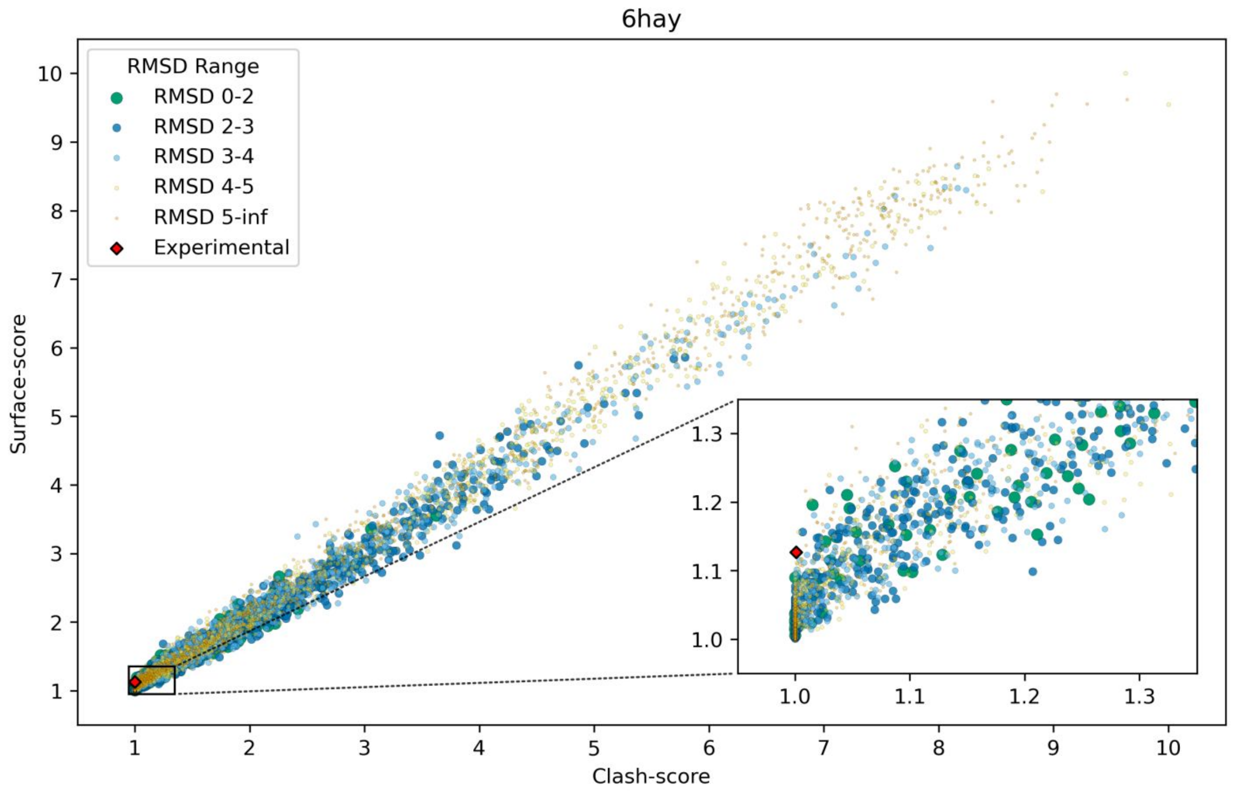

Figure S48. Surface-score vs clash-score scatterplot for the full conformational ensemble of 6HAY. Each point represents a model generated by the PCG. Point color, size, and transparency reflect a scale of lig_RMSD (in Å). The red diamond marks the scores of the experimental ternary complex structure for reference.

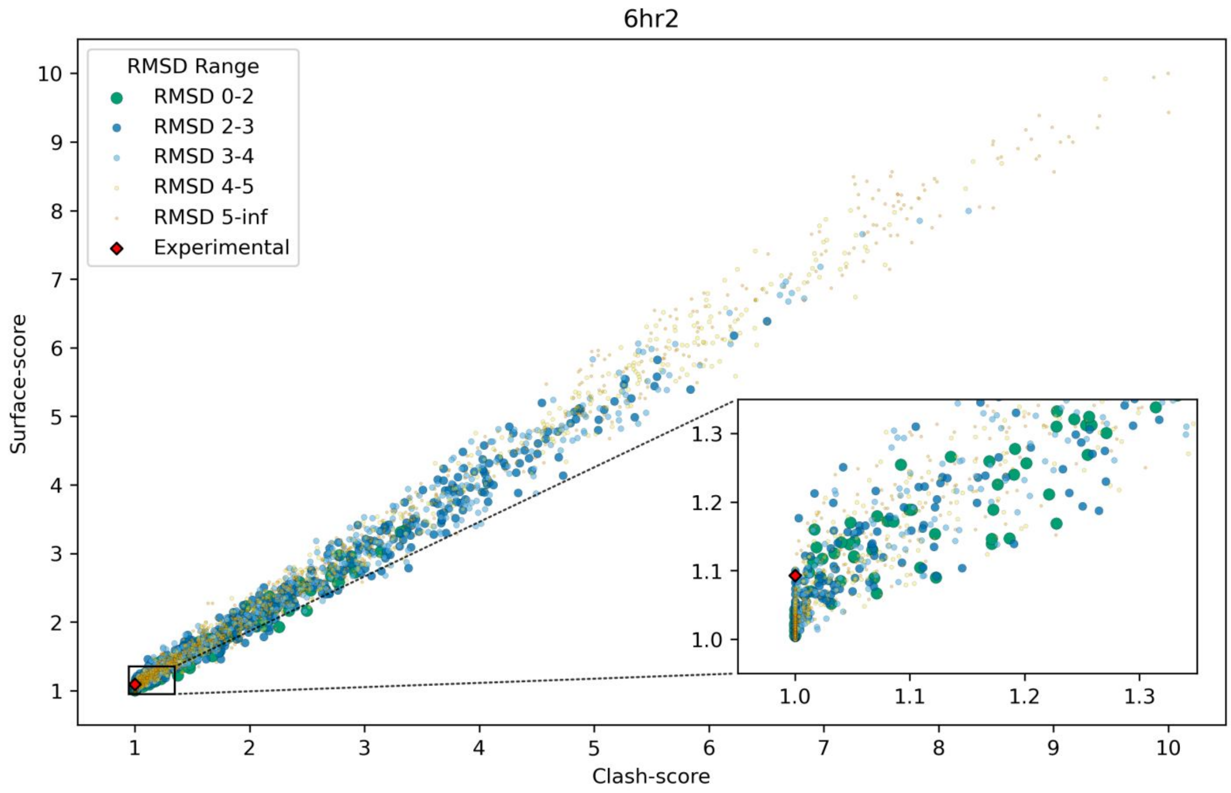

Figure S49. Surface-score vs clash-score scatterplot for the full conformational ensemble of 6HR2. Each point represents a model generated by the PCG. Point color, size, and transparency reflect a scale of lig_RMSD (in Å). The red diamond marks the scores of the experimental ternary complex structure for reference.

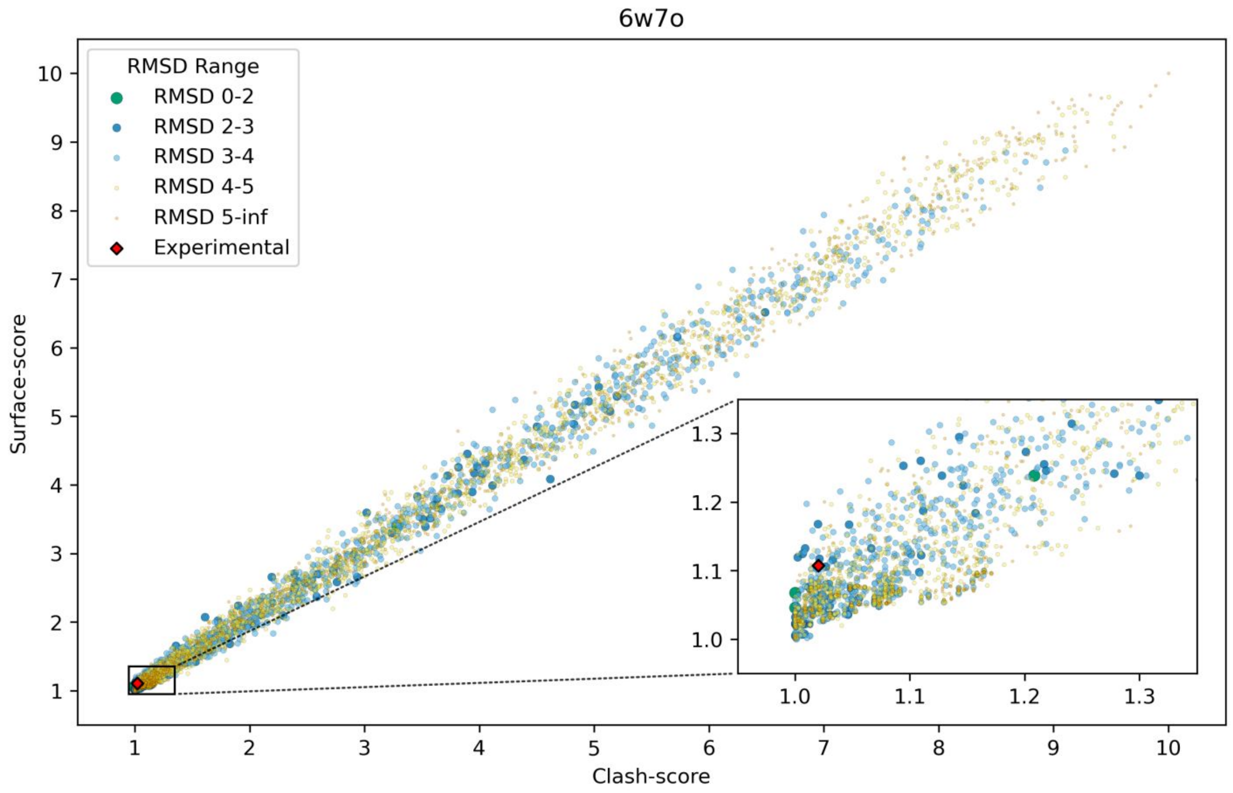

Figure S50. Surface-score vs clash-score scatterplot for the full conformational ensemble of 6W7O. Each point represents a model generated by the PCG. Point color, size, and transparency reflect a scale of lig_RMSD (in Å). The red diamond marks the scores of the experimental ternary complex structure for reference.

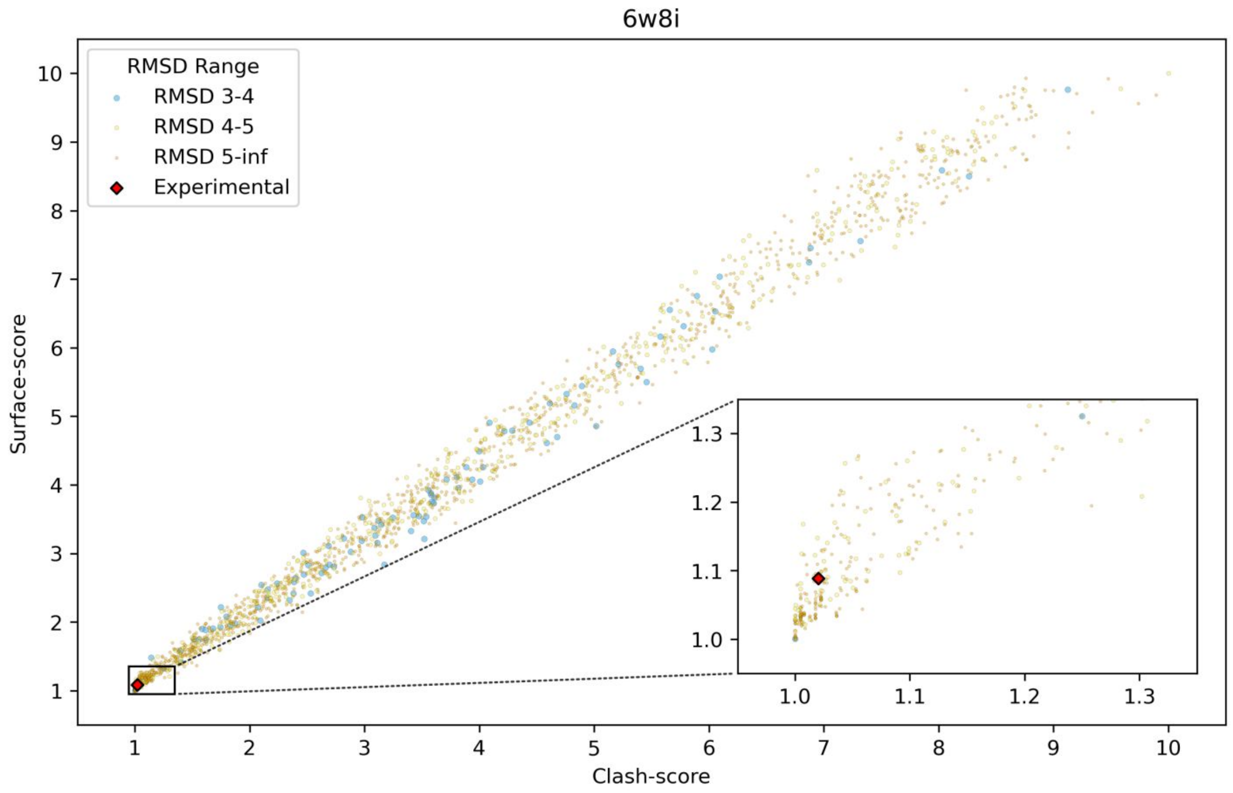

Figure S51. Surface-score vs clash-score scatterplot for the full conformational ensemble of 6W8I. Each point represents a model generated by the PCG. Point color, size, and transparency reflect a scale of lig_RMSD (in Å). The red diamond marks the scores of the experimental ternary complex structure for reference.

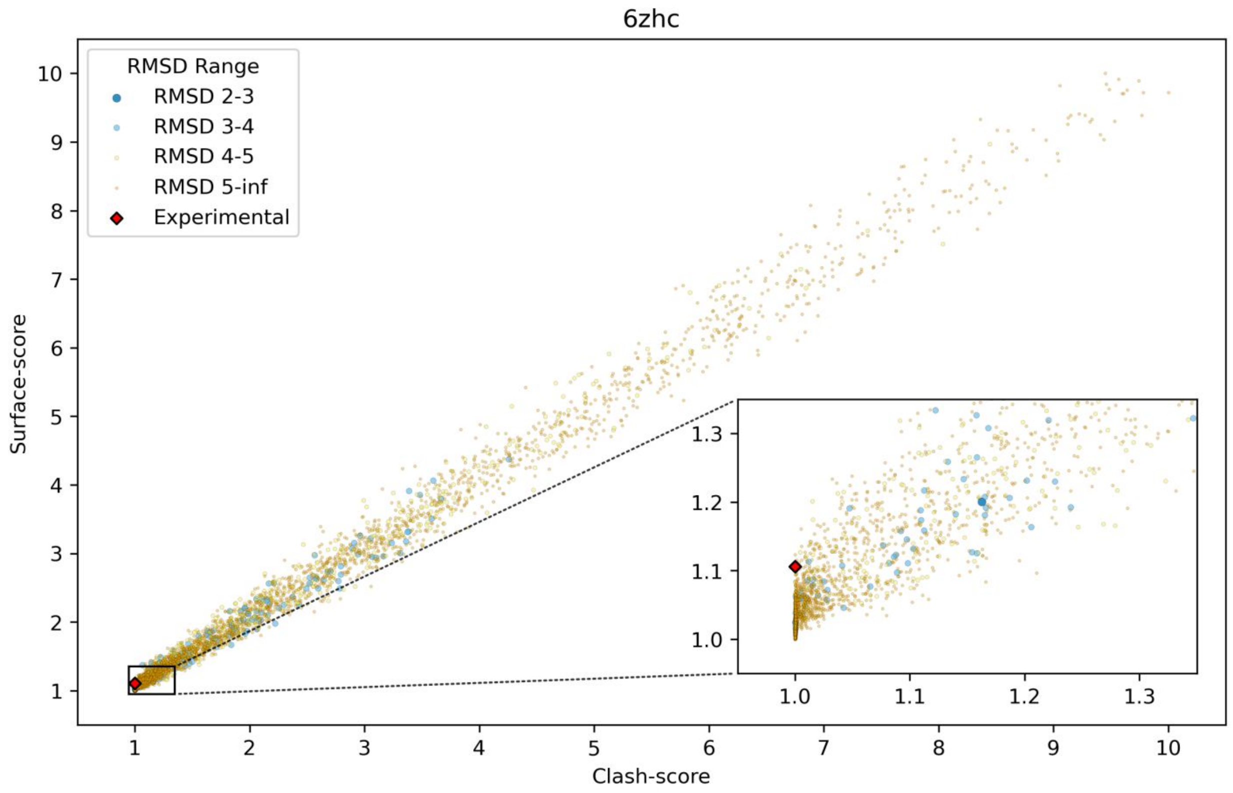

Figure S52. Surface-score vs clash-score scatterplot for the full conformational ensemble of 6ZHC. Each point represents a model generated by the PCG. Point color, size, and transparency reflect a scale of lig_RMSD (in Å). The red diamond marks the scores of the experimental ternary complex structure for reference.

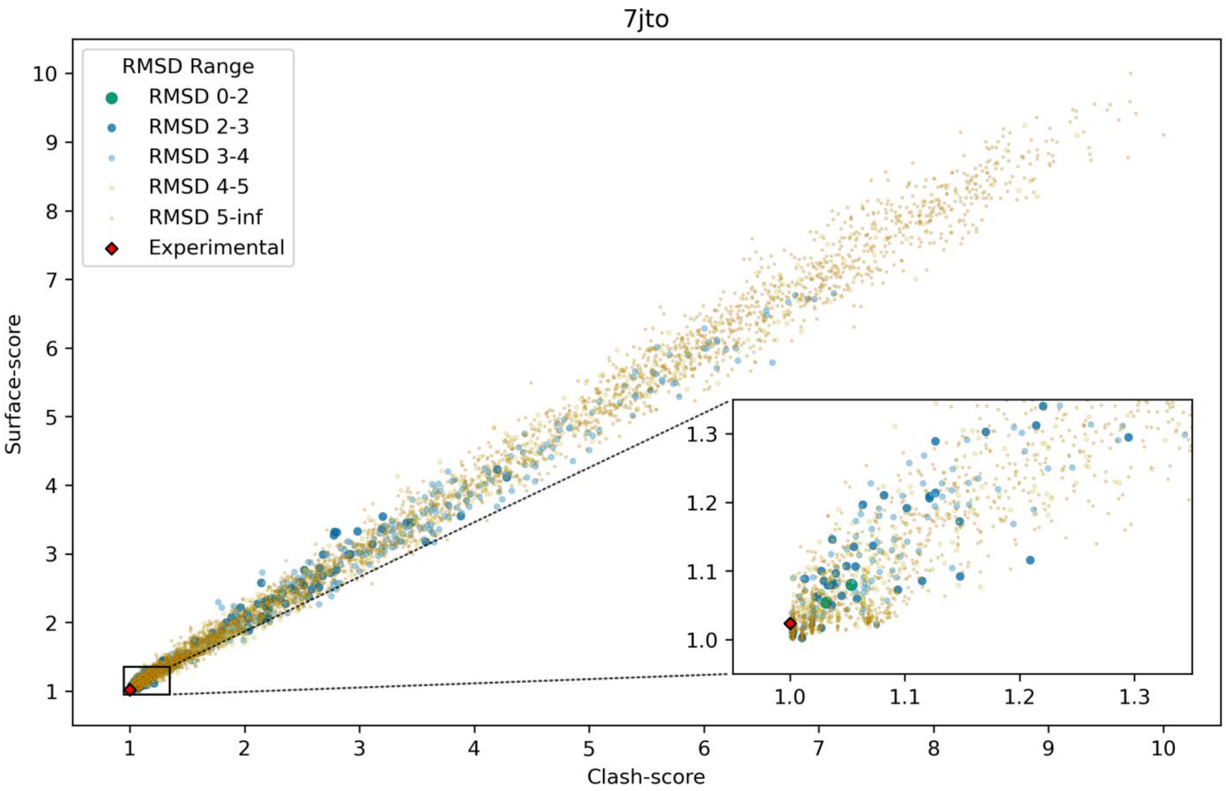

Figure S53. Surface-score vs clash-score scatterplot for the full conformational ensemble of 7JTO. Each point represents a model generated by the PCG. Point color, size, and transparency reflect a scale of lig_RMSD (in Å). The red diamond marks the scores of the experimental ternary complex structure for reference.

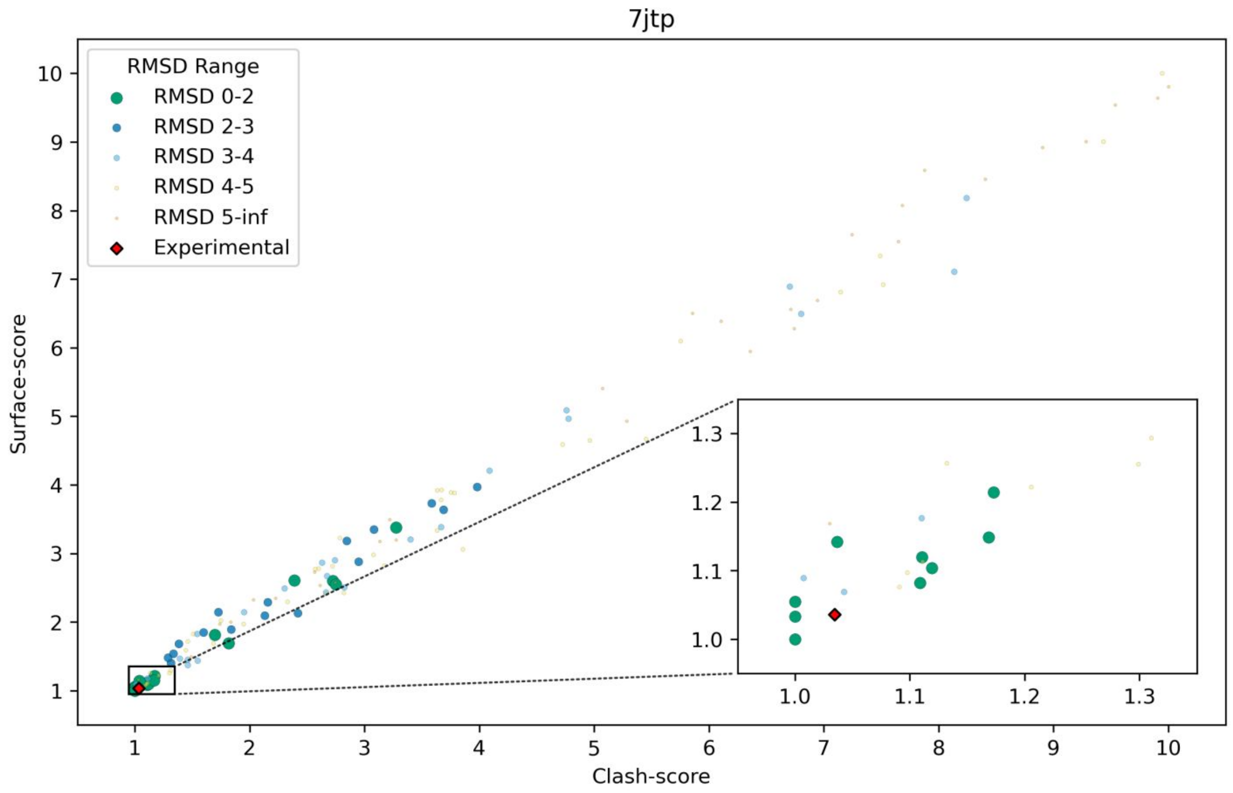

Figure S54. Surface-score vs clash-score scatterplot for the full conformational ensemble of 7JTP. Each point represents a model generated by the PCG. Point color, size, and transparency reflect a scale of lig_RMSD (in Å). The red diamond marks the scores of the experimental ternary complex structure for reference.

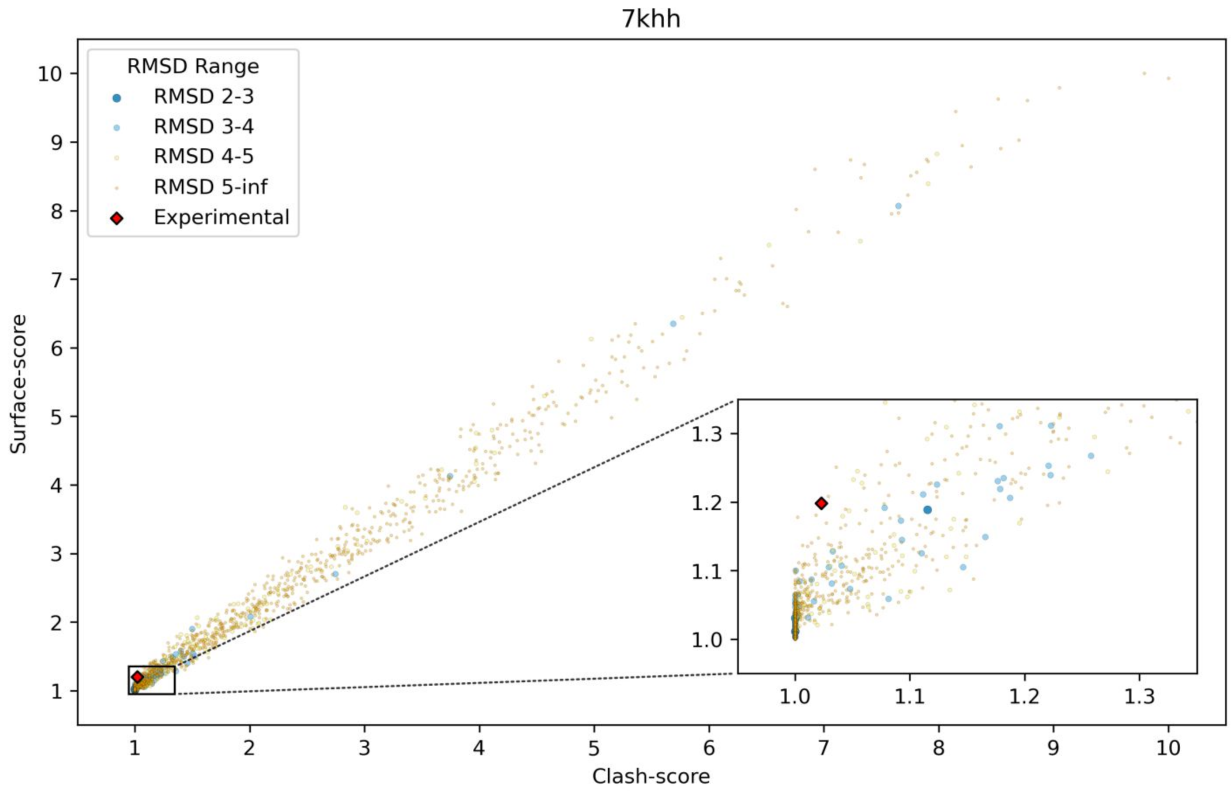

Figure S55. Surface-score vs clash-score scatterplot for the full conformational ensemble of 7KHH. Each point represents a model generated by the PCG. Point color, size, and transparency reflect a scale of lig_RMSD (in Å). The red diamond marks the scores of the experimental ternary complex structure for reference.

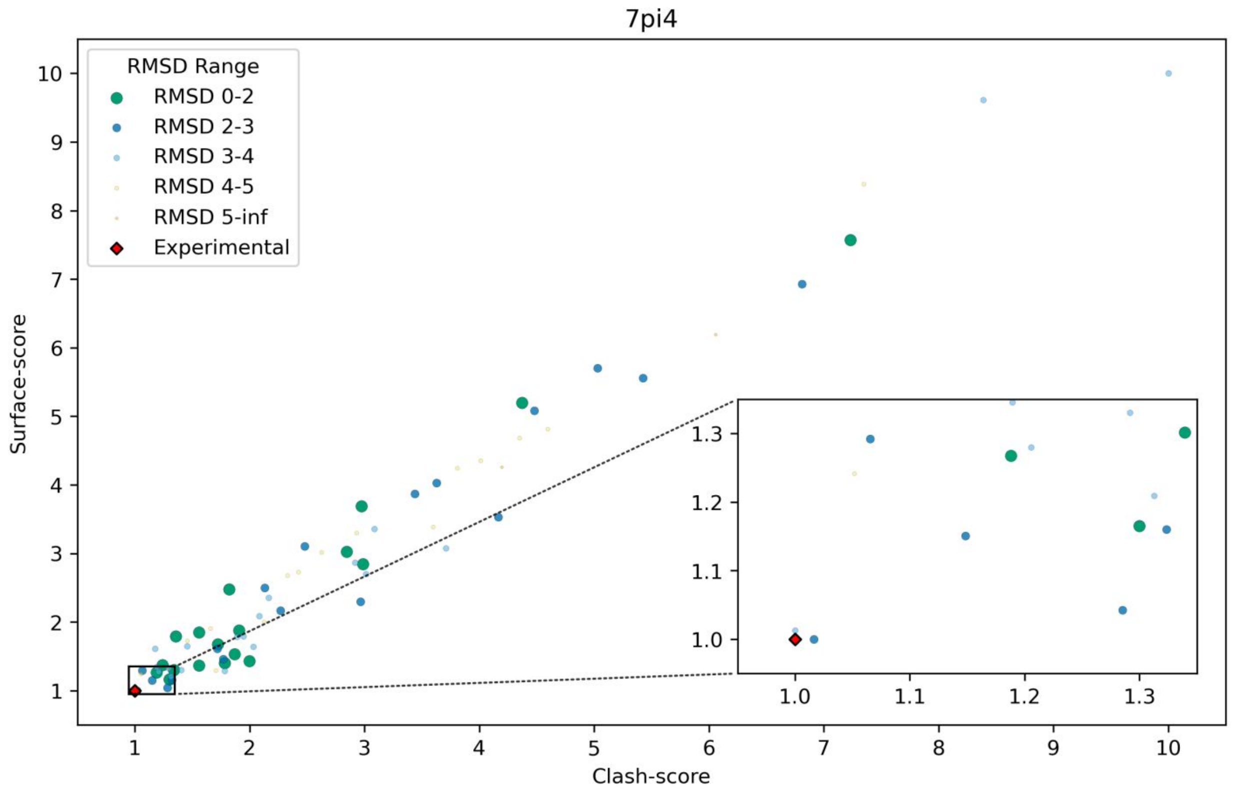

Figure S56. Surface-score vs clash-score scatterplot for the full conformational ensemble of 7PI4. Each point represents a model generated by the PCG. Point color, size, and transparency reflect a scale of lig_RMSD (in Å). The red diamond marks the scores of the experimental ternary complex structure for reference.

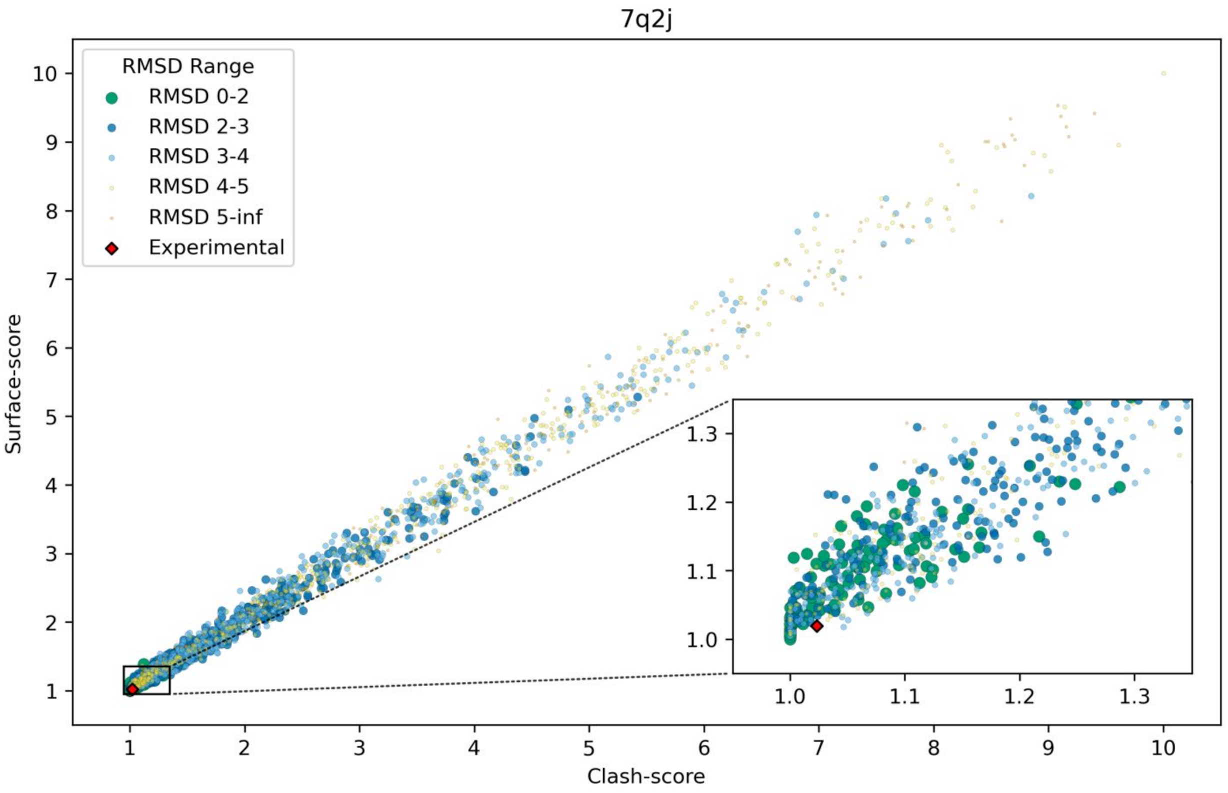

Figure S57. Surface-score vs clash-score scatterplot for the full conformational ensemble of 7Q2J. Each point represents a model generated by the PCG. Point color, size, and transparency reflect a scale of lig_RMSD (in Å). The red diamond marks the scores of the experimental ternary complex structure for reference.

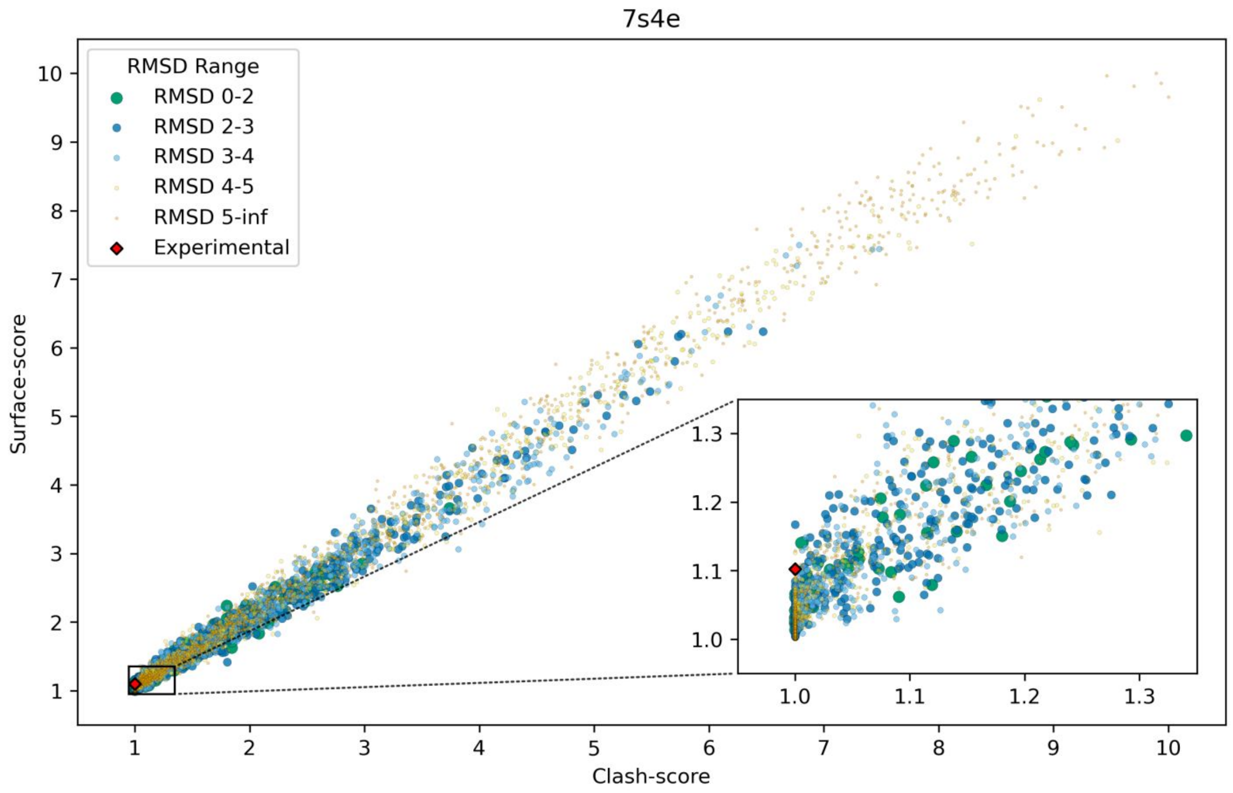

Figure S58. Surface-score vs clash-score scatterplot for the full conformational ensemble of 7S4E. Each point represents a model generated by the PCG. Point color, size, and transparency reflect a scale of lig_RMSD (in Å). The red diamond marks the scores of the experimental ternary complex structure for reference.

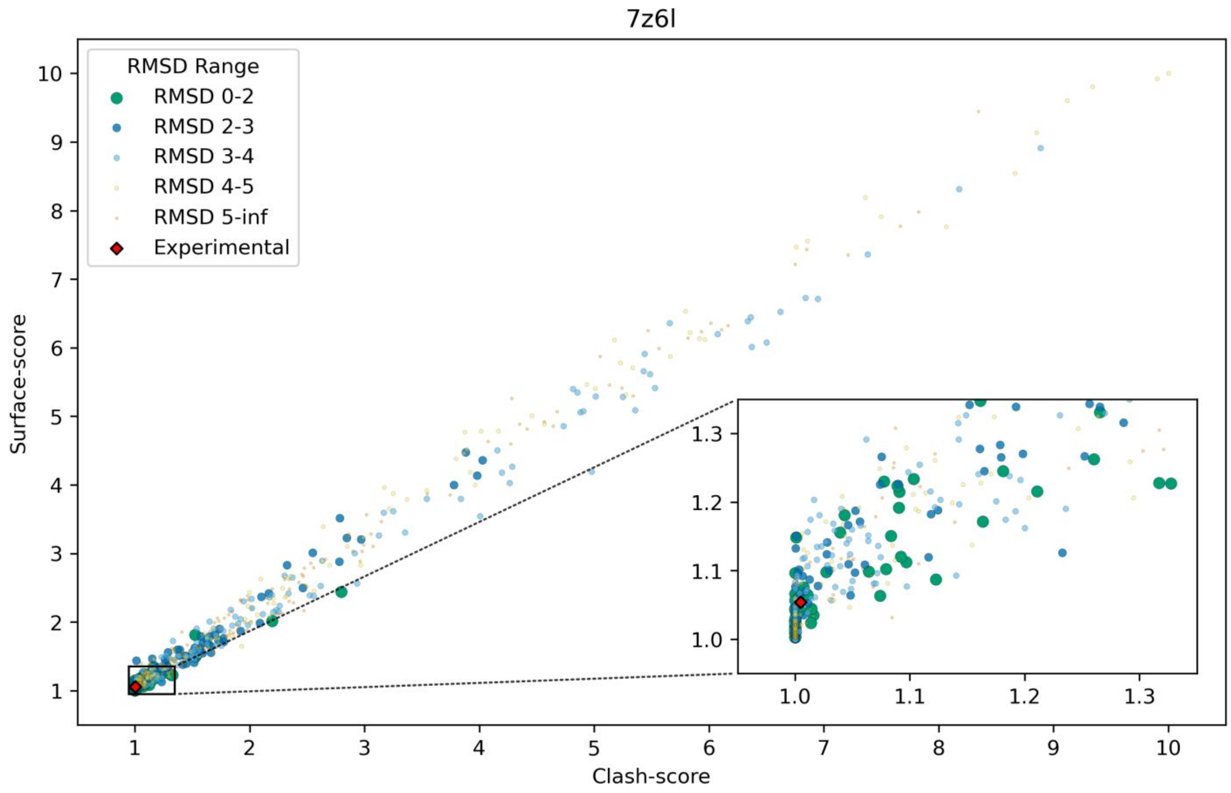

Figure S59. Surface-score vs clash-score scatterplot for the full conformational ensemble of 7Z6L. Each point represents a model generated by the PCG. Point color, size, and transparency reflect a scale of lig_RMSD (in Å). The red diamond marks the scores of the experimental ternary complex structure for reference.

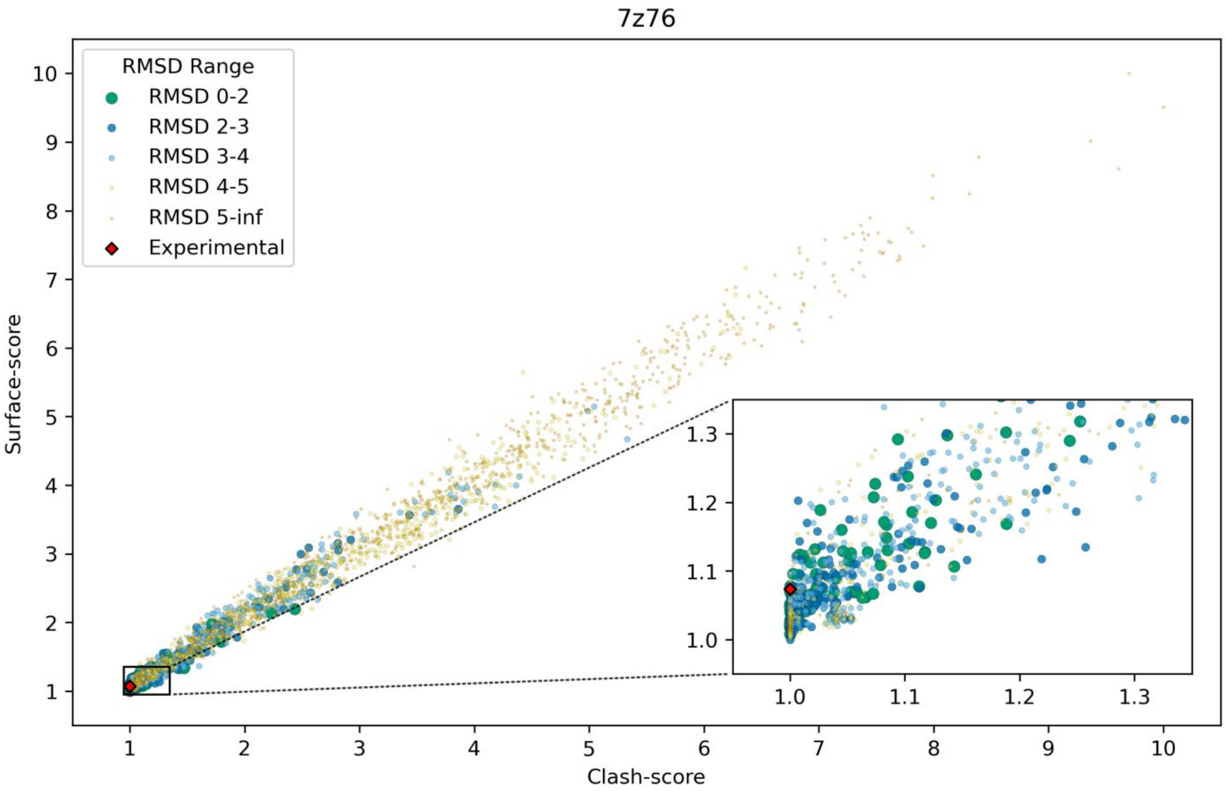

Figure S60. Surface-score vs clash-score scatterplot for the full conformational ensemble of 7Z76. Each point represents a model generated by the PCG. Point color, size, and transparency reflect a scale of lig_RMSD (in Å). The red diamond marks the scores of the experimental ternary complex structure for reference.

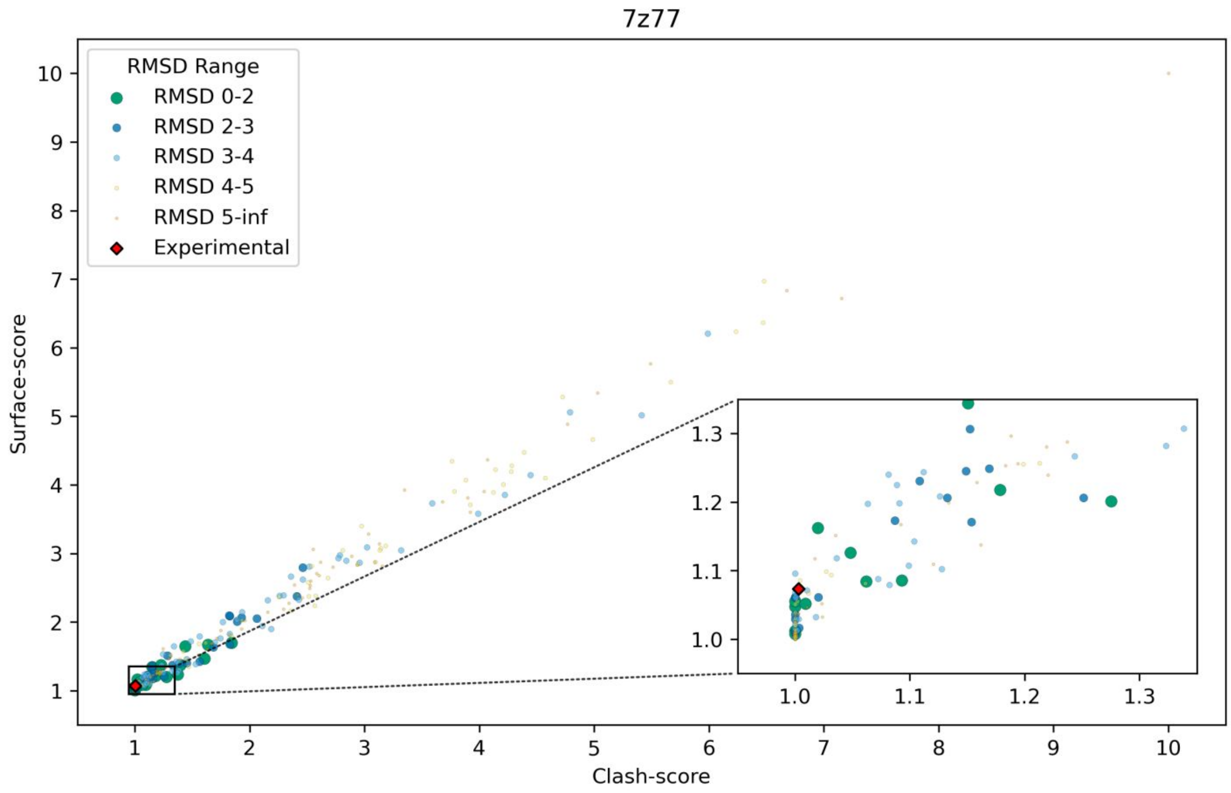

Figure S61. Surface-score vs clash-score scatterplot for the full conformational ensemble of 7Z77. Each point represents a model generated by the PCG. Point color, size, and transparency reflect a scale of lig_RMSD (in Å). The red diamond marks the scores of the experimental ternary complex structure for reference.

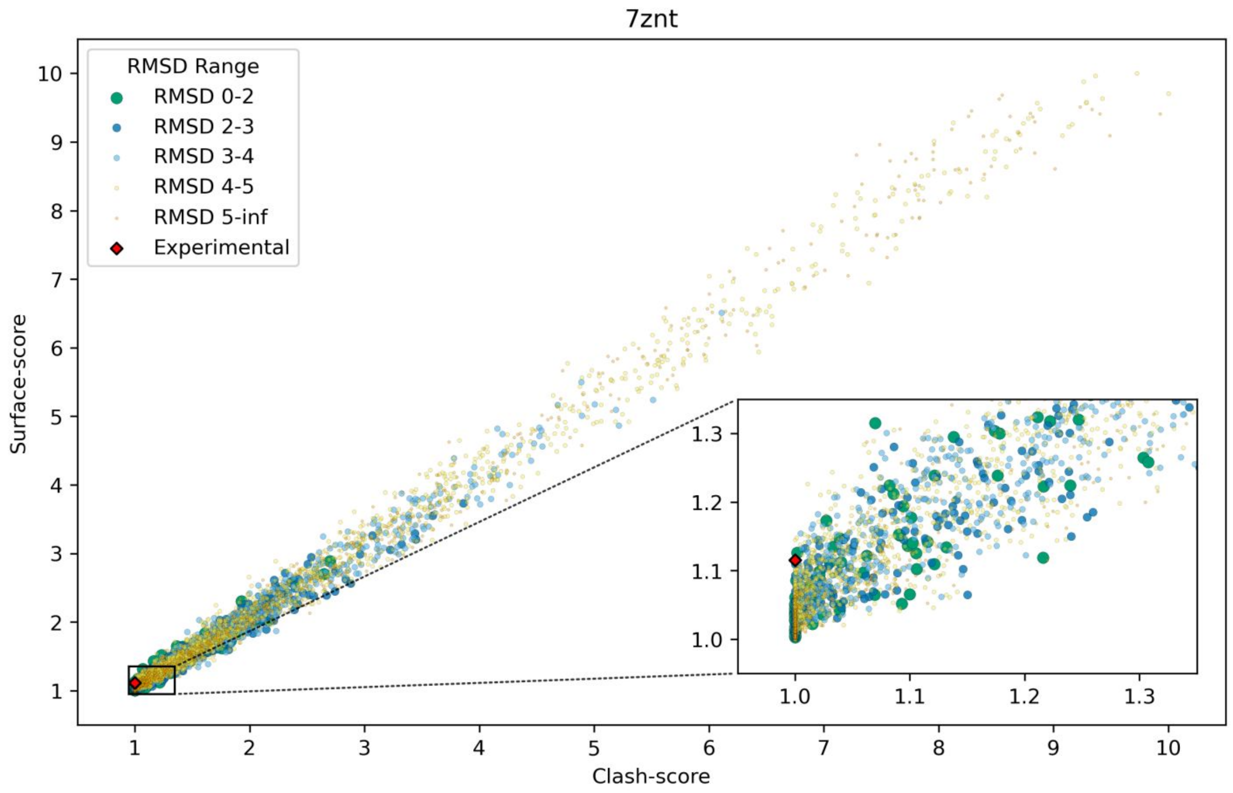

Figure S62. Surface-score vs clash-score scatterplot for the full conformational ensemble of 7ZNT. Each point represents a model generated by the PCG. Point color, size, and transparency reflect a scale of lig_RMSD (in Å). The red diamond marks the scores of the experimental ternary complex structure for reference.

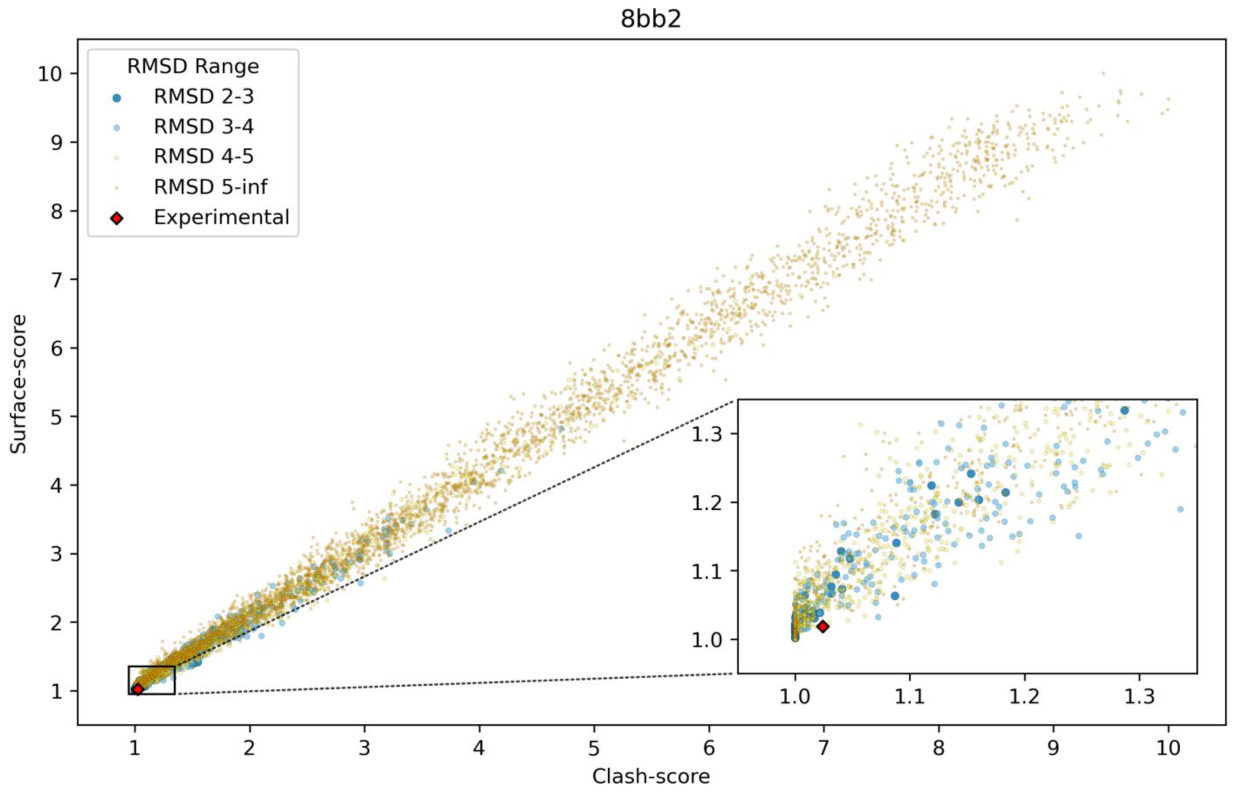

Figure S63. Surface-score vs clash-score scatterplot for the full conformational ensemble of 8BB2. Each point represents a model generated by the PCG. Point color, size, and transparency reflect a scale of lig_RMSD (in Å). The red diamond marks the scores of the experimental ternary complex structure for reference.

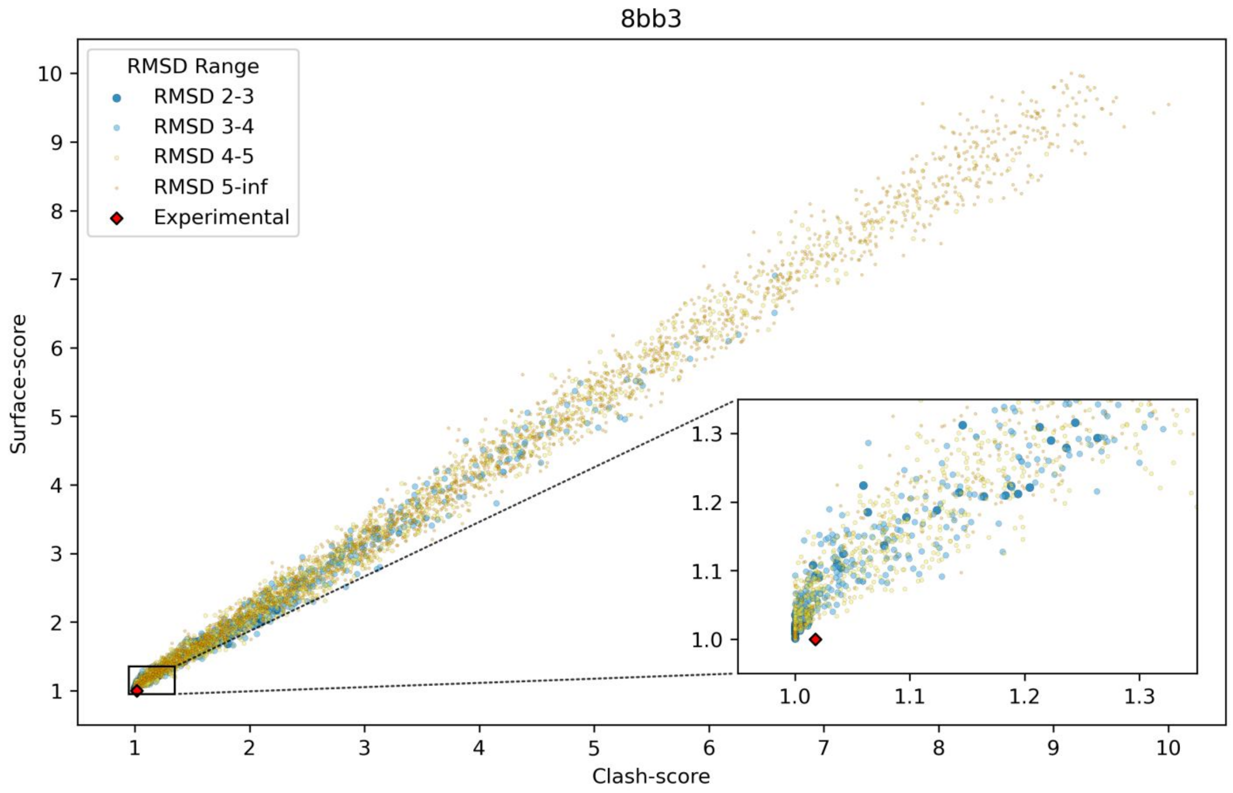

Figure S64. Surface-score vs clash-score scatterplot for the full conformational ensemble of 8BB3. Each point represents a model generated by the PCG. Point color, size, and transparency reflect a scale of lig_RMSD (in Å). The red diamond marks the scores of the experimental ternary complex structure for reference.

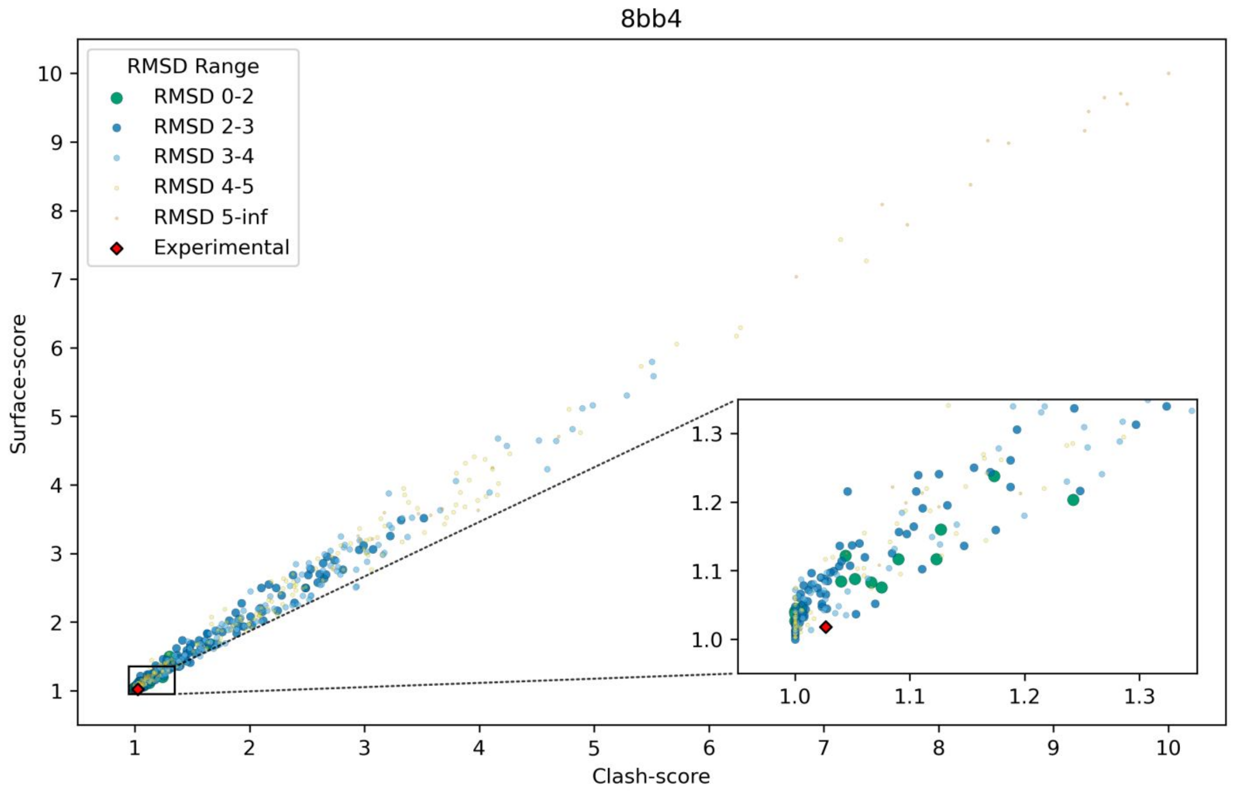

Figure S65. Surface-score vs clash-score scatterplot for the full conformational ensemble of 8BB4. Each point represents a model generated by the PCG. Point color, size, and transparency reflect a scale of lig_RMSD (in Å). The red diamond marks the scores of the experimental ternary complex structure for reference.

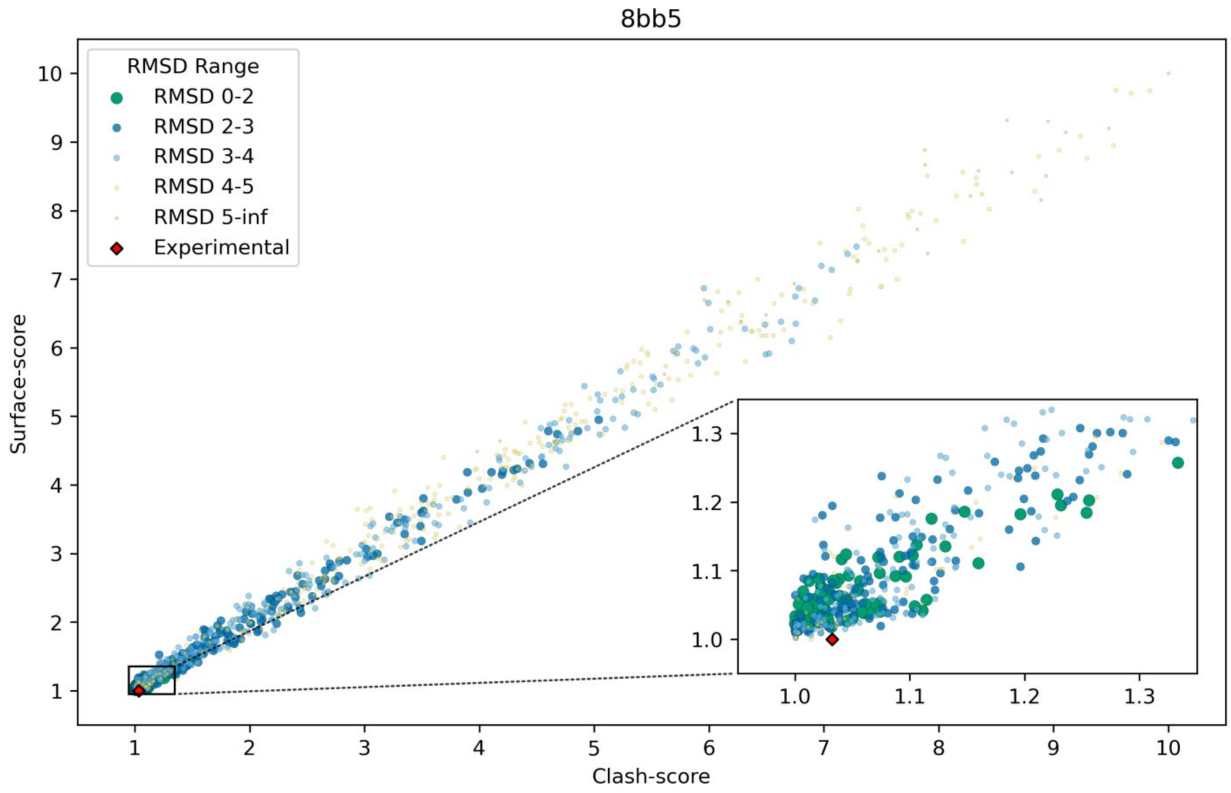

Figure S66. Surface-score vs clash-score scatterplot for the full conformational ensemble of 8BB5. Each point represents a model generated by the PCG. Point color, size, and transparency reflect a scale of lig_RMSD (in Å). The red diamond marks the scores of the experimental ternary complex structure for reference.

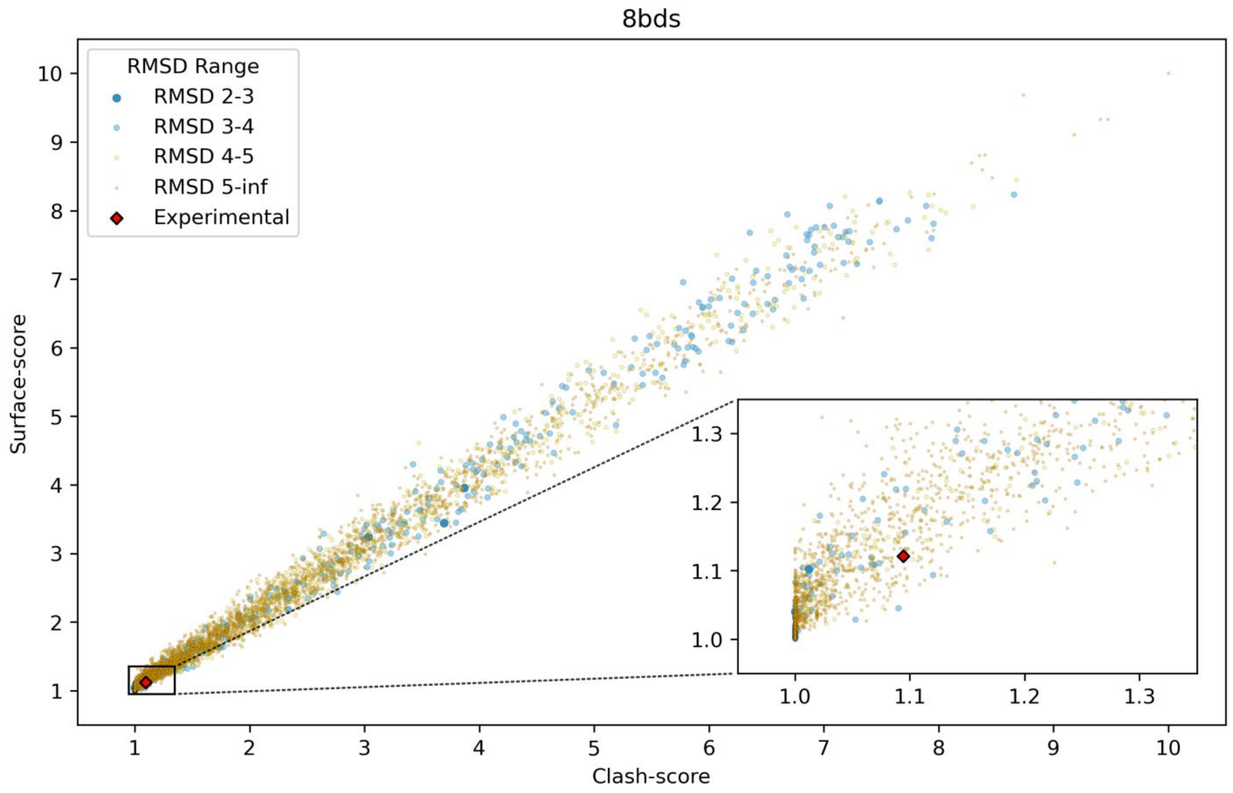

Figure S67. Surface-score vs clash-score scatterplot for the full conformational ensemble of 8BDS. Each point represents a model generated by the PCG. Point color, size, and transparency reflect a scale of lig_RMSD (in Å). The red diamond marks the scores of the experimental ternary complex structure for reference.

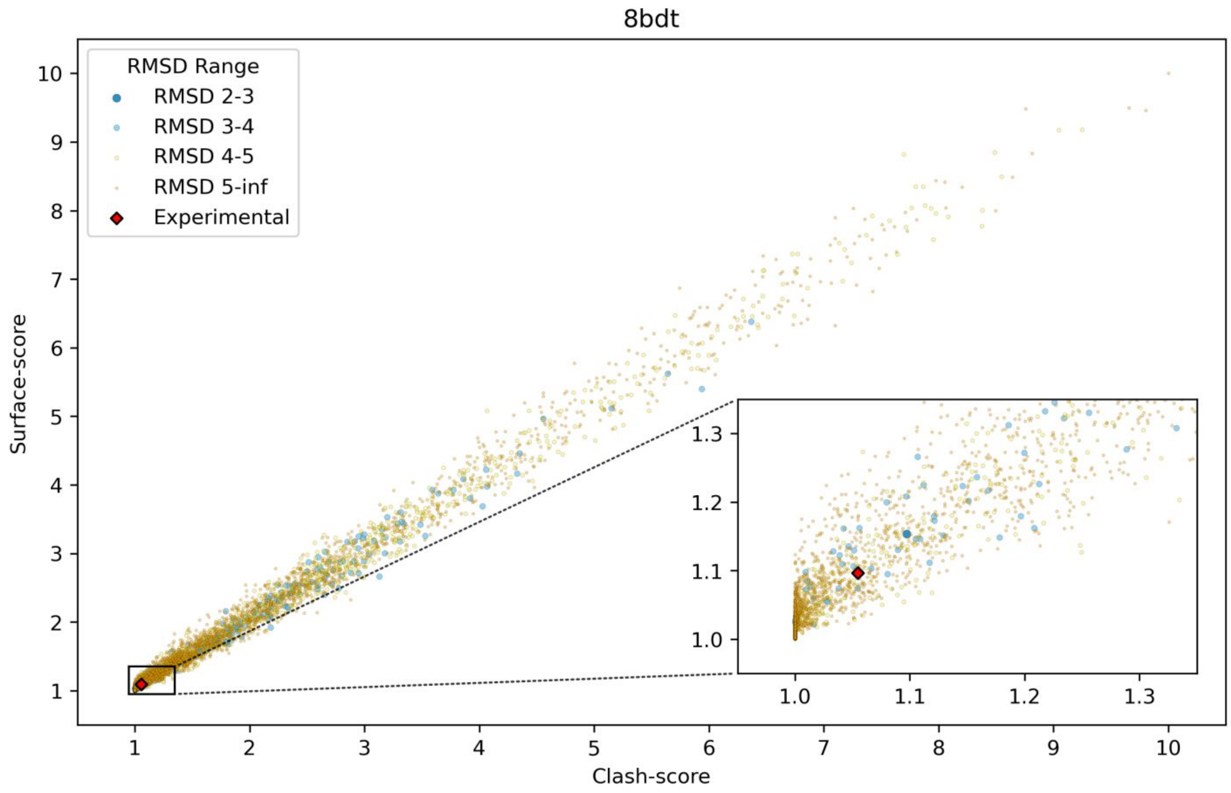

Figure S68. Surface-score vs clash-score scatterplot for the full conformational ensemble of 8BDT. Each point represents a model generated by the PCG. Point color, size, and transparency reflect a scale of lig_RMSD (in Å). The red diamond marks the scores of the experimental ternary complex structure for reference.

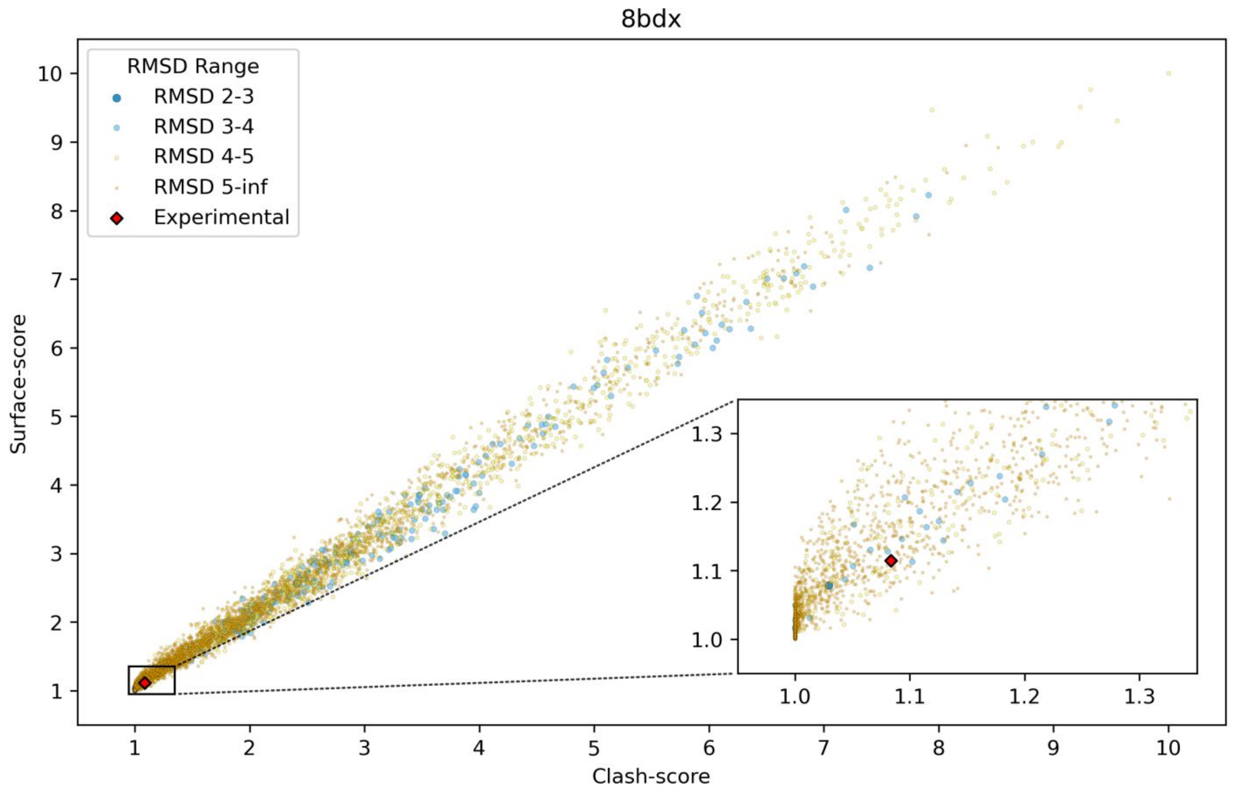

Figure S69. Surface-score vs clash-score scatterplot for the full conformational ensemble of 8BDX. Each point represents a model generated by the PCG. Point color, size, and transparency reflect a scale of lig_RMSD (in Å). The red diamond marks the scores of the experimental ternary complex structure for reference.

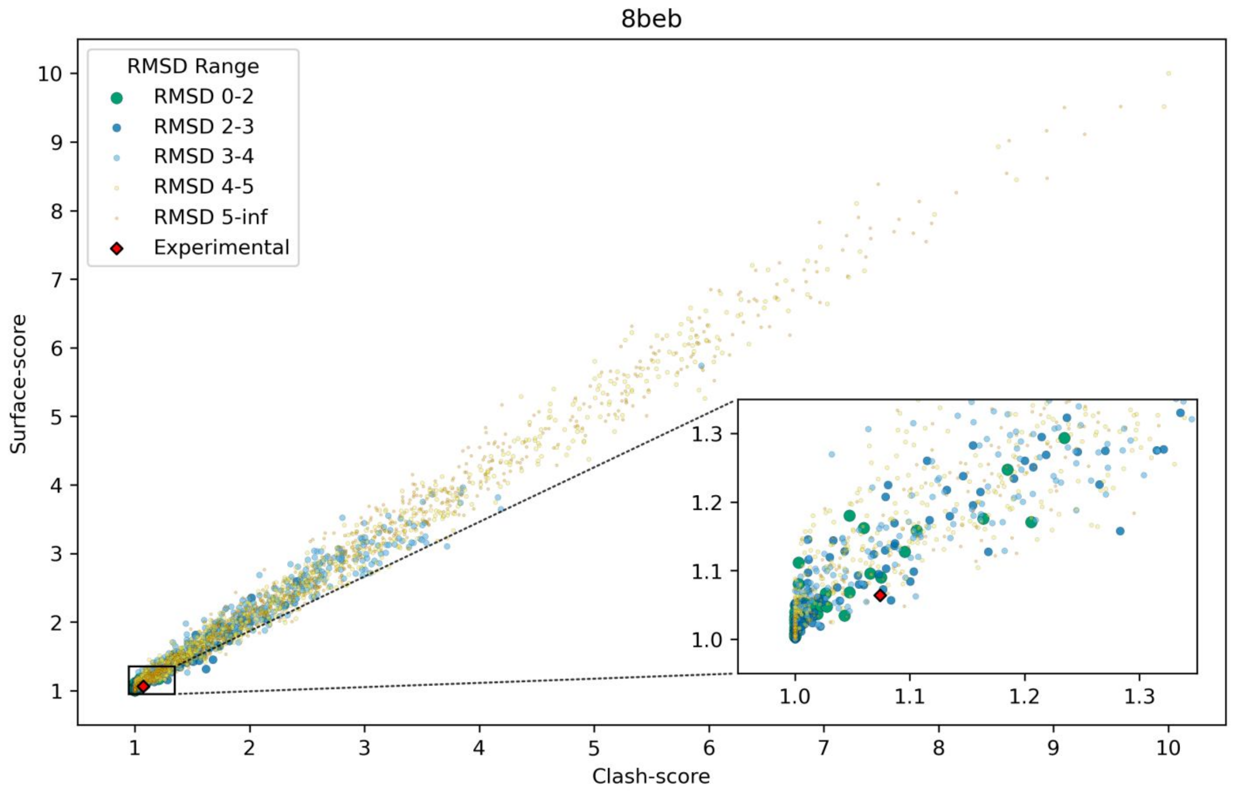

Figure S70. Surface-score vs clash-score scatterplot for the full conformational ensemble of 8BEB. Each point represents a model generated by the PCG. Point color, size, and transparency reflect a scale of lig_RMSD (in Å). The red diamond marks the scores of the experimental ternary complex structure for reference.

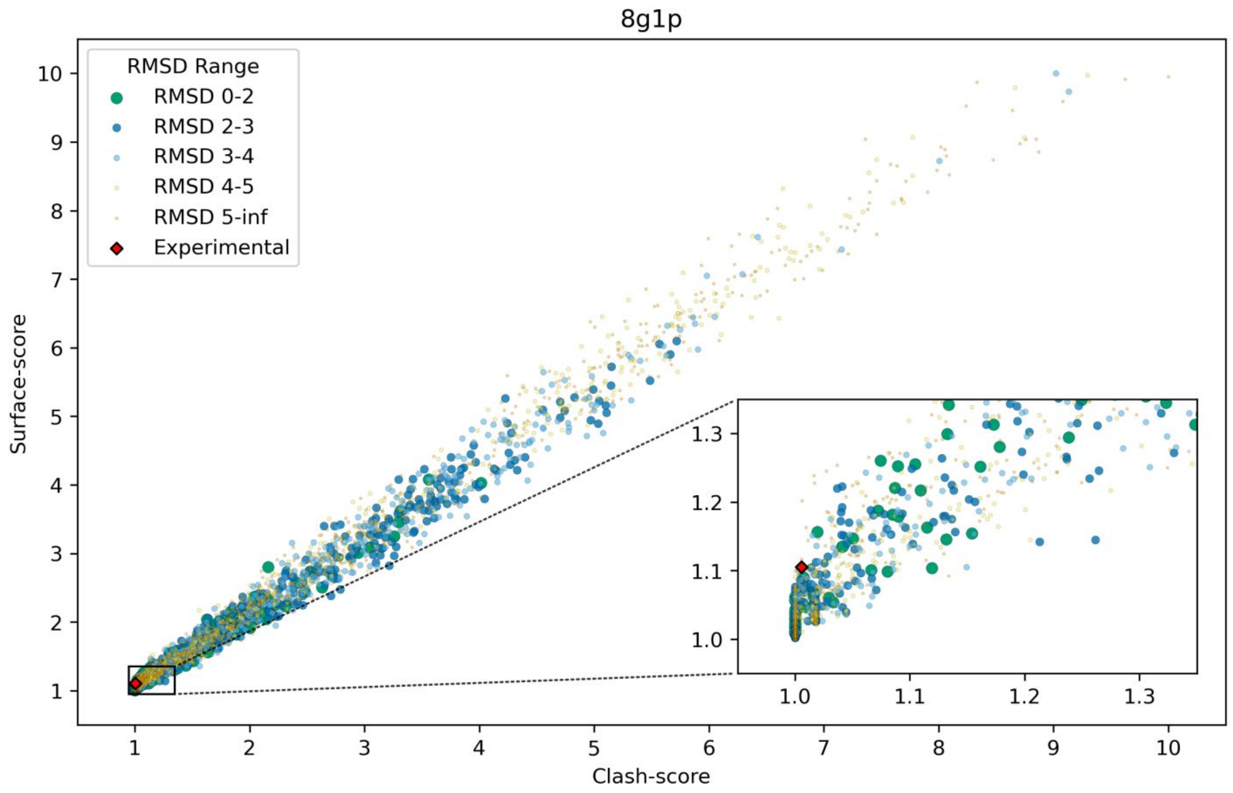

Figure S71. Surface-score vs clash-score scatterplot for the full conformational ensemble of 8G1P. Each point represents a model generated by the PCG. Point color, size, and transparency reflect a scale of lig_RMSD (in Å). The red diamond marks the scores of the experimental ternary complex structure for reference.

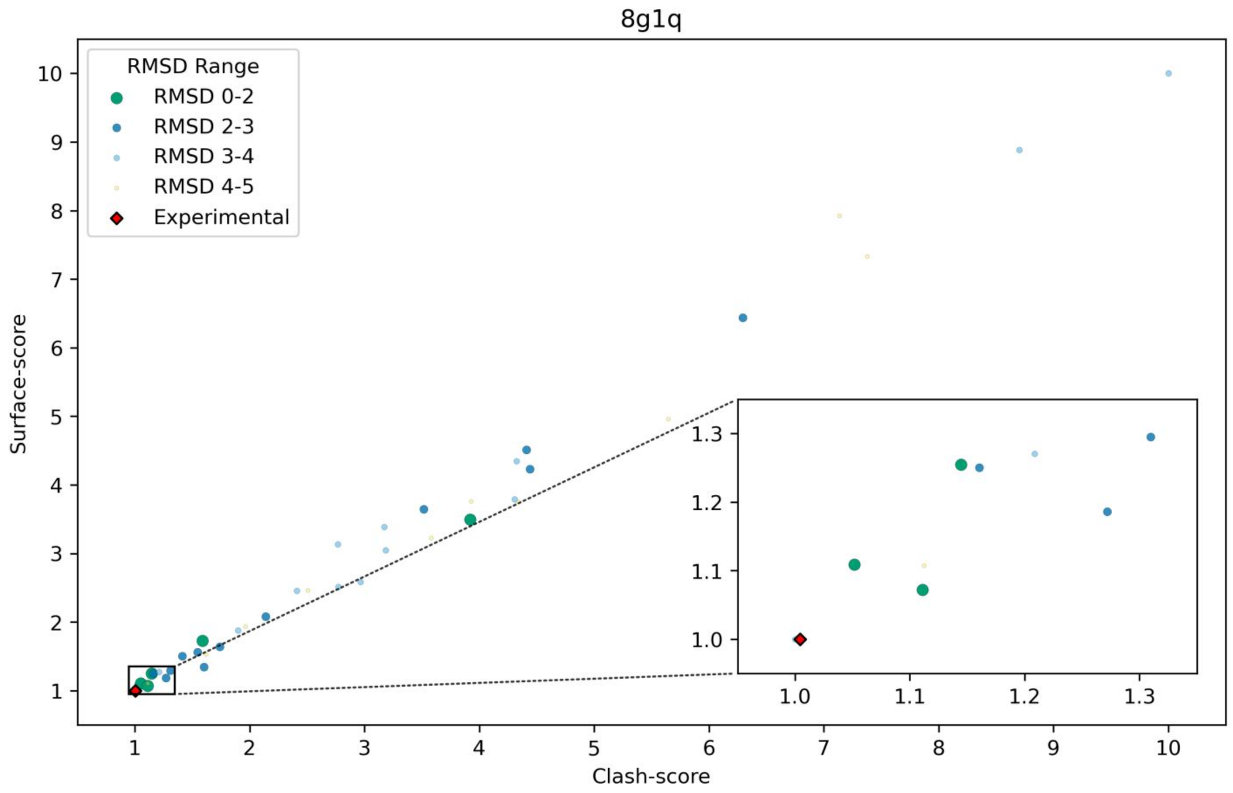

Figure S72. Surface-score vs clash-score scatterplot for the full conformational ensemble of 8G1Q. Each point represents a model generated by the PCG. Point color, size, and transparency reflect a scale of lig_RMSD (in Å). The red diamond marks the scores of the experimental ternary complex structure for reference.

Overlay of generated ternary complex models to the experimental structures

This section presents structural comparisons between the experimental PROTAC-mediated ternary complexes and the models generated by the PCG. The structures were aligned on the E3 ligase side using PyMOL's align command, which performs a least-squares fit based on Cα atoms (ligand excluded), to highlight variations in the positioning and orientation of the POI. Experimental structures are shown in green, and the model with the lowest pp_RMSD is shown in magenta. The model based on the closest ligand conformation is also shown in cyan for comparison. In cases where the closest conformer also yields the lowest pp_RMSD (6HAX and 7Z76), only the cyan model is shown alongside the experimental structure. In figures that include a second panel, steric clashes are visualized for the model built on the closest conformer, except for the 6BN7 case, where clashes are shown for the lowest clash-score model to illustrate that they are present across the entire ensemble. The E3 ligase is shown in yellow, the POI in orange, and the PROTAC in light gray carbon sticks with standard heteroatom coloring. Regions of steric conflict are circled in red.

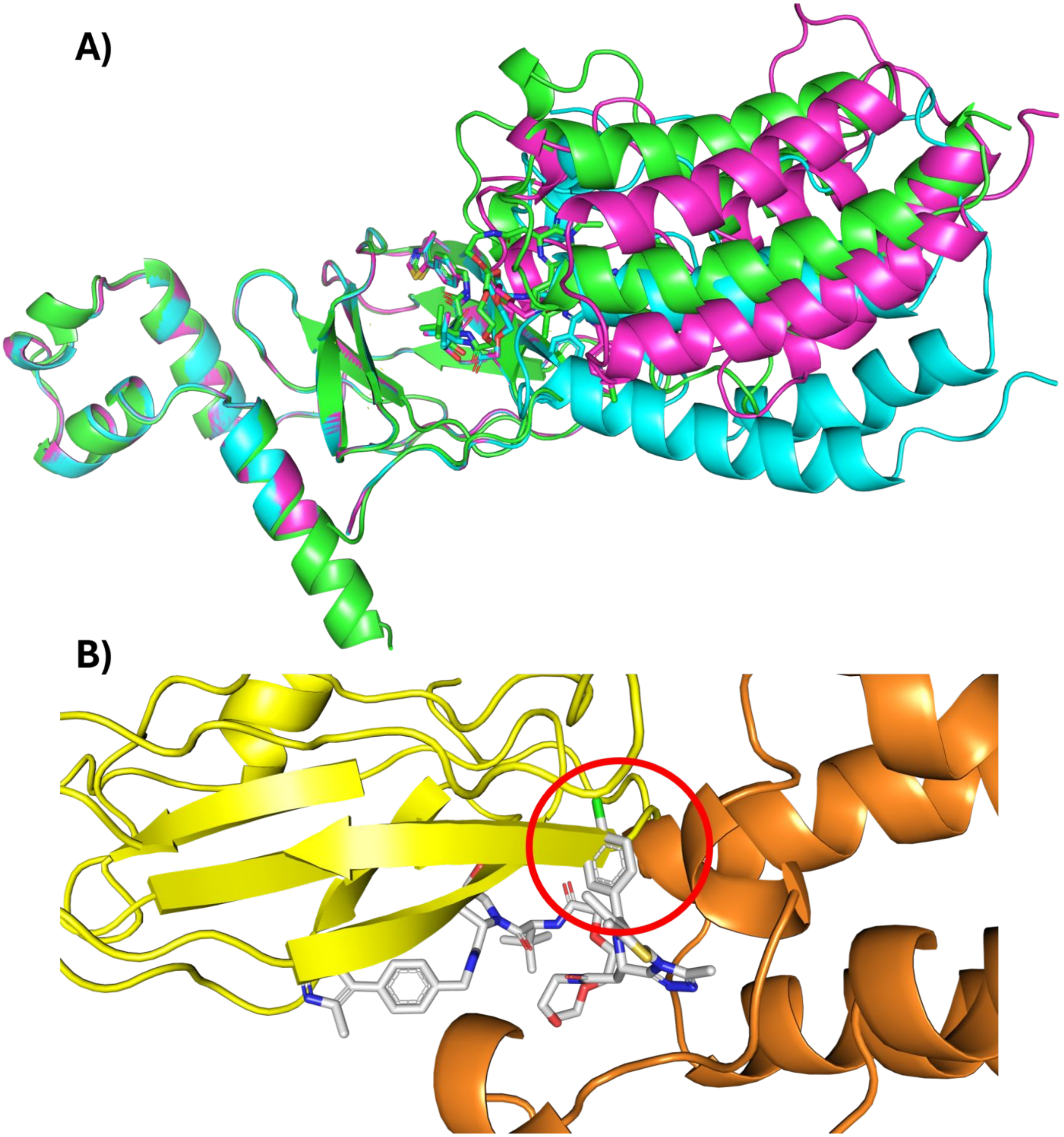

Figure S73. A) Overlay of the experimental ternary complex of 5T35 (green), the model based on the closest conformer (cyan), and the model with the lowest pp_RMSD (magenta), aligned on the E3 ligase side. **B)** Closest conformer-based model shown separately, with steric clashes circled in

red. The E3 ligase is shown in yellow, the POI in orange, and the PROTAC in light gray carbon sticks.

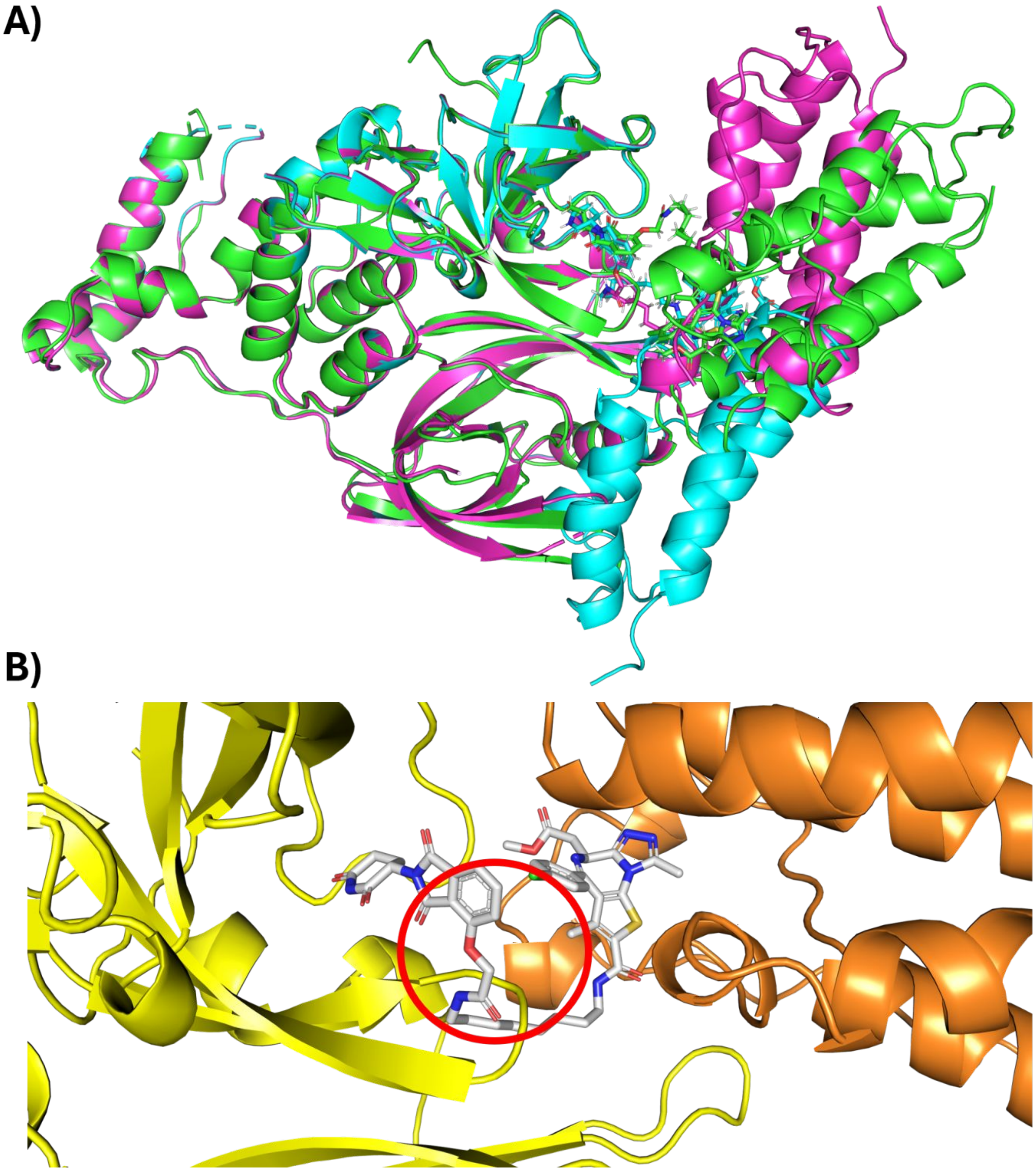

Figure S74. A) Overlay of the experimental ternary complex of 6BN7 (green), the model based on the closest conformer (cyan), and the model with the lowest pp_RMSD (magenta), aligned on the E3 ligase side. **B)** Lowest clash-score model with steric clashes circled in red. The E3 ligase is shown in yellow, the POI in orange, and the PROTAC in light gray carbon sticks.

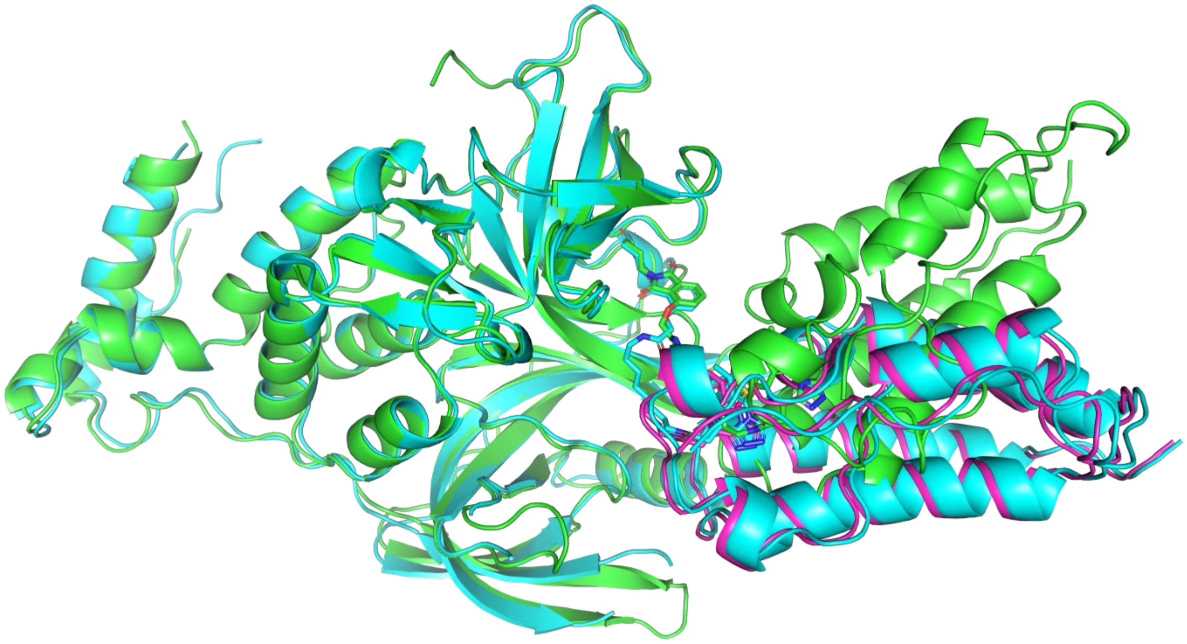

Figure S75. Overlay of the experimental ternary complex of 6BOY (green), the model based on the closest conformer (cyan), and the model with the lowest pp_RMSD (magenta), aligned on the E3 ligase side.

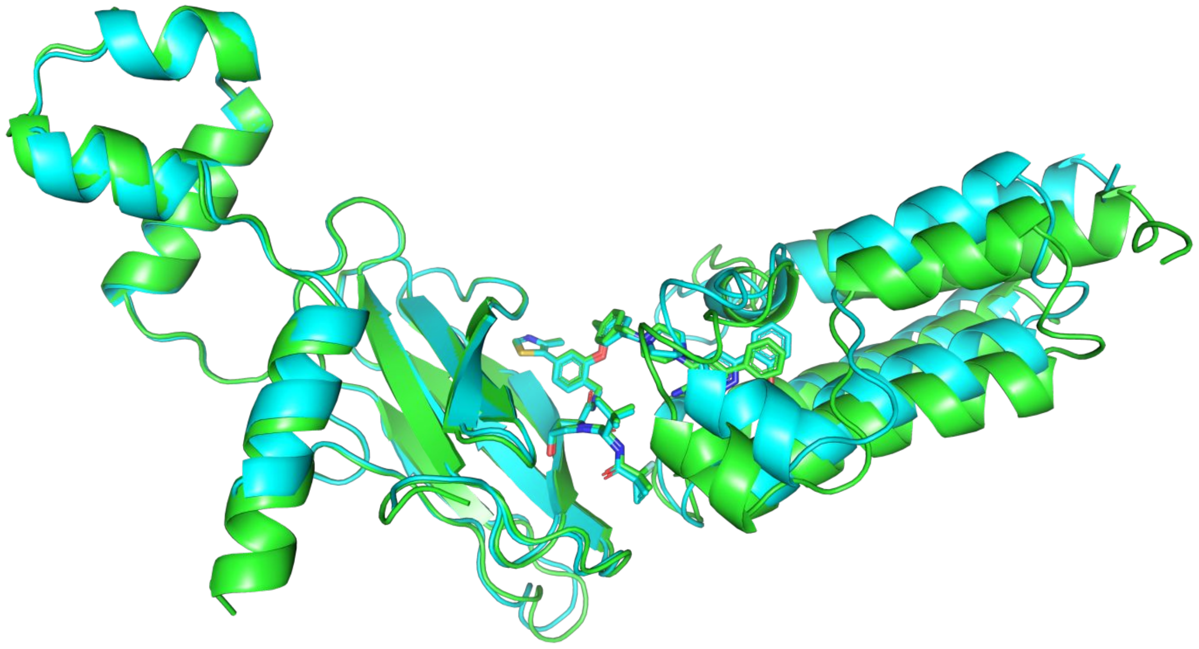

Figure S76. Overlay of the experimental ternary complex of 6HAX (green) and the model based on the closest conformer, which also has the lowest pp_RMSD (cyan), aligned on the E3 ligase side.

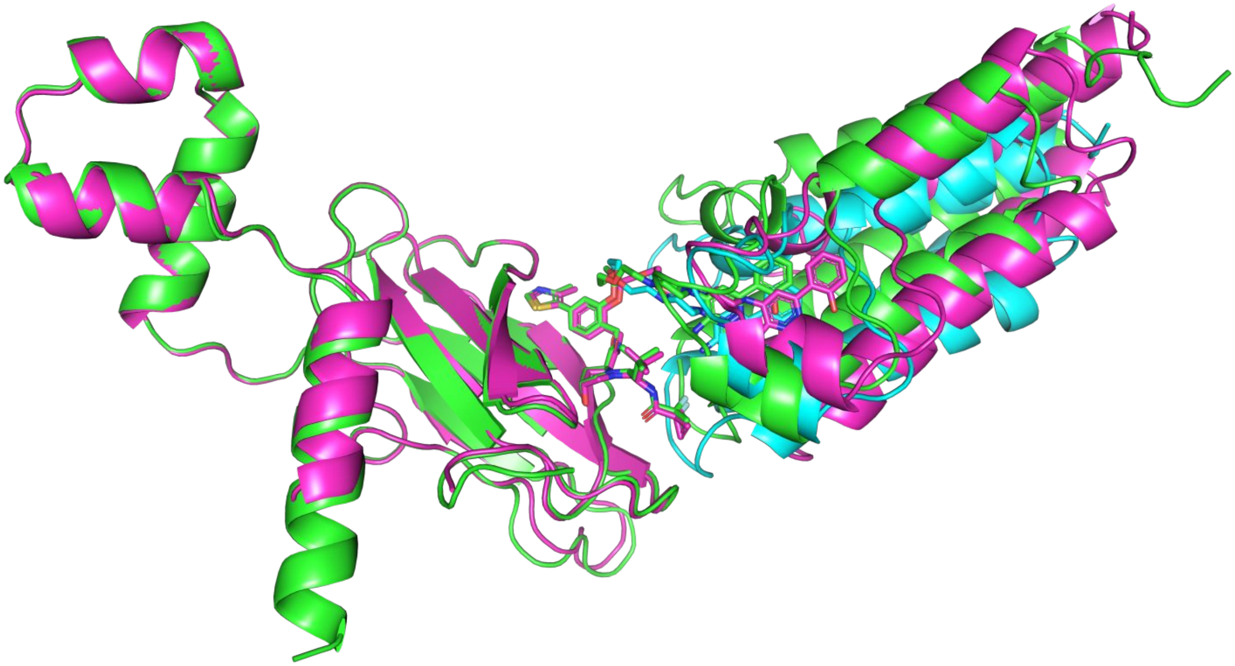

Figure S77. Overlay of the experimental ternary complex of 6HAY (green), the model based on the closest conformer (cyan), and the model with the lowest pp_RMSD (magenta), aligned on the E3 ligase side.

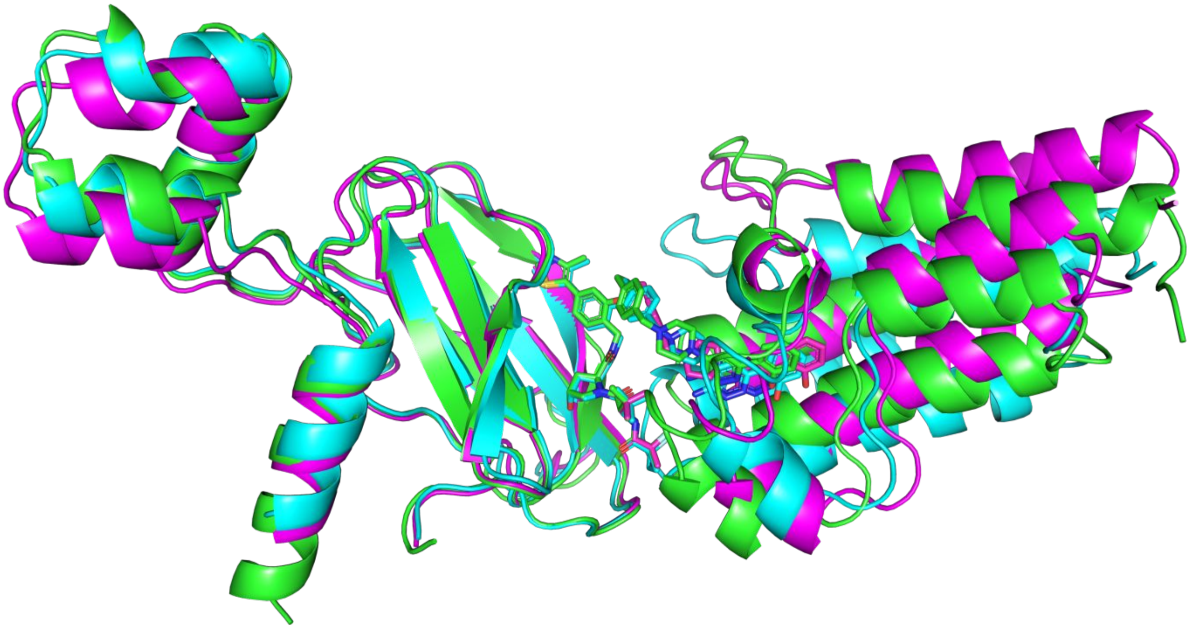

Figure S78. Overlay of the experimental ternary complex of 6HR2 (green), the model based on the closest conformer (cyan), and the model with the lowest pp_RMSD (magenta), aligned on the E3 ligase side.

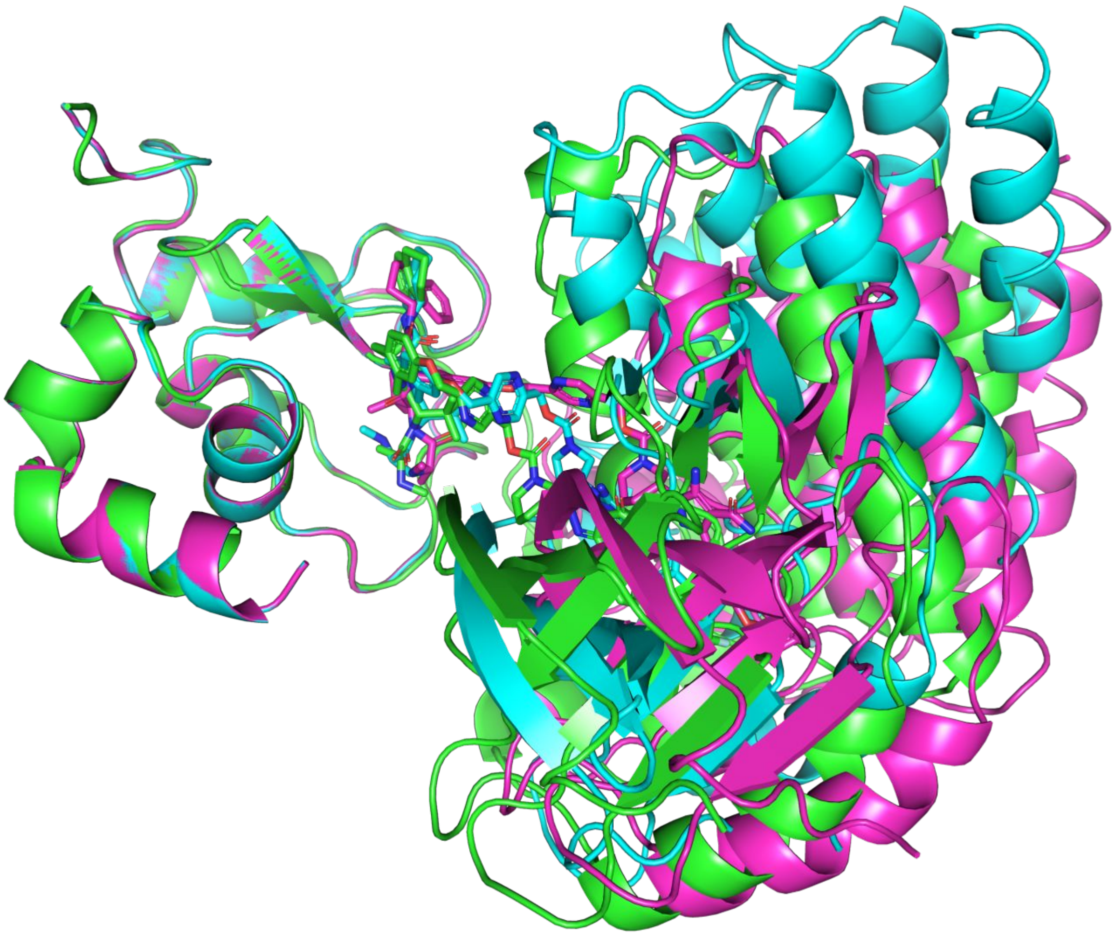

Figure S79. Overlay of the experimental ternary complex of 6W7O (green), the model based on the closest conformer (cyan), and the model with the lowest pp_RMSD (magenta), aligned on the E3 ligase side.

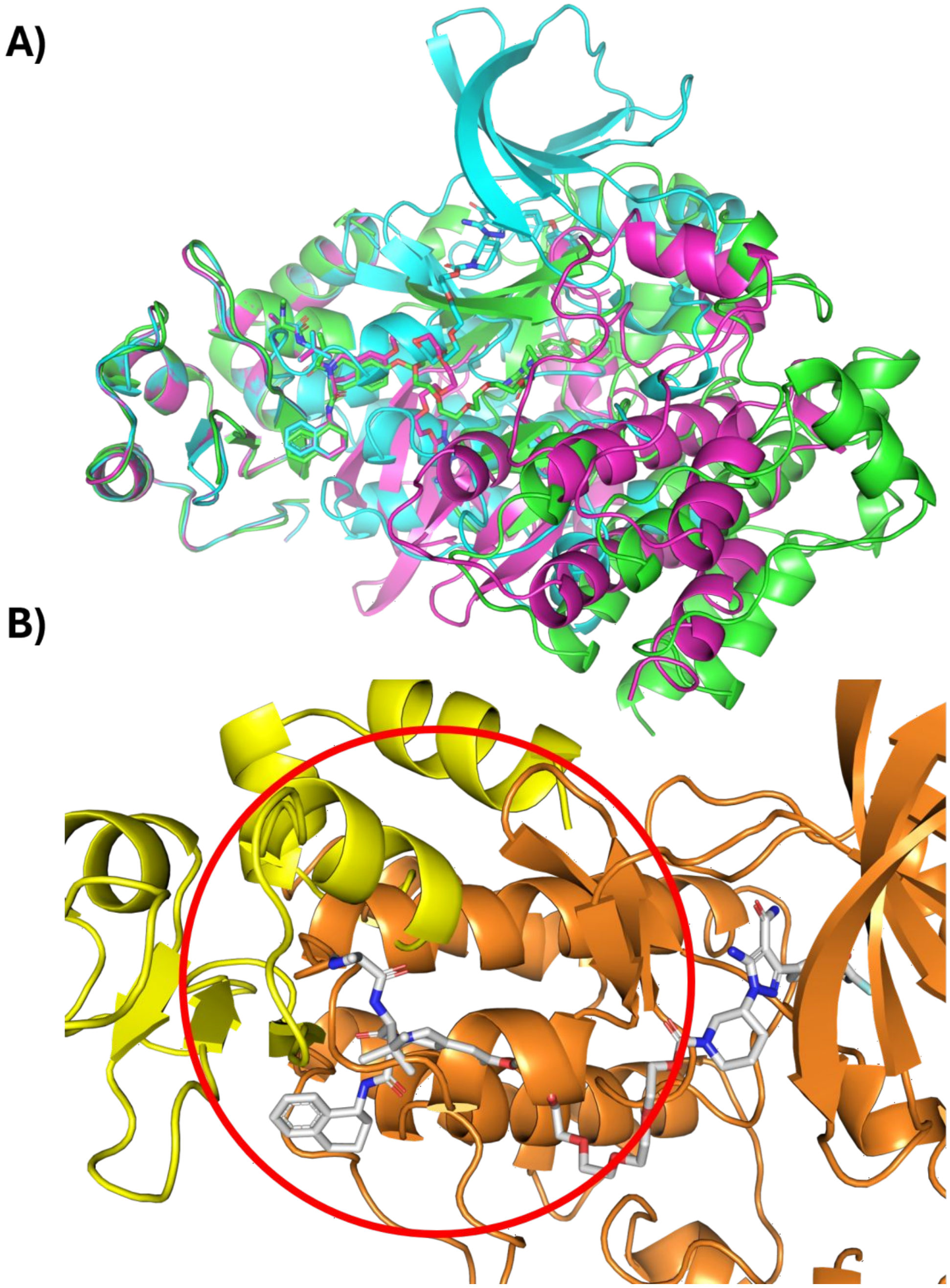

Figure S80. A) Overlay of the experimental ternary complex of 6W8I (green), the model based on the closest conformer (cyan), and the model with the lowest pp_RMSD (magenta), aligned on the E3 ligase side. **B)** Closest conformer-based model shown separately, with steric clashes circled in

red. The E3 ligase is shown in yellow, the POI in orange, and the PROTAC in light gray carbon sticks.

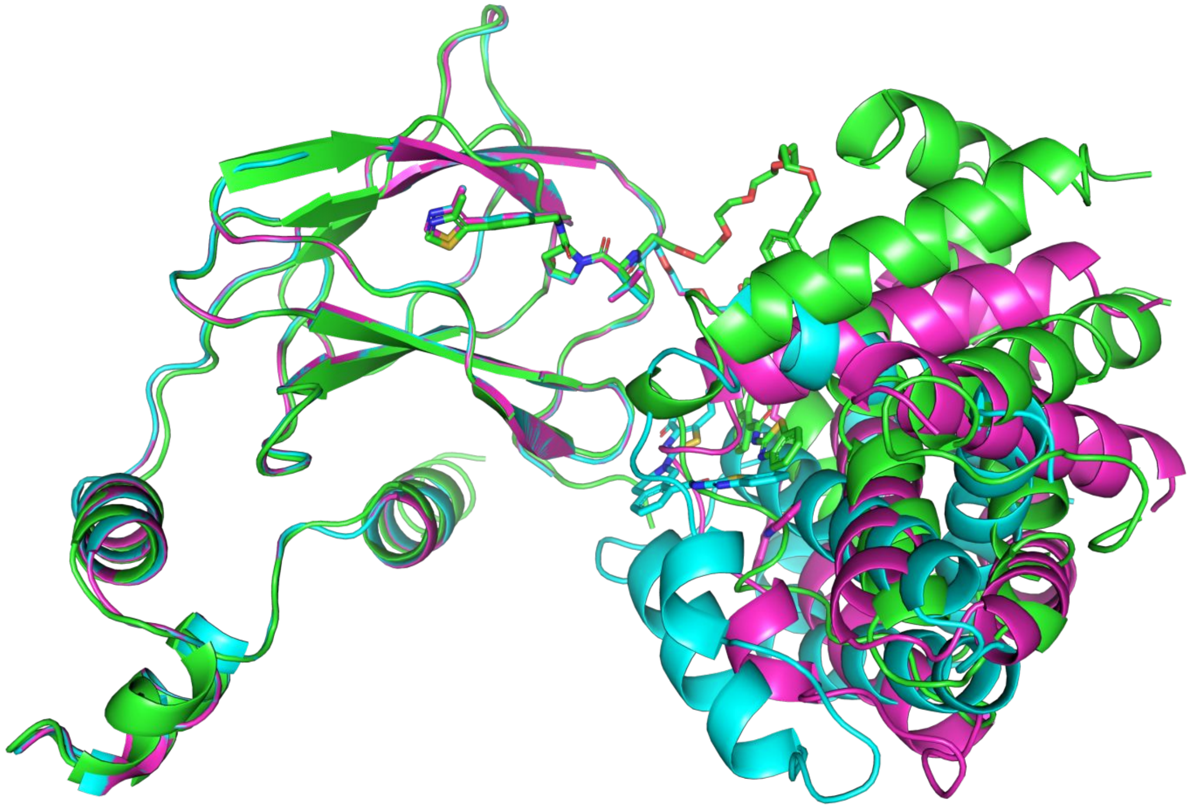

Figure S81. Overlay of the experimental ternary complex of 6ZHC (green), the model based on the closest conformer (cyan), and the model with the lowest pp_RMSD (magenta), aligned on the E3 ligase side.

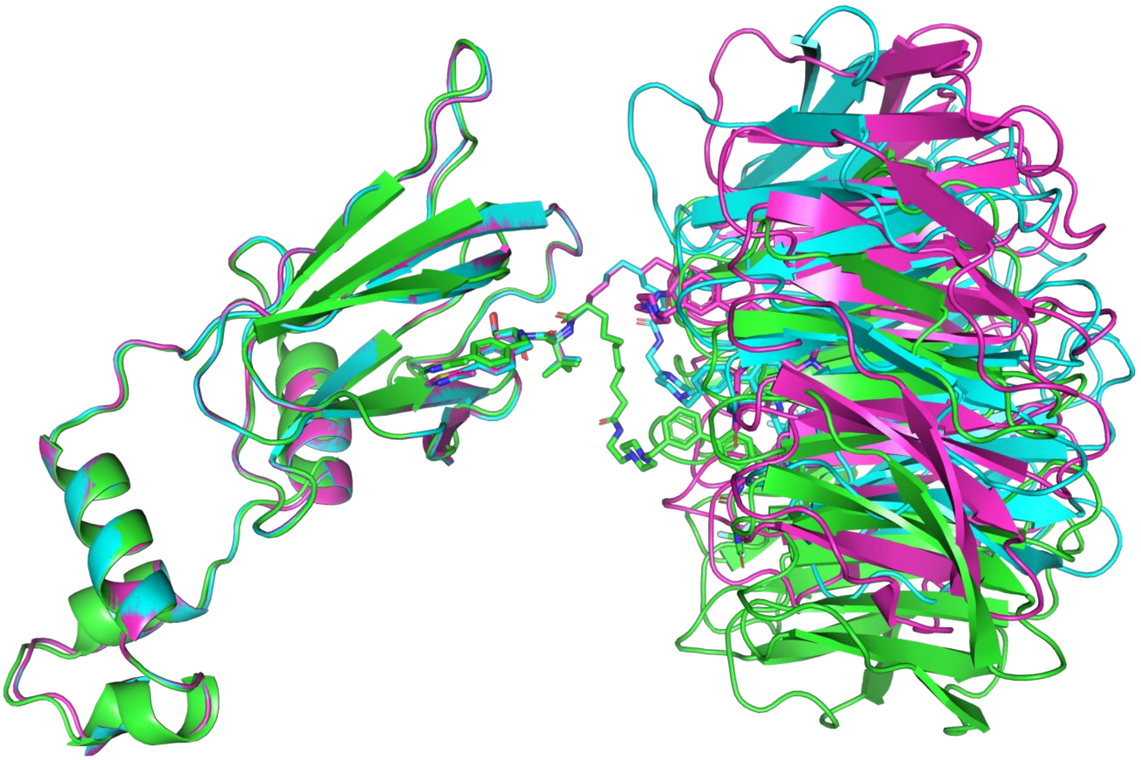

Figure S82. Overlay of the experimental ternary complex of 7JTO (green), the model based on the closest conformer (cyan), and the model with the lowest pp_RMSD (magenta), aligned on the E3 ligase side.

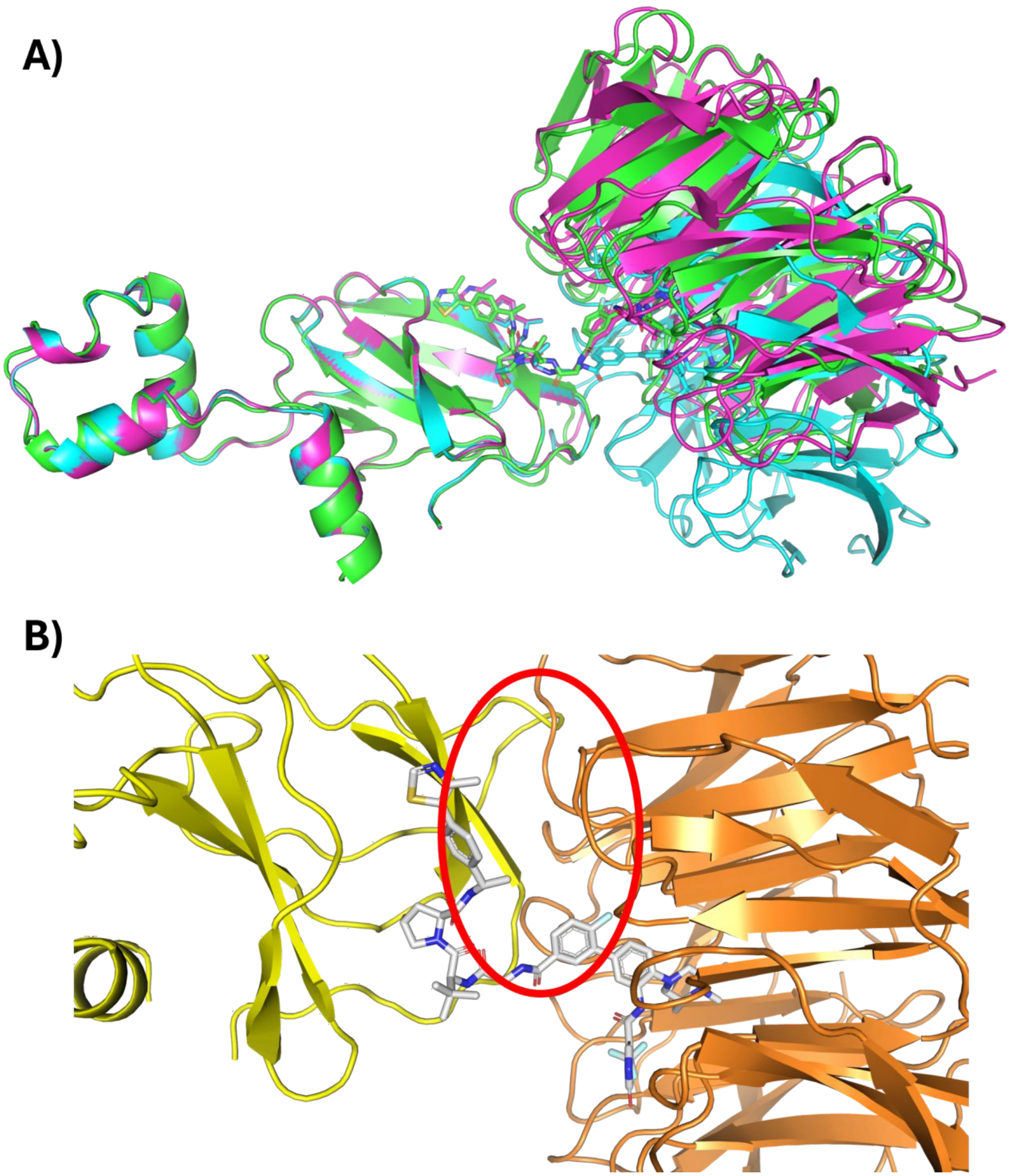

Figure S83. A) Overlay of the experimental ternary complex of 7JTP (green), the model based on the closest conformer (cyan), and the model with the lowest pp_RMSD (magenta), aligned on the E3 ligase side. **B)** Closest conformer-based model shown separately, with steric clashes circled in red. The E3 ligase is shown in yellow, the POI in orange, and the PROTAC in light gray carbon sticks.

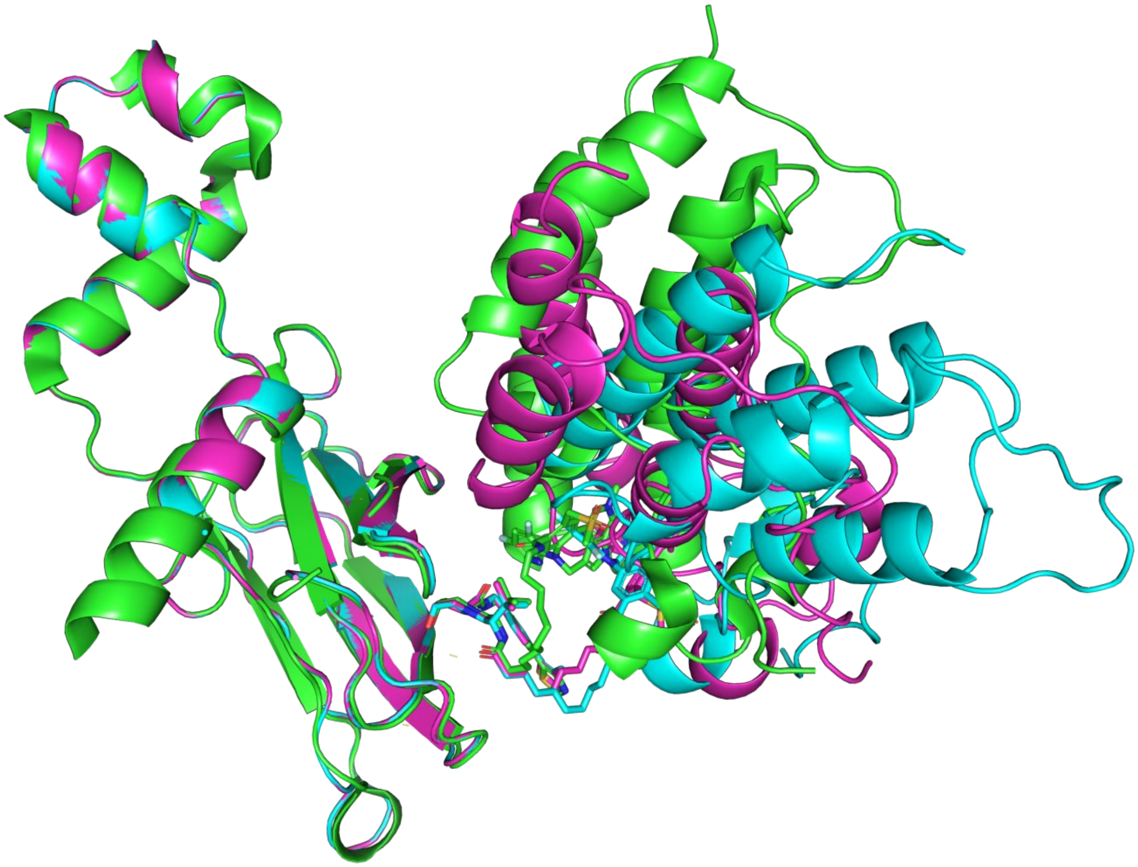

Figure S84. Overlay of the experimental ternary complex of 7KHH (green), the model based on the closest conformer (cyan), and the model with the lowest pp_RMSD (magenta), aligned on the E3 ligase side.

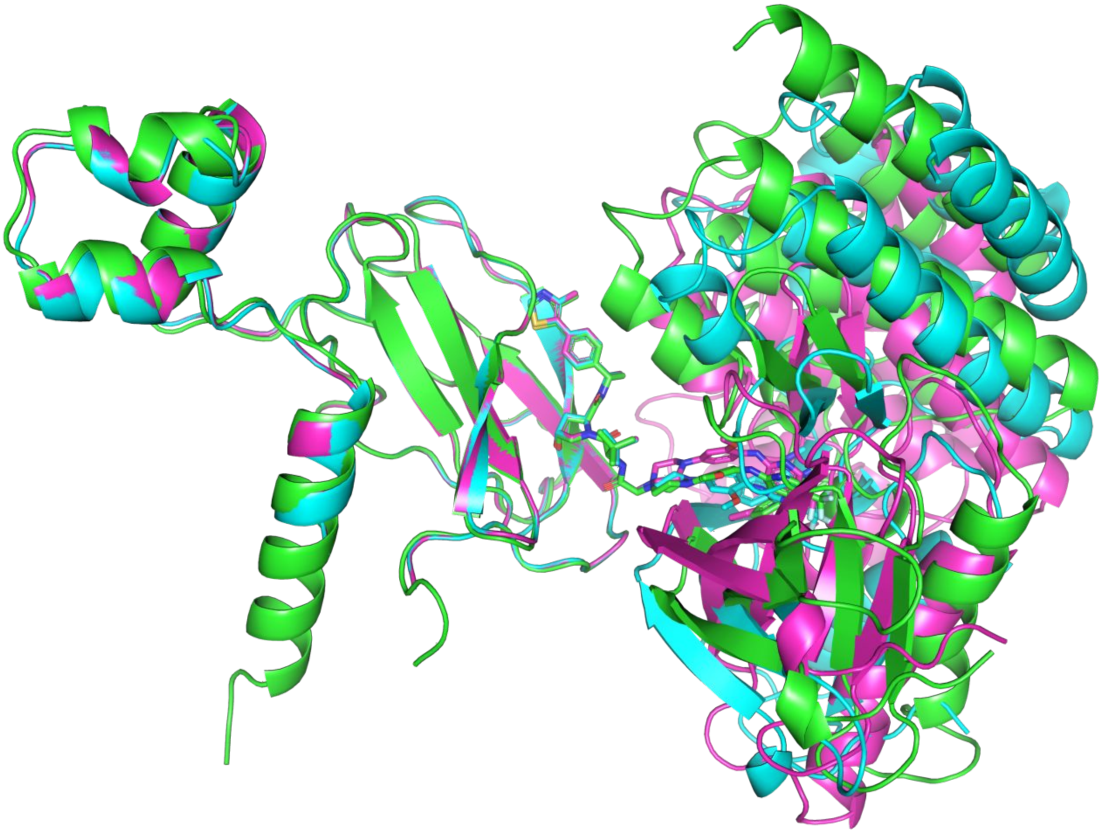

Figure S85. Overlay of the experimental ternary complex of 7PI4 (green), the model based on the closest conformer (cyan), and the model with the lowest pp_RMSD (magenta), aligned on the E3 ligase side.

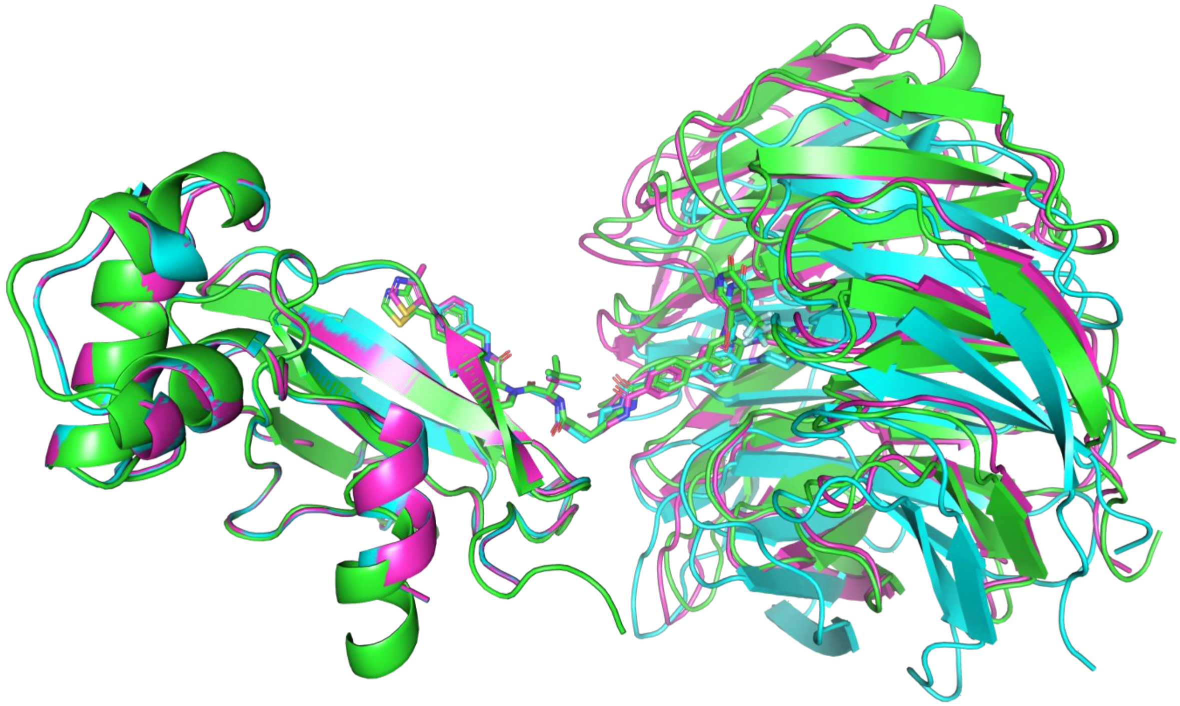

Figure S86. Overlay of the experimental ternary complex of 7Q2J (green), the model based on the closest conformer (cyan), and the model with the lowest pp_RMSD (magenta), aligned on the E3 ligase side.

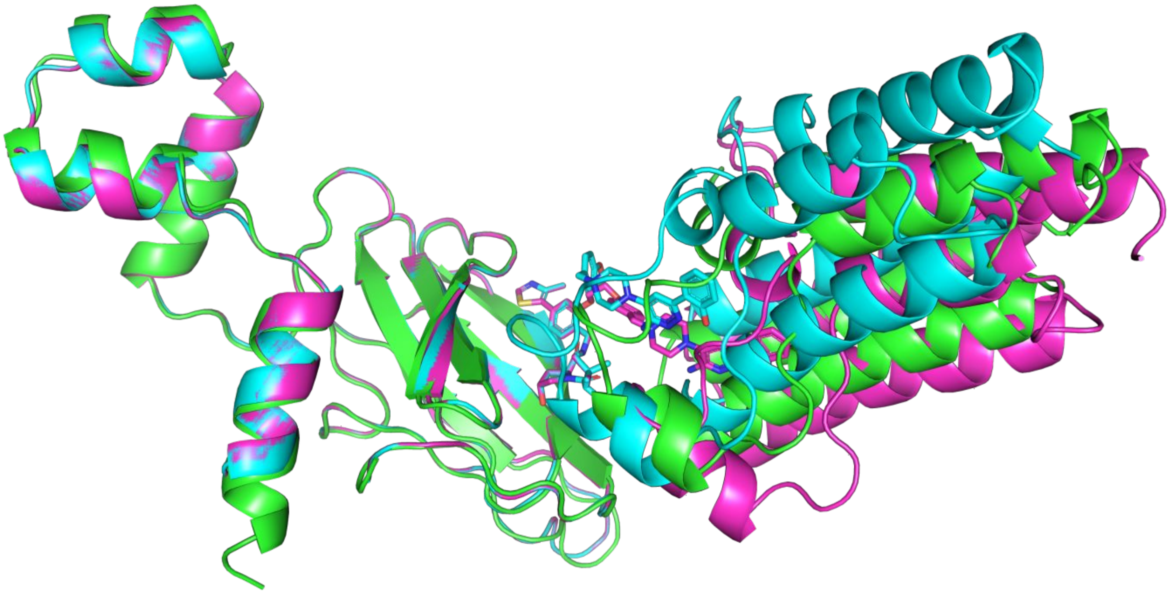

Figure S87. Overlay of the experimental ternary complex of 7S4E (green), the model based on the closest conformer (cyan), and the model with the lowest pp_RMSD (magenta), aligned on the E3 ligase side.

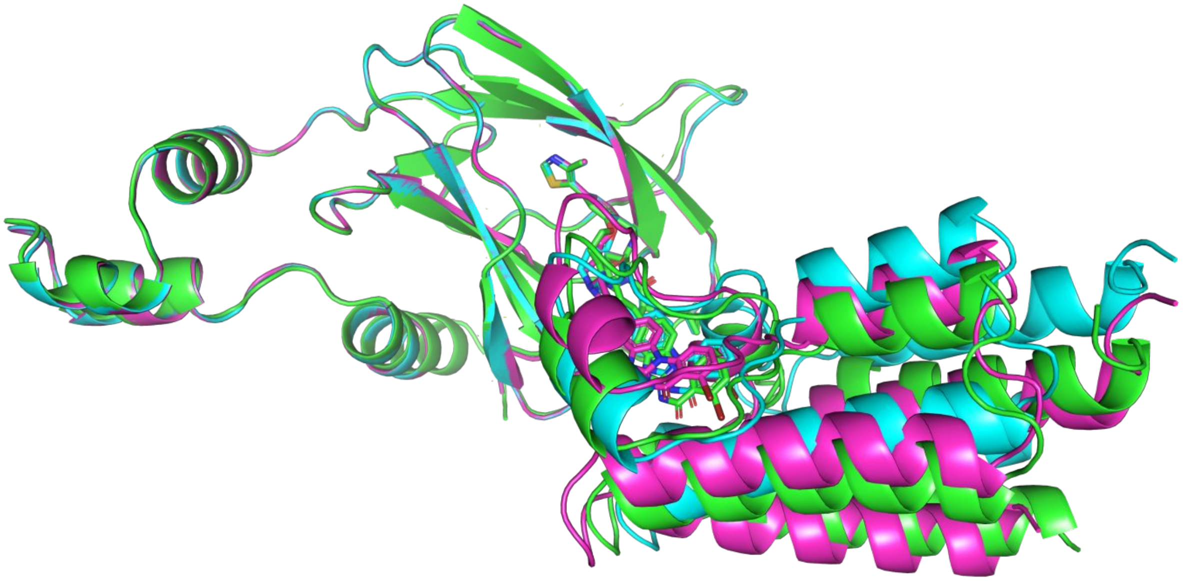

Figure S88. Overlay of the experimental ternary complex of 7Z6L (green), the model based on the closest conformer (cyan), and the model with the lowest pp_RMSD (magenta), aligned on the E3 ligase side.

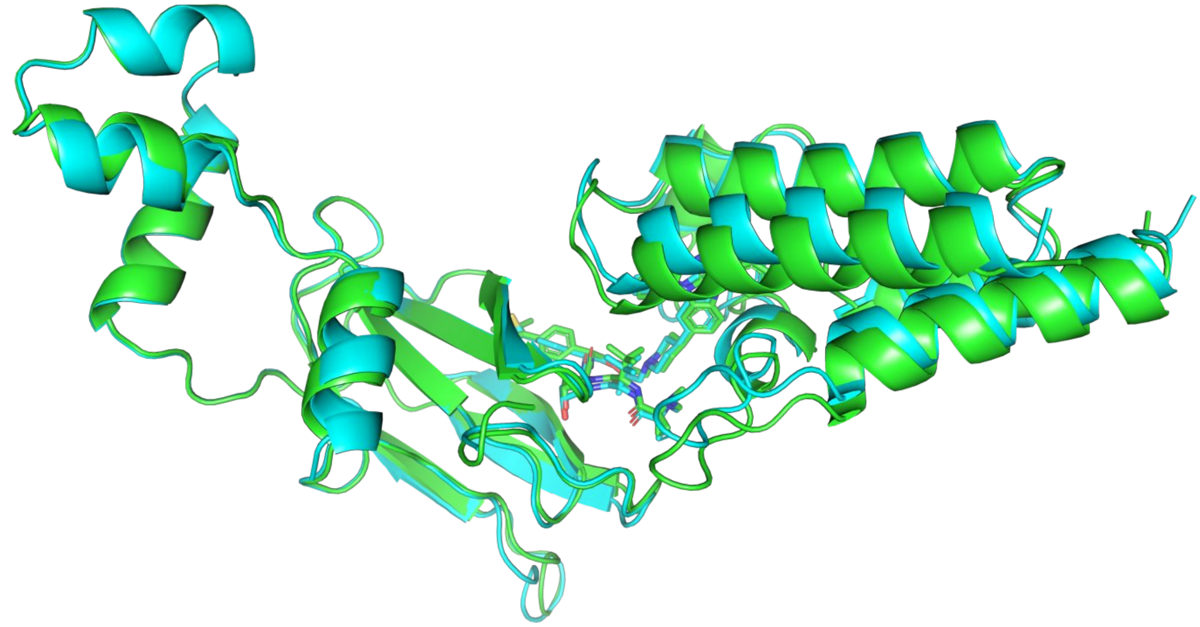

Figure S89. Overlay of the experimental ternary complex of 7Z76 (green) and the model based on the closest conformer, which also has the lowest pp_RMSD (cyan), aligned on the E3 ligase side.

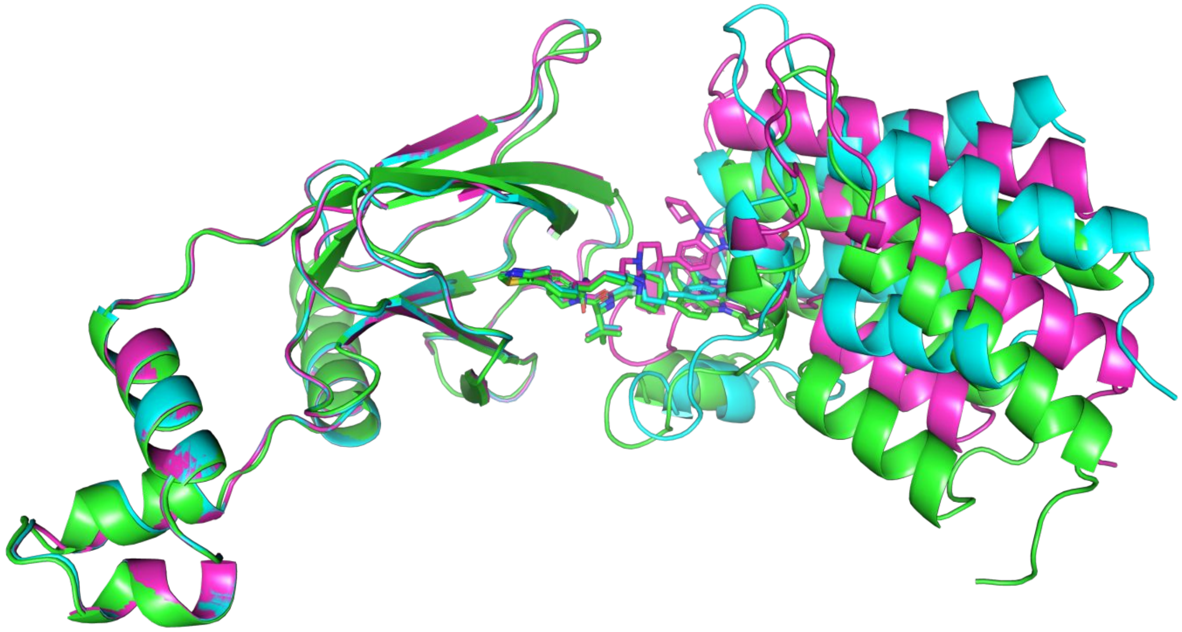

Figure S90. Overlay of the experimental ternary complex of 7Z77 (green), the model based on the closest conformer (cyan), and the model with the lowest pp_RMSD (magenta), aligned on the E3 ligase side.

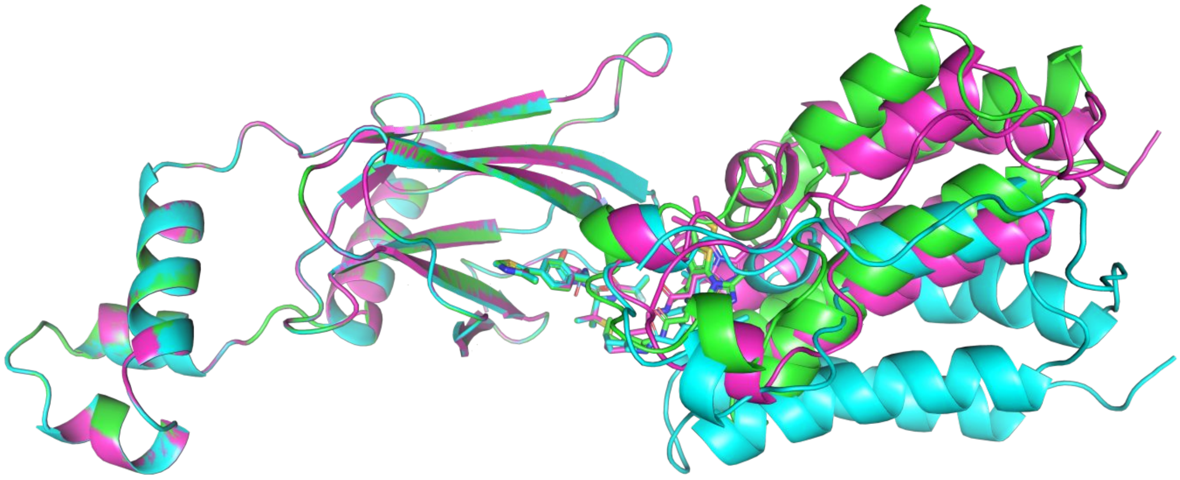

Figure S91. Overlay of the experimental ternary complex of 7ZNT (green), the model based on the closest conformer (cyan), and the model with the lowest pp_RMSD (magenta), aligned on the E3 ligase side.

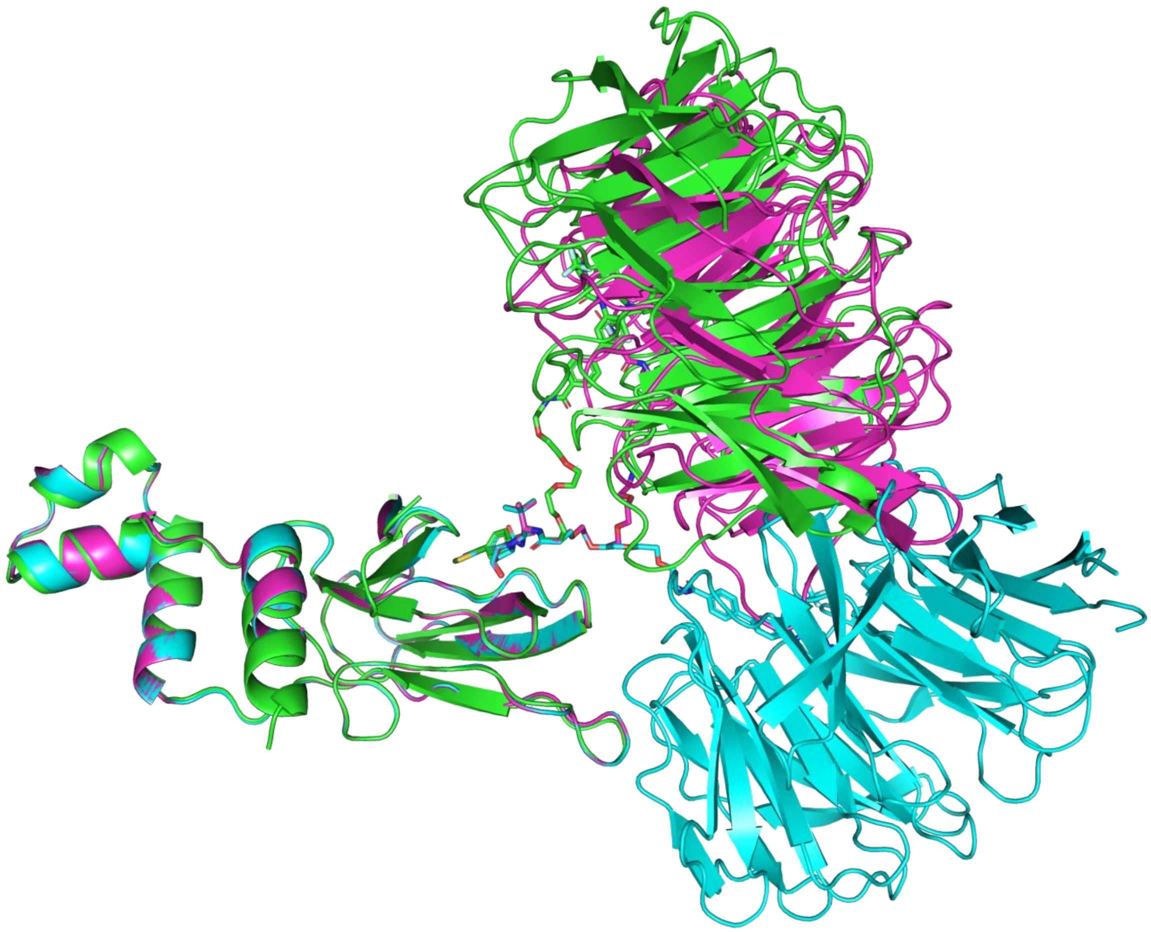

Figure S92. Overlay of the experimental ternary complex of 8BB2 (green), the model based on the closest conformer (cyan), and the model with the lowest pp_RMSD (magenta), aligned on the E3 ligase side.

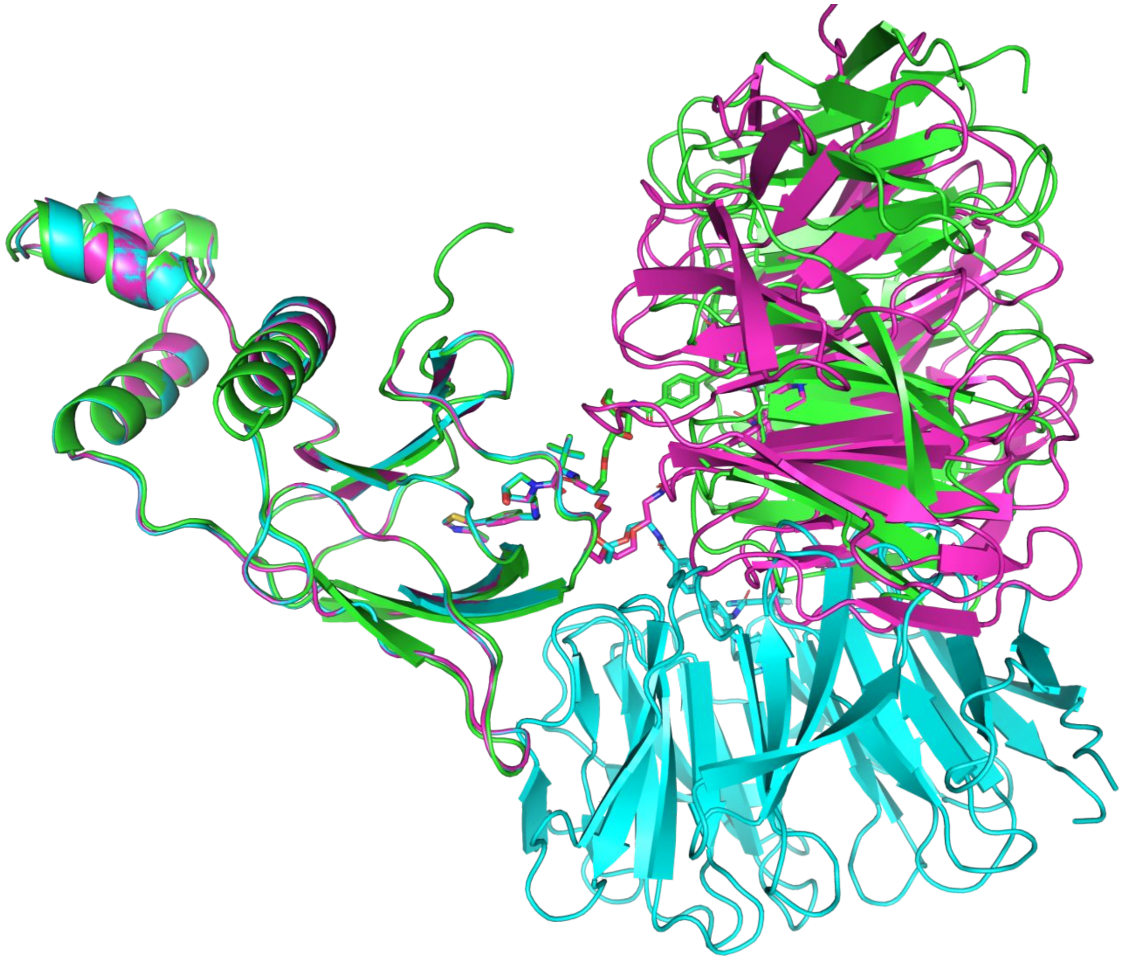

Figure S93. Overlay of the experimental ternary complex of 8BB3 (green), the model based on the closest conformer (cyan), and the model with the lowest pp_RMSD (magenta), aligned on the E3 ligase side.

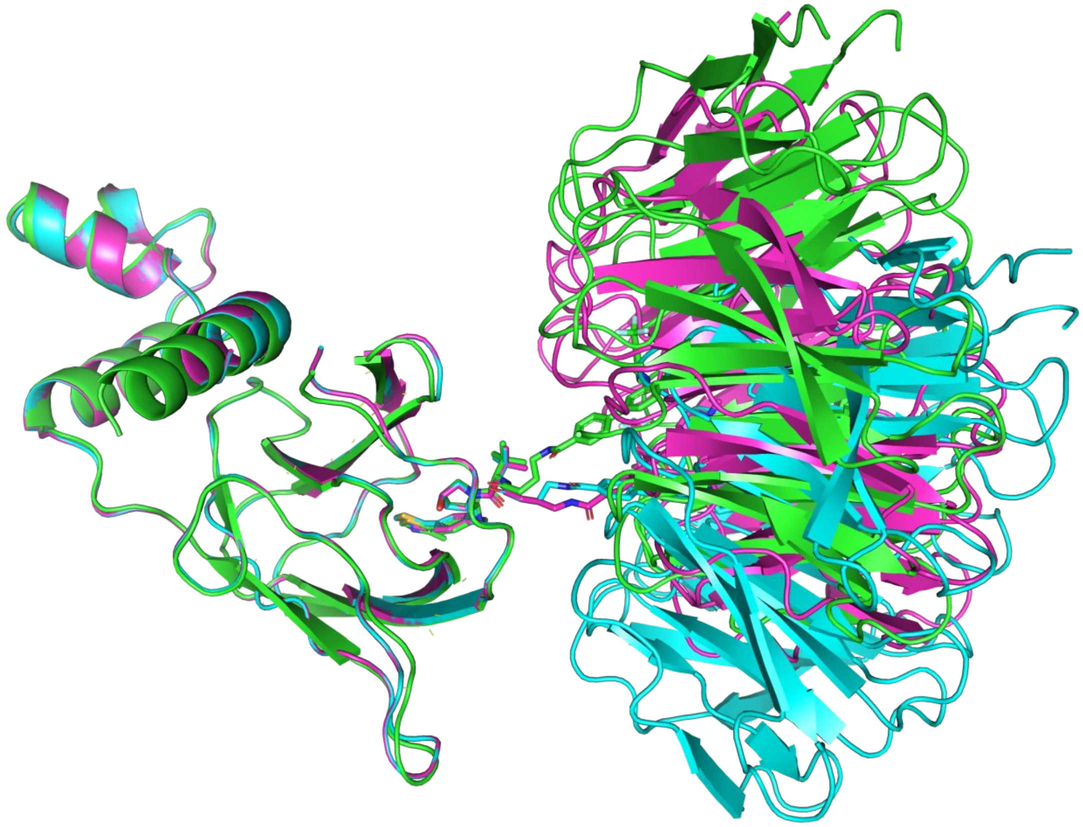

Figure S94. Overlay of the experimental ternary complex of 8BB4 (green), the model based on the closest conformer (cyan), and the model with the lowest pp_RMSD (magenta), aligned on the E3 ligase side.

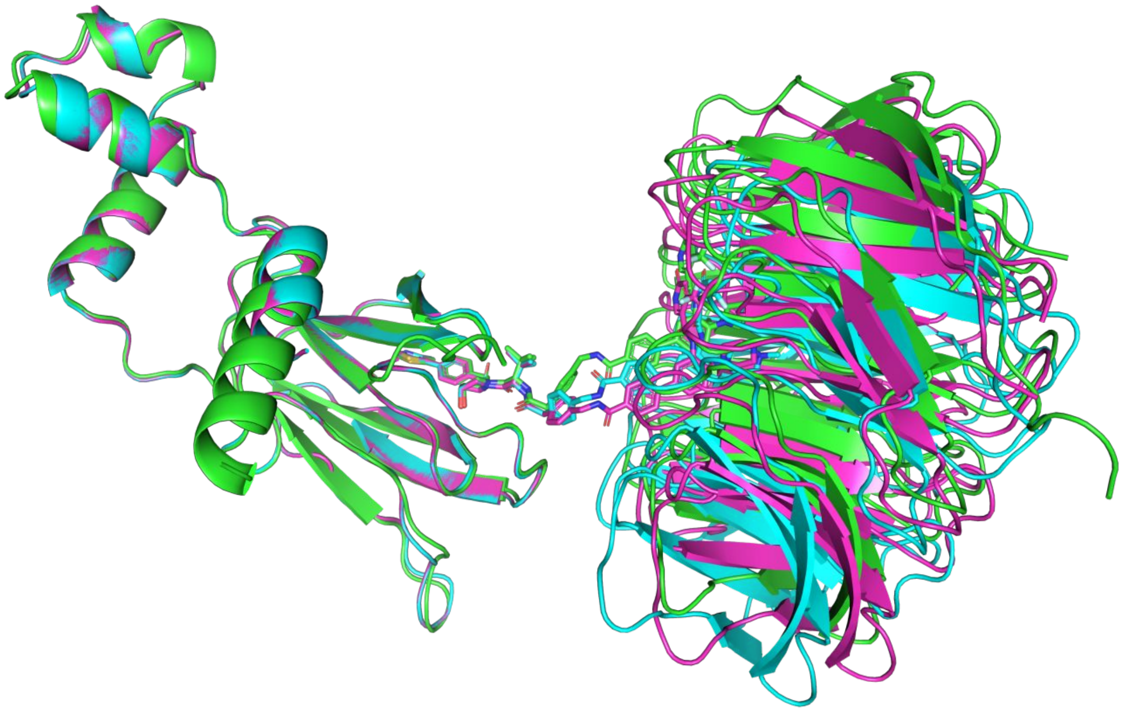

Figure S95. Overlay of the experimental ternary complex of 8BB5 (green), the model based on the closest conformer (cyan), and the model with the lowest pp_RMSD (magenta), aligned on the E3 ligase side.

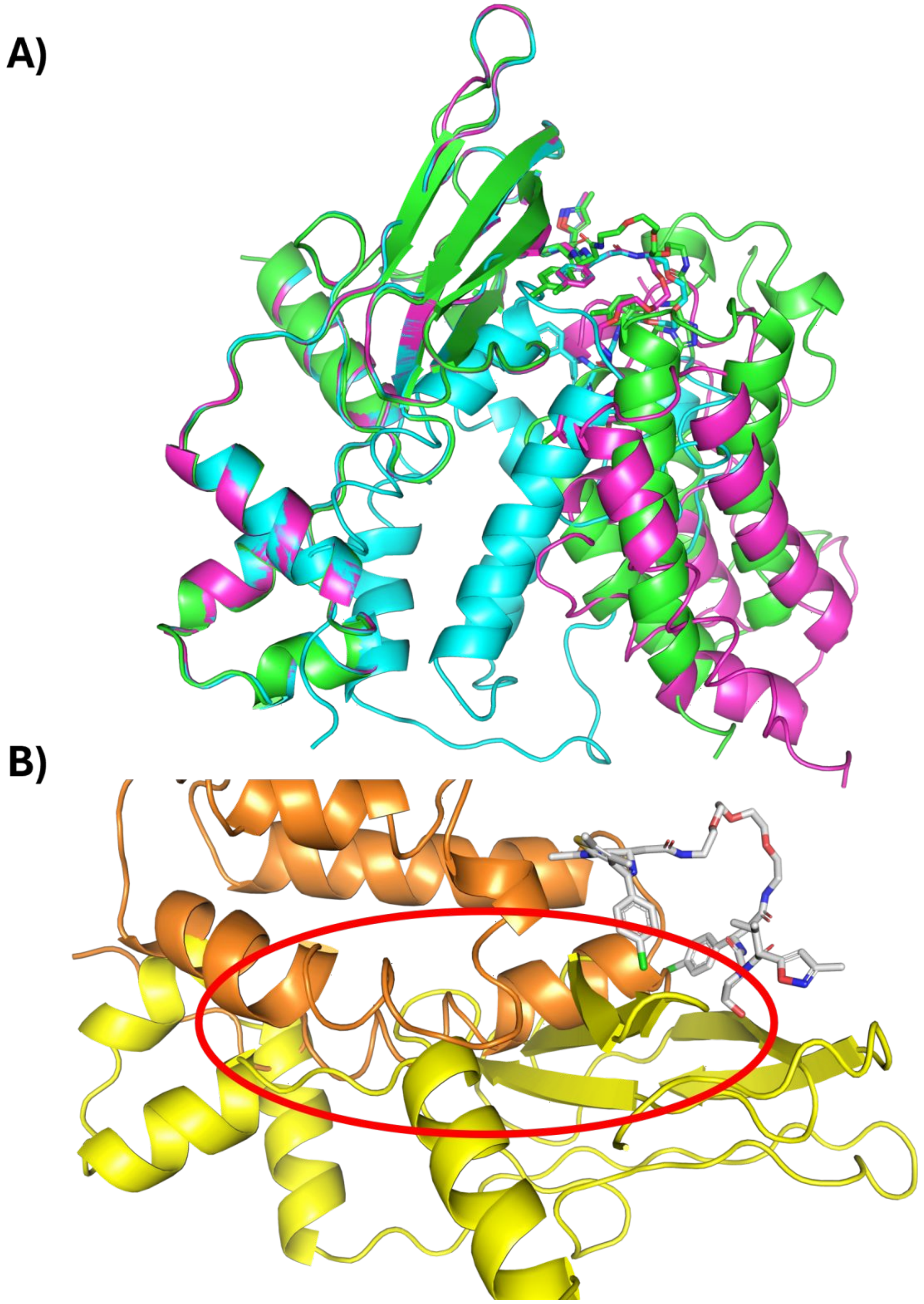

Figure S96. A) Overlay of the experimental ternary complex of 8BDS (green), the model based on the closest conformer (cyan), and the model with the lowest pp_RMSD (magenta), aligned on the E3 ligase side. **B)** Closest conformer-based model shown separately, with steric clashes circled in

red. The E3 ligase is shown in yellow, the POI in orange, and the PROTAC in light gray carbon sticks.

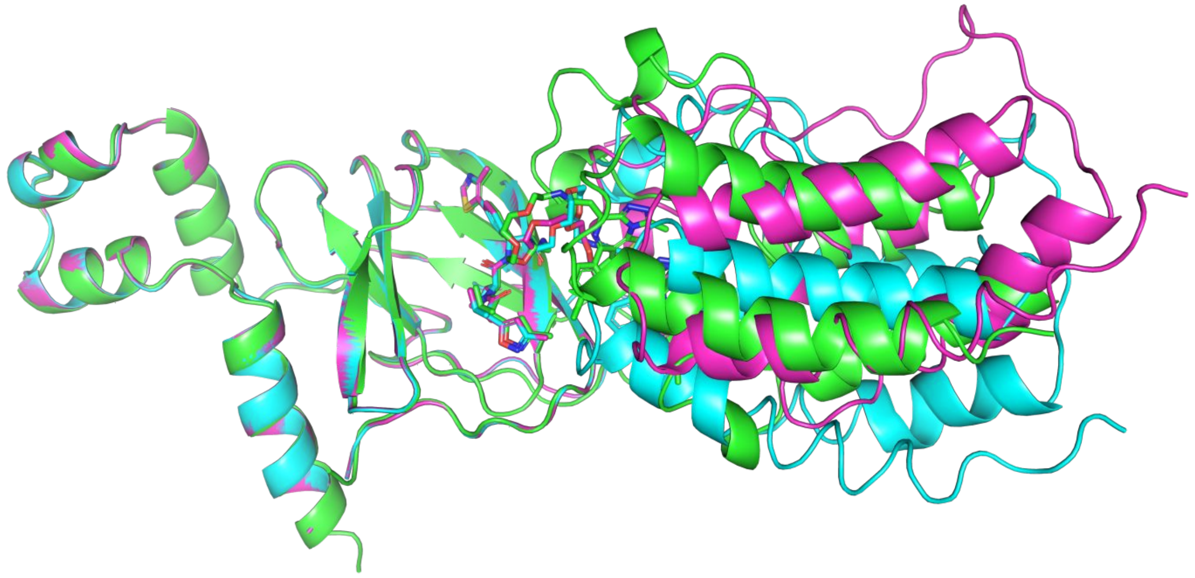

Figure S97. Overlay of the experimental ternary complex of 8BDT (green), the model based on the closest conformer (cyan), and the model with the lowest pp_RMSD (magenta), aligned on the E3 ligase side.

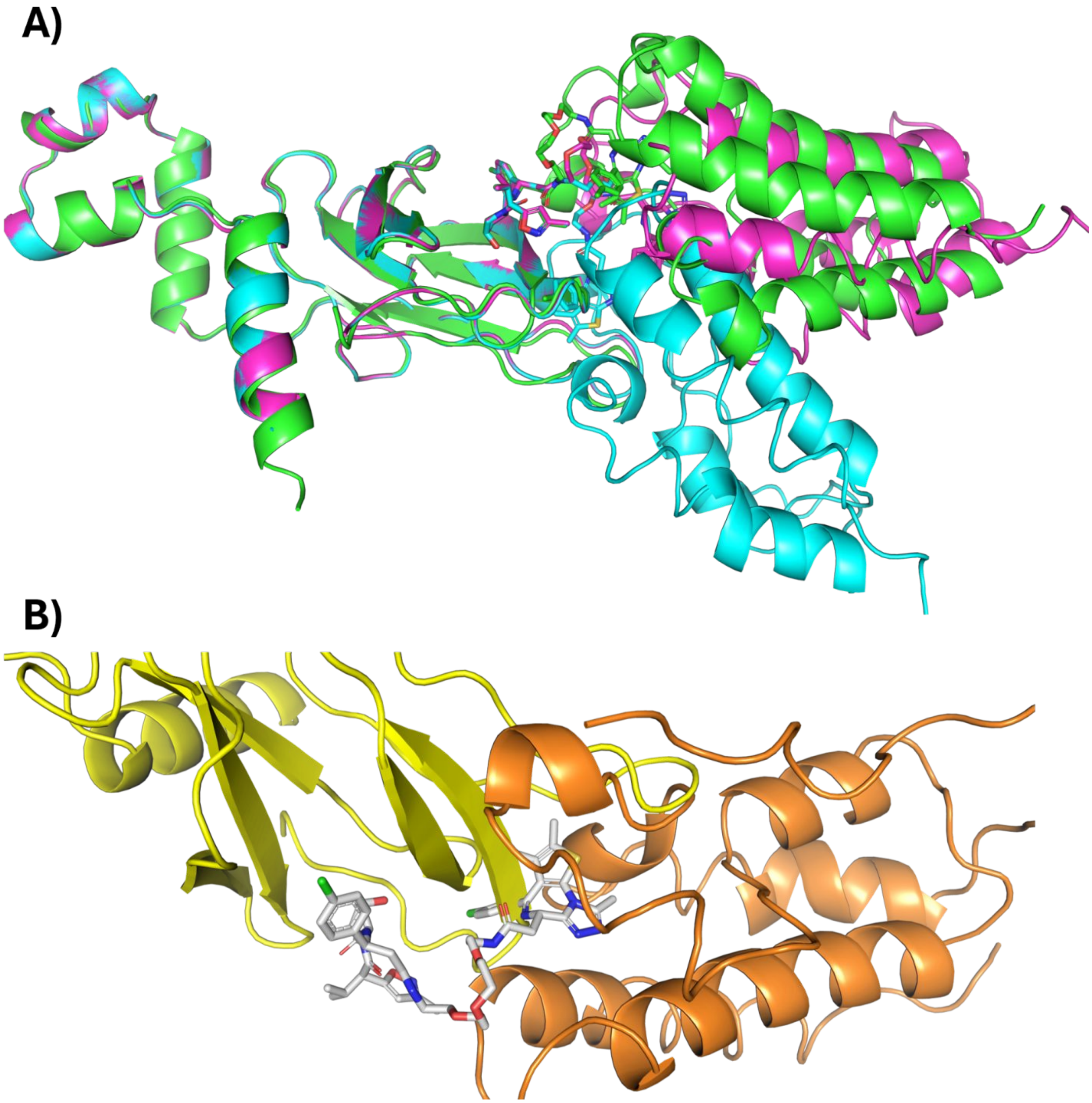

Figure S98. A) Overlay of the experimental ternary complex of 8BDX (green), the model based on the closest conformer (cyan), and the model with the lowest pp_RMSD (magenta), aligned on the E3 ligase side. **B)** Closest conformer-based model shown separately, with steric clashes circled in red. The E3 ligase is shown in yellow, the POI in orange, and the PROTAC in light gray carbon sticks.

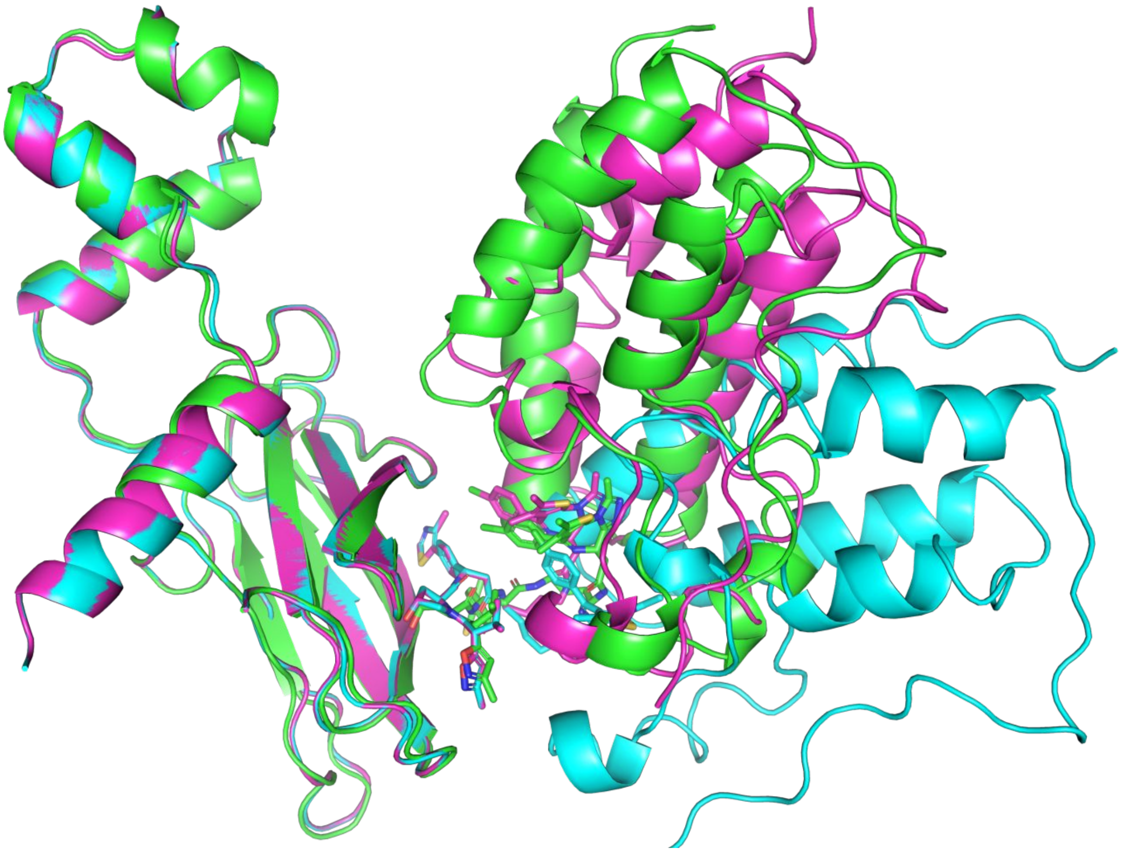

Figure S99. Overlay of the experimental ternary complex of 8BEB (green), the model based on the closest conformer (cyan), and the model with the lowest pp_RMSD (magenta), aligned on the E3 ligase side.

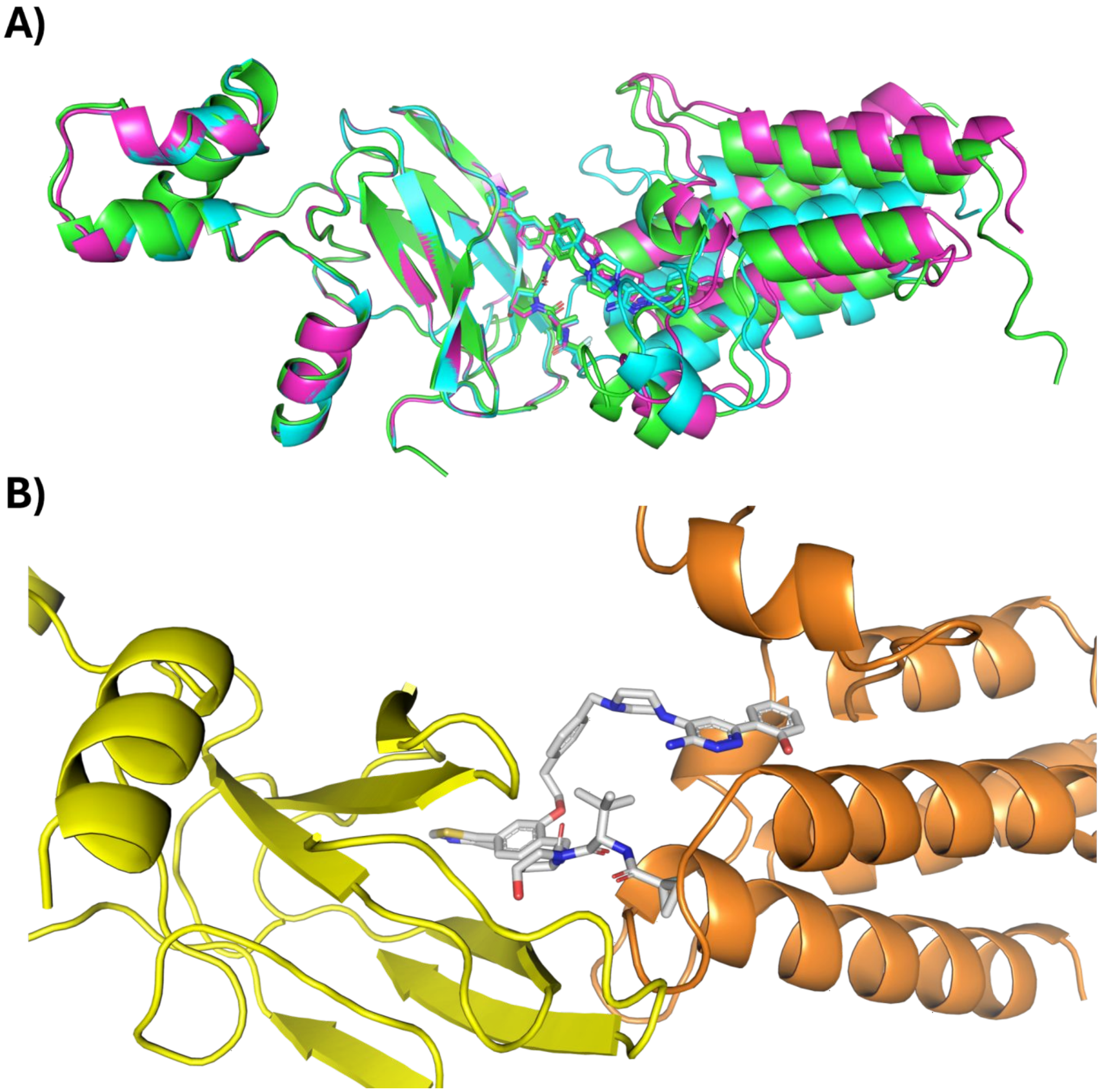

Figure S100. A) Overlay of the experimental ternary complex of 8G1P (green), the model based on the closest conformer (cyan), and the model with the lowest pp_RMSD (magenta), aligned on the E3 ligase side. **B)** Closest conformer-based model shown separately, with steric clashes circled in red. The E3 ligase is shown in yellow, the POI in orange, and the PROTAC in light gray carbon sticks.

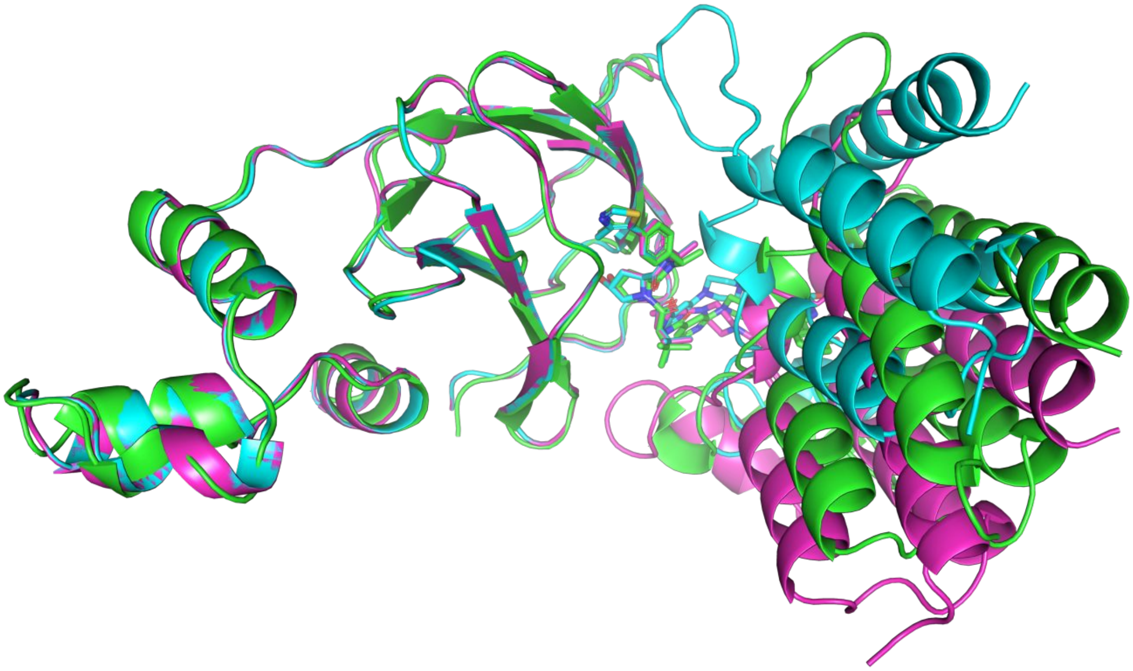

Figure S101. Overlay of the experimental ternary complex of 8G1Q (green), the model based on the closest conformer (cyan), and the model with the lowest pp_RMSD (magenta), aligned on the E3 ligase side.

Clustering and ensemble reduction for 6HAY

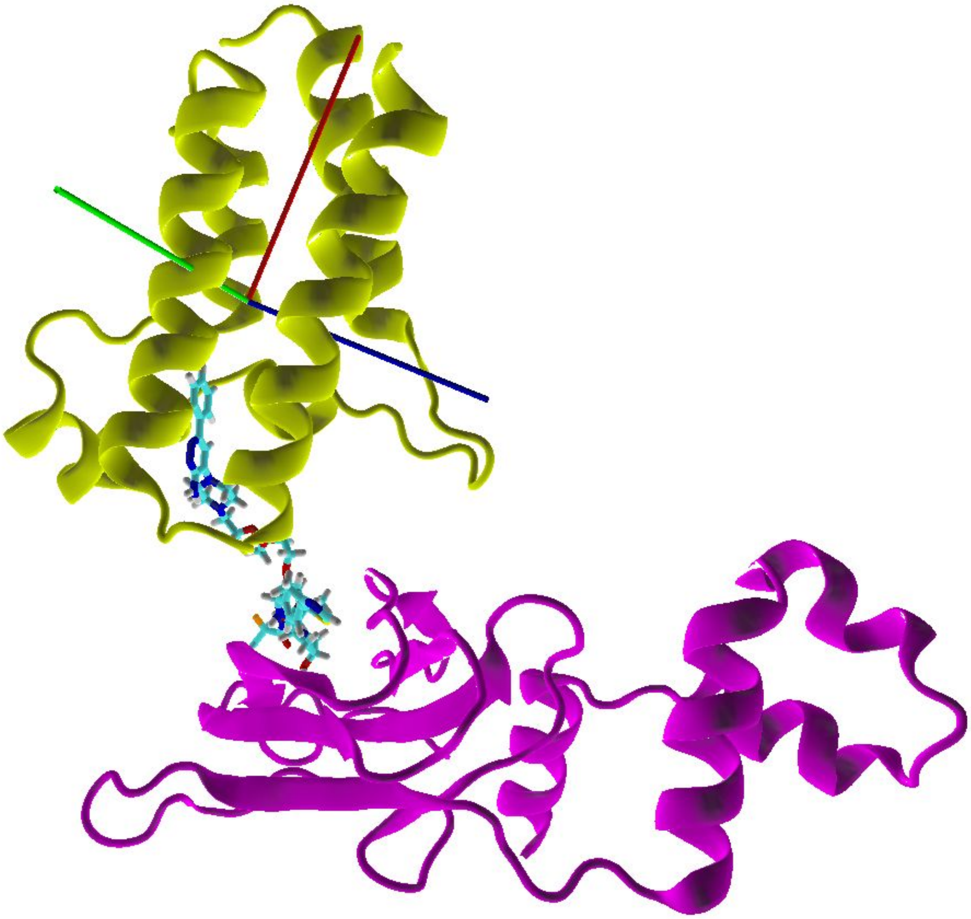

Figure S102. Visualization of the lowest clash-score model for 6HAY. The E3 ligase is shown in purple, the POI in yellow, and the PROTAC in cyan carbon sticks. The principal axes of the POI, derived from the moment of inertia tensor, are shown in red, blue, and green.

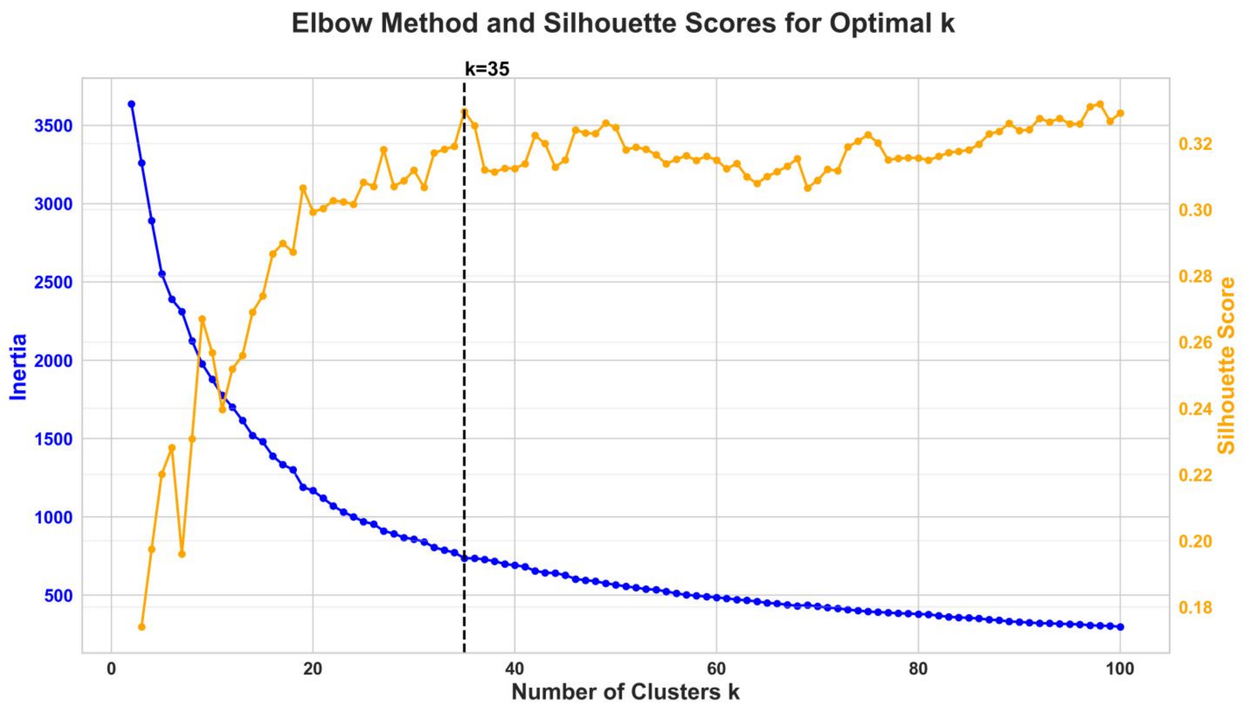

Figure S103. Combined plot of the Elbow method (blue) and Silhouette score (orange) used to determine the optimal number of clusters (k). The vertical dashed line indicates the selected k = 35.

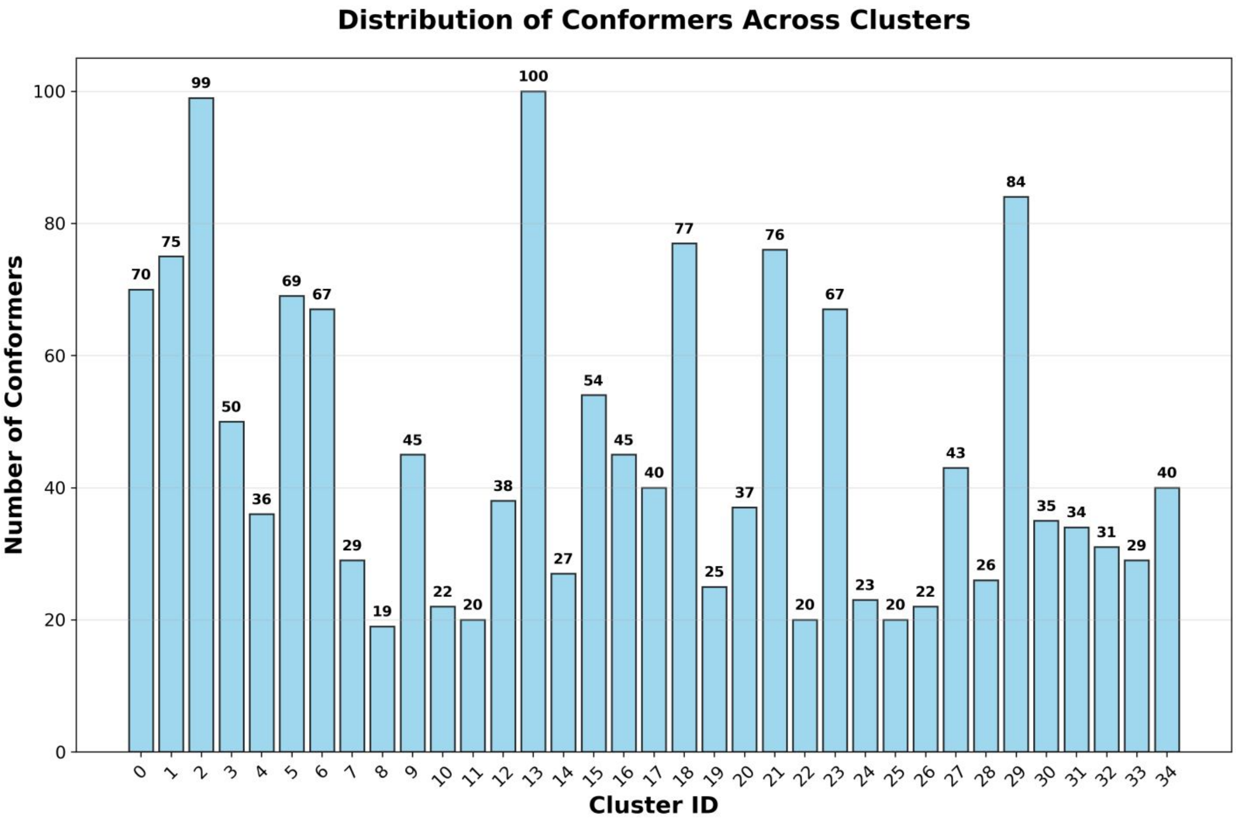

Figure S104. Bar plot showing the number of models assigned to each cluster after k-means clustering with k = 35. Clusters are shown on the x-axis; the y-axis indicates the number of models.

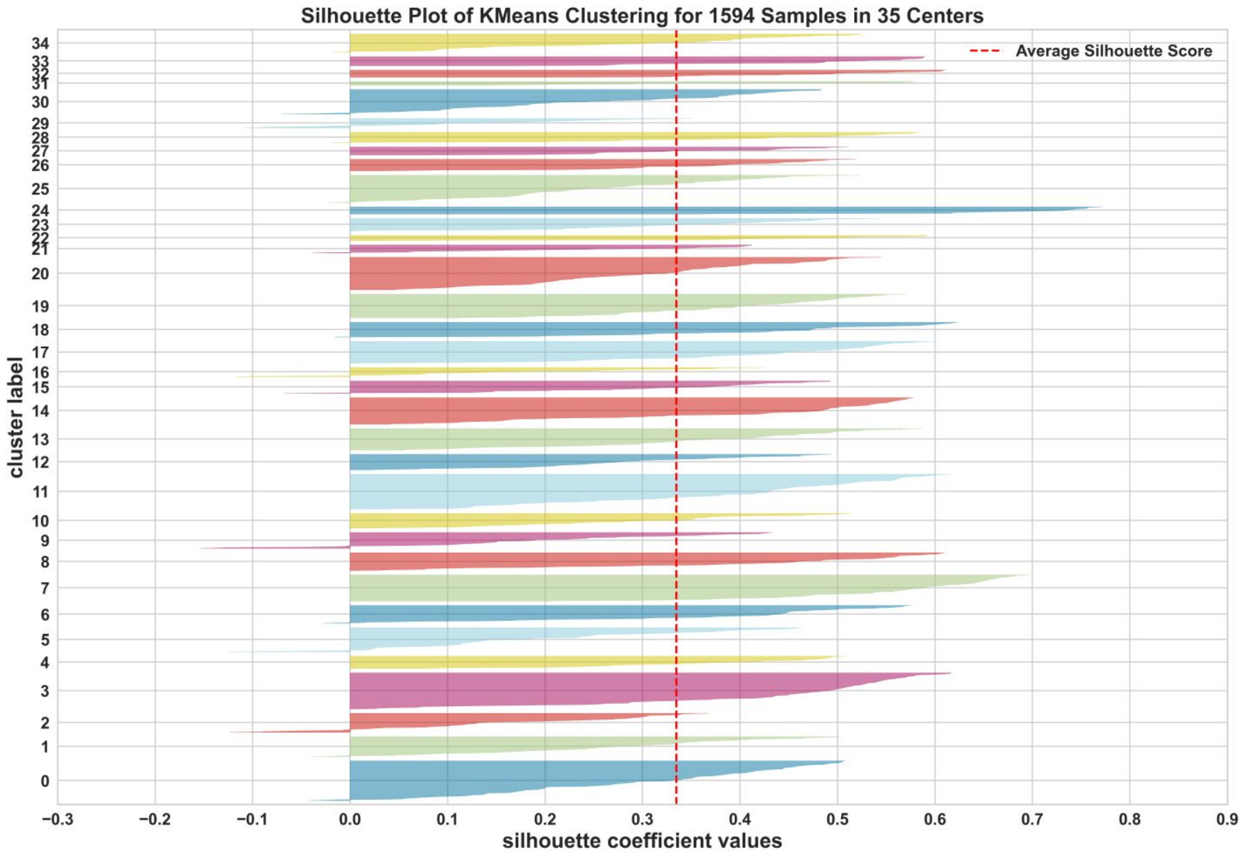

Figure S105. Distribution of Silhouette scores per cluster after clustering with k = 35. Positive values near 1 indicate well-separated clusters, while negative scores reflect some overlap; the red dashed line marks the average score.

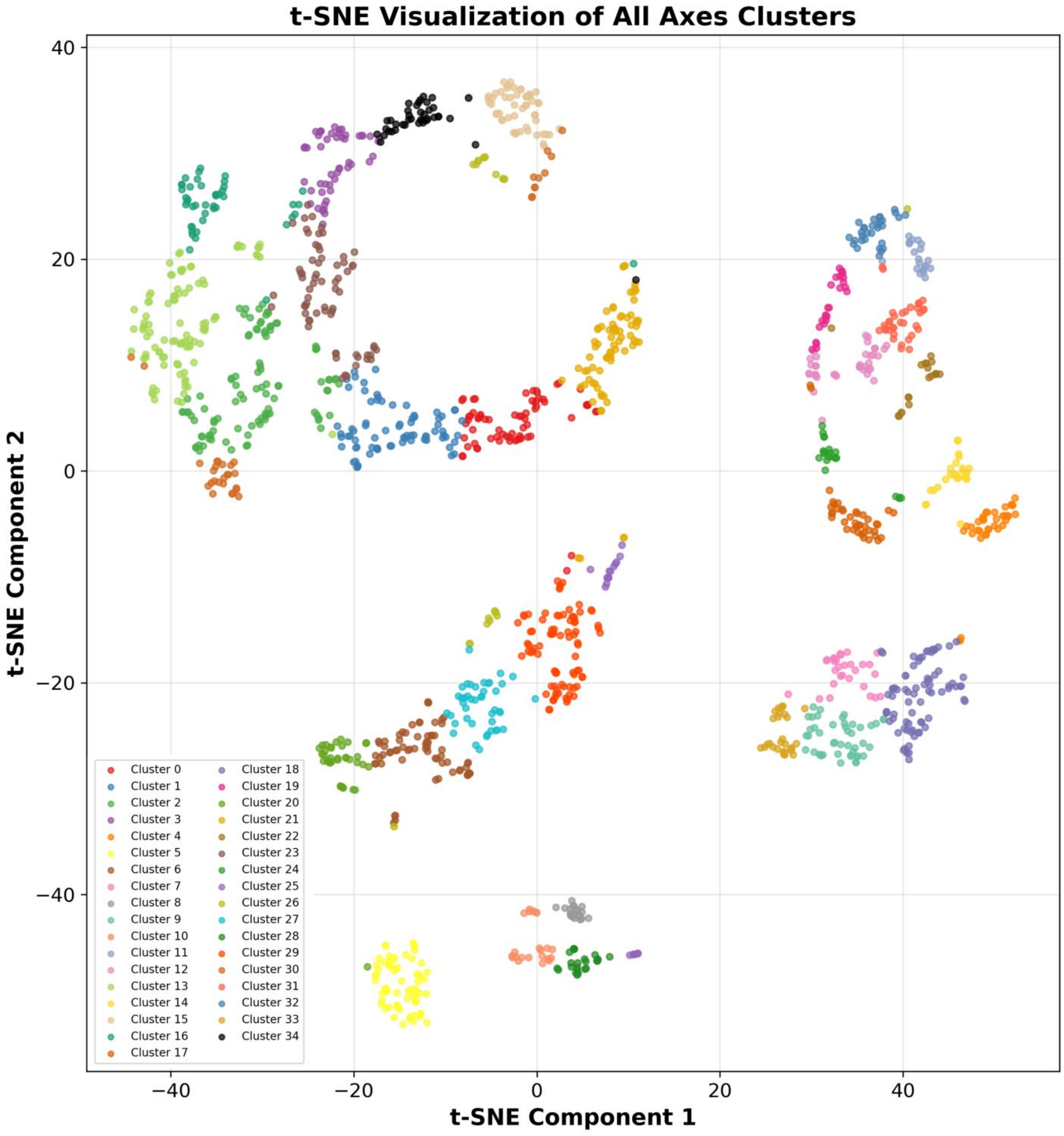

Figure S106. t-SNE plot (Component 2 vs. Component 1) of the clustered data, with points colored according to cluster assignment. The plot highlights the spatial relationships and separability of the clusters in reduced dimensional space.

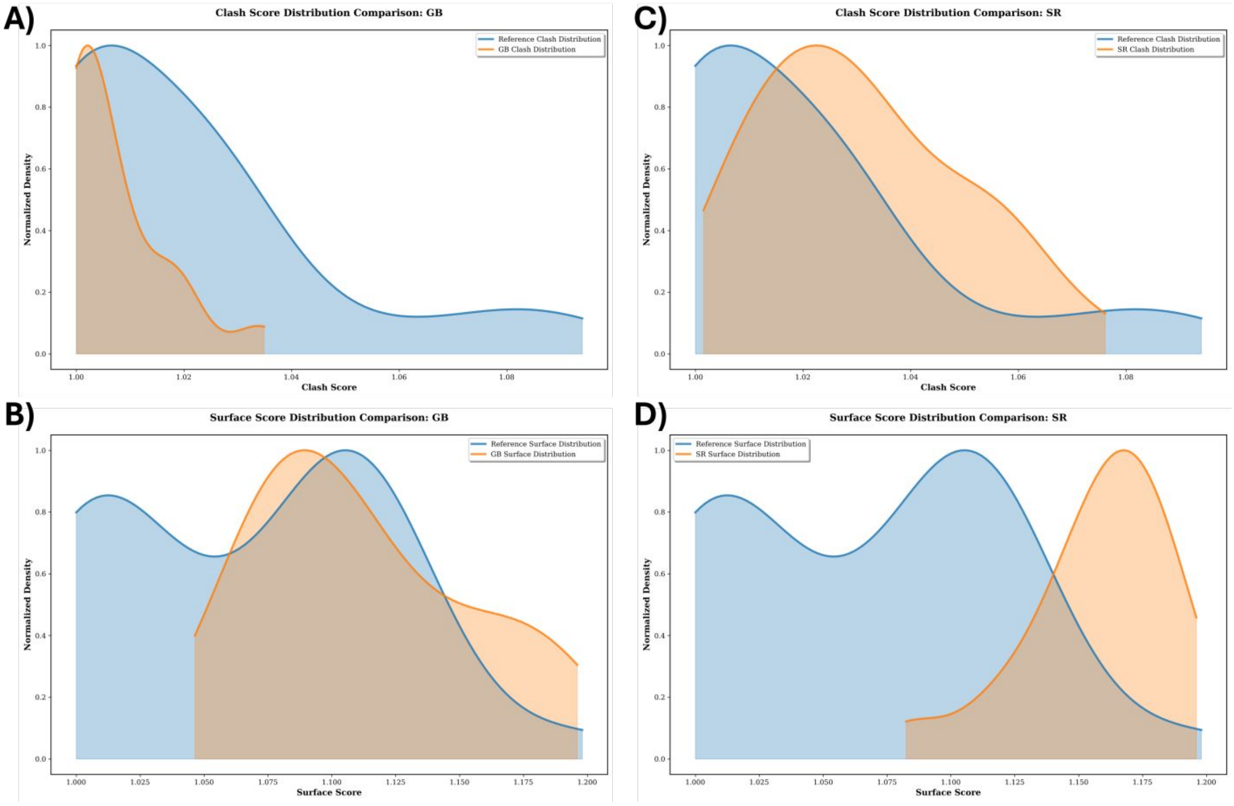

Figure S107. Kernel density estimation (KDE) plots comparing two model selection strategies: compromise score **(A, B)** and surface-to-clash ratio **(C, D)**. **A** and **C** show clash-score distributions; **B** and **D** show surface-score distributions. Blue curves represent experimental reference distributions; orange curves show the selected models.

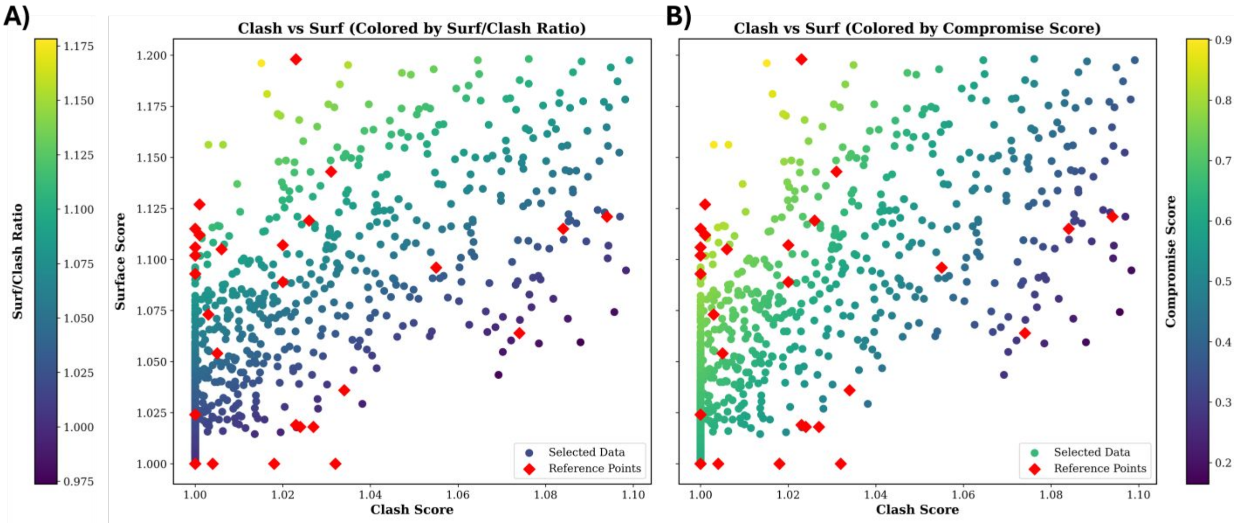

Figure S108. Surface-score vs. clash-score scatterplots for all models within the selected window. **A)** Points colored by surface-to-clash ratio. **B)** Points colored by compromise score. Experimental structures are shown as red diamonds.

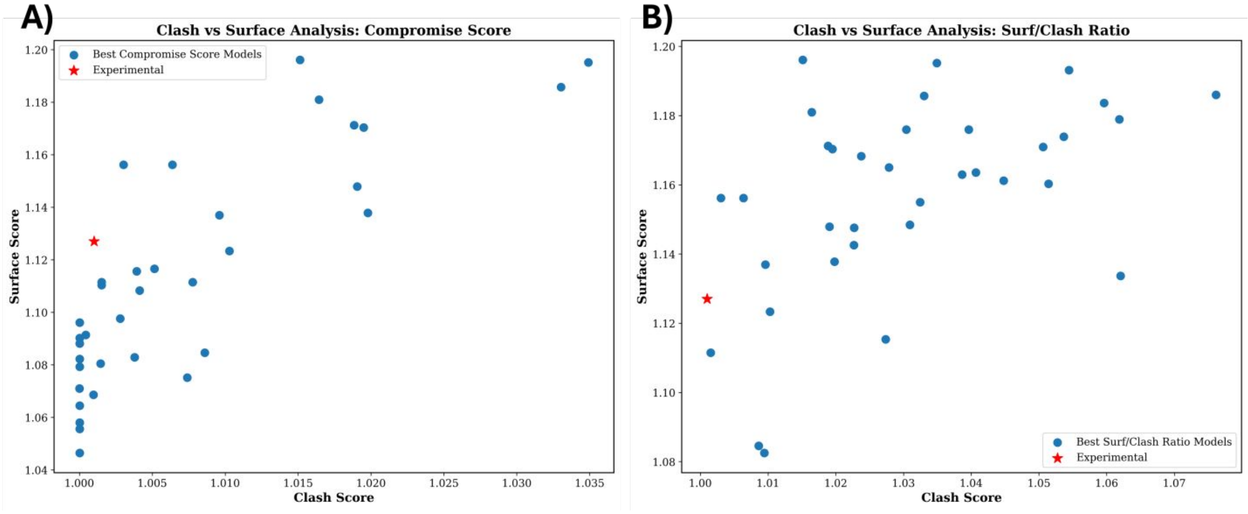

Figure S109. Surface-score vs. clash-score scatterplots for the final 35 selected models. **A)** Best models from each cluster by compromise score. **B)** Best models from each cluster by surface-to-clash ratio. The red star marks the experimental structure.
